# Flavone Cocrystals: A Comprehensive Approach Integrating Experimental and Virtual Methods

**DOI:** 10.1021/acs.cgd.4c00293

**Published:** 2024-05-06

**Authors:** Tom L. Petrick, Alexandra Grünwald, Doris E. Braun

**Affiliations:** Institute of Pharmacy, University of Innsbruck, Innrain 52c, 6020 Innsbruck, Austria

## Abstract

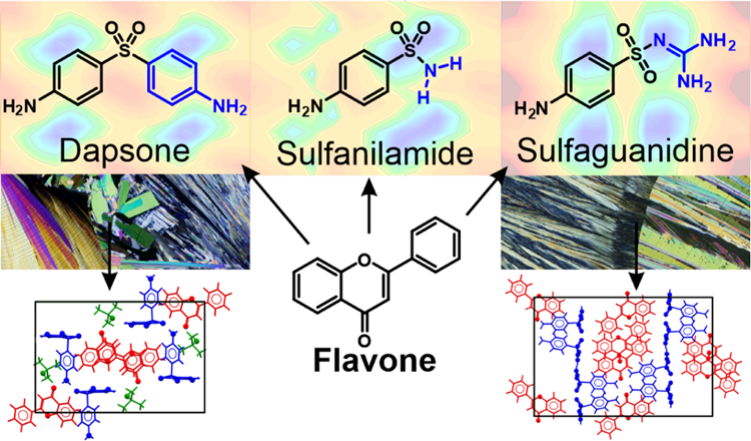

The dapsone/flavone cocrystal system served as a benchmark for both experimental and virtual screening methods. Expanding beyond this, two additional active pharmaceutical ingredients (APIs), sulfanilamide and sulfaguanidine, structurally related to dapsone were chosen to investigate the impact of substituents on cocrystal formation. The experimental screening involved mechanochemical methods, slurry experiments, hot-melt extrusion, and the contact preparation method. The virtual screening focused on crystal structure prediction (CSP), molecular complementarity, hydrogen-bond propensity, and molecular electrostatic potentials. The CSP studies not only indicated that each of the three APIs should form cocrystals with flavone but also reproduced the known single- and multicomponent phases. Experimentally, dapsone/flavone cocrystals **A**_**CC**_, **B**_**CC**_, **C**_**CC**_, and **D**_**CC**_ were reproduced, **C**_**CC**_ was identified as a nonstoichiometric hydrate, and a fifth cocrystal (**E**_**CC**_), a *t*-butanol solvate, was discovered. The cocrystal polymorphs **A**_**CC**_ and **B**_**CC**_ are enantiotripically related, and **D**_**CC**_, exhibiting a different stoichiometric ratio, is enthalpically stabilized over the other cocrystals. For the sulfaguanidine/flavone system, two novel, enantiotripically related cocrystals were identified. The crystal structures of two cocrystals and a flavone polymorph were solved from powder X-ray diffraction data, and the stability of all cocrystals was assessed through differential scanning calorimetry and lattice energy calculations. Despite computational indications, a diverse array of cocrystallization techniques did not result in a sulfanilamide/flavone cocrystal. The driving force behind dapsone’s tendency to cocrystallize with flavone can be attributed to the overall strength of flavone interactions in the cocrystals. For sulfaguanidine, the potential to form strong API···API and API···coformer interactions in the cocrystal is a contributing factor. Furthermore, flavone was found to be trimorphic.

## Abstract 2

The influence of the functional groups of structurally related APIs on cocrystallization with flavone was investigated. Employing a hybrid approach that incorporated both virtual and experimental cocrystal screening methods, seven distinct cocrystals were identified and thoroughly characterized. A meticulous investigation of the molecular interactions contributed to an enhanced understanding of cocrystal formation within this class of APIs.

## 1 Introduction

Multicomponent systems, such as cocrystals, offer a promising route to enhance the physicochemical properties of active pharmaceutical ingredients (APIs) while preserving their original composition.^1−3^ Potentially effective substances often fail to reach the market due to their low solubility and bioavailability, a problem that can be mitigated through cocrystallization.^2−4^ Pharmaceutical cocrystals gained notable significance in 2018 when the Food and Drug Administration recognized cocrystals as alternative crystal forms, alike to polymorphs.^5^ This allows drugs that have already been classified as safe to be reapproved in the form of a cocrystal. However, it is important to note that pharmaceutical cocrystals are primarily limited to coformers classified as “generally regarded as safe” (GRAS). The pool of over 370 potential GRAS coformers is expansive, making the exhaustive screening of all conceivable combinations a laborious task.^6^ Therefore, a deeper comprehension of the mechanisms underlying cocrystal formation is required.

Numerous methods can be employed to produce cocrystals, with common approaches including slurry-mediated transformation,^7^ antisolvent addition,^8^ solvent evaporation,^9^ cooling crystallization,^10^ and neat and liquid-assisted grinding.^11^ Furthermore, contact preparation,^12−14^ hot-melt extrusion,^15^ and freeze- and spray-drying techniques^16^ have been used successfully.^17,18^ The selection of a specific method can influence whether a metastable form or the thermodynamically stable phase is obtained.^17^

In addition to experimental approaches, computational methods play a pivotal role in cocrystal screening. Knowledge-based methods, like molecular complementarity (MC) and multicomponent hydrogen-bond propensity (MCHBP) utilize the Cambridge Structural Database (CSD) to predict cocrystallization based on a molecular and statistical basis, respectively.^19^ Alternatively, calculating the molecular electrostatic potential (MEP) surface of isolated molecules allows for estimating interaction energies in the solid state.^20^ Crystal structure prediction (CSP) generates numerous thermodynamically feasible crystal structures. In contrast to the other virtual screening methods, the molecular environment is considered in CSP studies. CSP, though powerful, becomes increasingly complex and computationally expensive as the size and number of molecules grow. As cocrystals are inherently more complex systems, *Z*′ = 1 and 1:1 stoichiometric ratio calculations are predominantly employed.^21^

**Figure 1:**
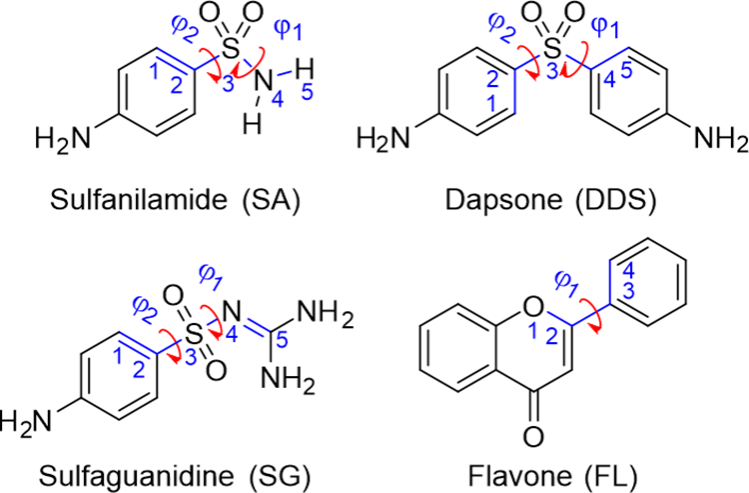
APIs and coformer (flavone) selected for the experimental and virtual cocrystal screen. The arrows indicate the torsion angles that have been considered in the virtual screenings. Key atoms are labeled.

This study encompasses both experimental and virtual cocrystal screening, focusing on three structurally related APIs and flavone (FL) as the coformer, as depicted in Figure 1. Dapsone (DDS), known to form cocrystals with flavone,^22,23^ served as the basis for the selection of the structurally related APIs—sulfanilamide (SA) and sulfaguanidine (SG).

DDS is a sulfone and aniline derivative similar to the sulfonamides in structure and action.^24^ The API is classified by the WHO as an essential medicine and is used to treat leprosy in combination with rifampicin.^24−26^ Dapsone is known to exist in five polymorphic forms (I,^27^ II,^28^ III,^29^ IV,^27^ V^27^), one hydrate,^30^ 15 solvates,^31,32^ and 16 cocrystals (with 5-nitroisophthalic acid;^33^ 1,3,5-trinitrobenzene;^33^ 1-pyridine-4yl-piperazine;^34^ 3,5-dinitro benzoic acid;^35^ urotropine;^36^ 1,3-benzothiazol-2(3H)-one;^22^ flavone;^22,23^ sulfanilamide;^22^ caffeine;^22^ 2,2′-bipyridne;^37^ 4,4′-bipyridine;^37,38^ caprolactam^38^). It has to be mentioned that four distinct cocrystals have been identified for the combination of DDS with FL, **A**_**CC**_, **B**_**CC**_, **C**_**CC**_, and **D**_**CC**_. Among these, **A**_**CC**_, **B**_**CC**_, and **C**_**CC**_ are described as 1:1 cocrystals, while D_CC_ is characterized as a 1:2 cocrystal.^22,23^

The antibiotic drug SA belongs to the group of sulfonamides, which competitively inhibit the dihydropteroate synthase by acting as substrate analogues of *p*-aminobenzoic acid.^39^ Despite its historical relevance, SA is now largely replaced by more advanced alternatives.^40^ Four polymorphic forms (α,^41^ β,^42^ γ,^43^ δ^44^) and one hydrate^45^ are documented in the literature. SA is known to form cocrystals with dapsone,^22^ caprolactam,^46^ antipyrine,^47^ oxymatrine,^48^ and sulfathiazole.^49^

SG, also a sulfonamide, acts locally in the gastrointestinal tract against bacterial pathogens and is commonly employed in animal treatment.^50^ Five polymorphic forms (I,^51^ II,^52^ III,^51^ IV,^53^ and V^51,53^), two monohydrate forms,^54,55^ nine solvates,^53^ and cocrystals with antipyrine,^56^ 1,10-phenantholine,^57^ thiobarbituric acid,^57^ 1,2-di(4-pyridyl)ethylene,^58^ 4-nitrobenzoic acid,^58^ 3-nitrobenzoic acid,^58^ and phenazine^58^ are reported.

Flavone, a member of the flavonoid class, exists in an anhydrous form^59^ and is known to form cocrystals with dapsone,^22,23^ naringenin,^60^ and diethylstilbestrol.^61^

The goal of this study was to investigate cocrystal formation and cocrystal stability, taking into consideration the diverse substitution patterns featured by the three selected APIs. Moreover, this research endeavored to bridge the gap between theory and experiment by conducting a rigorous examination of cocrystal formation, allowing us to gain deeper insights into the complex interplay of intermolecular forces governing this process. Through a careful comparison of predictive outcomes with experimental results, we thoroughly examined screening techniques and, conversely, validated the reliability and applicability of computational methods.

## 2 Materials and Methods

### 2.1 Materials

Sulfanilamide (form β; purity > 99%) obtained from Merck, dapsone (form III; purity 97%) from Alfa Aesar, sulfaguanidine (monohydrate I; purity > 99%) from Apoka, and flavone (form II; purity > 99%) from Acros were used as received. All solvents used in this study met analytical quality standards.

### 2.2 Experimental Cocrystal Screening

#### 2.2.1 Dry and Liquid-Assisted Grinding Experiments

Samples of 150 mg each, featuring molar ratios of 1:1, 1:2, and 2:1 (API/FL), were prepared. Approximately five drops of solvent [diisopropylether (DIPE), *n*-heptane, water, *t*-butanol (*t*-BuOH), isobutyl acetate (*i*-BuOAc)] were added in case of the liquid-assisted grinding experiments. The grinding process was executed using 5 mL stainless steel vessels, three 5 mm stainless steel balls, and a Retsch Vario MM 500 instrument (Retsch, Haan, Germany). Grinding was conducted at a frequency of 15 Hz for a duration of 60 min. At specific intervals (5, 10, 15, 30, 45, and 60 min), samples were retrieved and subjected to analysis using powder X-ray diffraction (PXRD).

#### 2.2.2 Slurry Experiments in Organic Solvents

Mixtures of 150 mg each, with molar ratios of 1:1, 1:2, and 2:1 (API/FL), were prepared. To these mixtures, 300–400 μL of saturated solution was added (DIPE, *n*-heptane, or water). The resulting slurry was stirred at temperatures cycling between 10 and 30 °C. At predefined intervals, samples were withdrawn and subjected to analysis using PXRD.

#### 2.2.3 Contact Preparation Method

Following the procedure described by Kuhnert–Brandstätter, contact preparation was used as an efficient method for distinguishing between eutectics and cocrystals.^14^

#### 2.2.4 Hot-Melt Extrusion

Mixtures containing 1.5 g of API and FL in a 1:1 molar ratio were prepared. The selection of extrusion temperatures was guided by the differential scanning calorimetry (DSC) curves of the mixtures (see section 1 in the Supporting Information). The extrusion process was carried out using the Extruder Hybrid ZE 5/12 HMI (Three-Tec GmbH, Seon, Switzerland) with a screw speed of 20 rpm.

### 2.3 Preparation of the Solid-State Forms

The nomenclature for both neat and cocrystal polymorphs lacks uniform standardization. In this work the polymorphs, hydrates, and solvates of the different compounds were labeled as follows: FL—**I**_**FL**_, **II**_**FL**_, and **III**_**FL**_; SA—**α**_**SA**_, **β**_**SA**_, **γ**_**SA**_, **δ**_**SA**_, and **H**_**SA**_ (hydrate); DDS—**I**_**DDS**_, **II**_**DDS**_, **III**_**DDS**_, **IV**_**DDS**_, **V**_**DDS**_, and **H**_**DDS**_ (hydrate); SG—**I**_**SG**_, **II**_**SG**_, **III**_**SG**_, **V**_**SG**_, and **H**_**SG**_ (hydrate); DDS/FL (cocrystal)—**A**_**CC**_, **B**_**CC**_, **C**_**CC**_, **D**_**CC**_, and **E**_**CC**_, with **C**_**CC**_ being a cocrystal hydrate and **E**_**CC**_ a cocrystal solvate; SG/FL (cocrystal)—**I**_**CC**_ and **II**_**CC**_. The subscripts indicate either the neat compound or a cocrystal (CC).

Flavone form I (**I**_**FL**_) could only be obtained in small quantities by slow solvent evaporation of a saturated (at RT) dimethyl carbonate solution, while form II (**II**_**FL**_) was obtained from 23 other solvent evaporation experiments (see section 8 of the SI). Melting **II**_**FL**_ and quench cooling of the melt to RT resulted in **III**_**FL**_.

Similarly, the SA polymorphs could be obtained via solvent evaporation from saturated solutions at RT. Form α (**α**_**SA**_) crystallized from water, form β (**β**_**SA**_) from butyl acetate, diethyl ether, dimethylformamide, 1,4-dioxane, and methanol. Form γ (**γ**_**SA**_) crystallized from dimethylacetamide.

The SG^53^ and DDS^27,62^ solid-state forms were prepared as previously reported.

To produce the DDS/FL cocrystal A (**A**_**CC**_), 20 mg of **C**_**CC**_ was weighed in and subjected to three heating and cooling cycles spanning from 20 to 109 °C, each with a rate of 2 °C per min. Following this, the sample was annealed isothermally at 108 °C for 3–5 days, resulting in **A**_**CC**_. Stirring a 1:1 molar ratio of DDS (527.75 mg) and FL (472.30 mg) in 3 mL of a saturated MeOH/water solution with a water activity of 0.23 or 0.85 for 1 week at RT resulted in **B**_**CC**_ or **C**_**CC**_, respectively. Stirring a 1:2 molar ratio mixture of DDS (358.42 mg) and FL (641.58 mg) in DIPE for 2 days within the temperature range of 10–30 °C led to **D**_**CC**_. Finally, **E**_**CC**_ was obtained in liquid-assisted grinding experiments. 736.20 mg of DDS, 763.70 mg of FL, and 50 drops of *t*-BuOH were ground at 15 Hz for 60 min.

SG/FL Form I (**I**_**CC**_) was produced by grinding a 1:1 mixture of SG (736.2 mg) and FL (763.70 mg). This mixture was processed with 50 drops of *t*-BuOH for 120 min at 15 Hz. **II**_**CC**_ could only be obtained through melt recrystallization. To achieve this, 873.15 mg of SG and 1126.80 mg of FL were mixed and recrystallized from the melt using a hot-melt extruder (see section 4 of the SI) at 185 °C and screw speed of 20 rpm.

### 2.4 Virtual Cocrystal Screening

The virtual methods described in Sections 2.4.2 (MC), 2.4.3 (MCHBP), and 2.4.4 (MEP) were performed for the low-energy conformers of the APIs and FL (see section 5 of the SI). The conformers were optimized with the B3LYP method and the 6-311++G(d,p) basis set using Gaussian09^63^

#### 2.4.1 Potential Energy Surface (PES) Scans

PES scans were performed at the PBE0/6-31G(d,p) level of theory using Gaussian09.^63^ Dihedral angles (Figure 1) were incrementally rotated in 30° steps, while maintaining the *p*-aminobenzene and guanidine groups planar (refer to section 5 of the SI).

#### 2.4.2 Molecular Complementarity (MC)

The parameters for molecular complementarity were calculated using the “Molecular Complementarity screening Wizard” tool built into Mercury 2023.3. This method relies on the evaluation of distinct molecular descriptors. Among these descriptors, three pertain to the dimensions of a rectangle encompassing the van der Waals volume. Additionally, the dipole moment and the proportion of N and O atoms within the molecules are considered.^64^

#### 2.4.3 Multicomponent Hydrogen-Bond Propensity (MCHBP)

The MCHBP analysis was performed with the CCDC’s “Multi_component_hydrogen_bond_propensity_report” python script and standard settings available on GitHub.^65^ This method relies on statistical analyses drawn from an extensive data set of structures within the CSD and involves the computation of scores for various functional group interactions. To assess the potential for cocrystal formation, the highest scores obtained from interactions between coformers, APIs, and between APIs and coformers are extracted. If the score of the prospective cocrystal exceeds that of the homomeric interactions, it suggests a favorable propensity for cocrystal formation.^19^

#### 2.4.4 Molecular Electrostatic Potential (MEP) Calculations

Multiwfn 3.7^65^ was employed to map the MEPs onto their 0.002 e^–^ Å^–3^ electron density isosurface. Using the determined local maxima and minima, the H-bond donor parameter (α) and H-bond acceptor parameter (β) were calculated, following the methodology outlined in earlier investigations.^20^ To assess the potential energy gain (Δ*E*_MEP_, kJ mol^–1^, with greater negativity indicating increased stabilization) associated with cocrystal formation, eq 1 was applied,
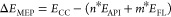
1with CC, API, and FL representing the cocrystal, API, and flavone, respectively, and *m* and *n* denoting the stoichiometric ratio.

The Δ*E*_MEP_ value cannot exceed 0, as in the absence of any preferred interaction, the energy of the cocrystal matches that of the initial substances. Consequently, it becomes challenging to establish a definitive threshold for determining cocrystal formation. In the existing literature, a probability criterion has been outlined for other compounds, suggesting that when Δ*E*_MEP_ is −11 kJ mol^–1^, there is approximately a 50% probability of cocrystal formation.^20^ Essentially, the lower the energy value, the more probable the formation of a cocrystal.

#### 2.4.5 Crystal Structure Prediction (CSP)

CSP calculations for the selected conformations (NH_2_ groups planar) were performed in the 59 most frequently encountered space groups using CrystalPredictor v2.0.1.^66,67^ For single-component systems, *Z*′ = 1 and *Z*′ = 2 rigid-body searches were executed, generating 250 000 and 500 000 structures for each search, respectively. In the case of multicomponent systems, calculations were carried out in *Z*′ = 1 with 500 000 generated structures for each search. The 9999 lowest-energy structures within a range of 10–25 kJ mol^–1^ relative to the global minimum were reoptimized using a distributed multipole model^68^ within DMACRYS.^69^ Subsequently, CrystalOptimizer v2.4.8^70,71^ was employed to optimize approximately 250 of the most stable structures (within a 13–25 kJ mol^–1^ energy range of the global minimum) for each system, allowing the dihedrals marked in Figure 1 to readjust in the crystal structure. The conformational energies and distributed multipoles^68^ used were calculated at the PBE0/6-31G(d,p) level (Gaussian09), and all other intermolecular forces were modeled in an atom–atom exp-6 form using the FIT potential.^69^

Following this flexible optimization, the energies were initially recalculated without optimization using the PBE-MBD* (DFT-*d*) method.^72−74^ Subsequently, the most stable crystal structures within a 10–15 kJ mol^–1^ energy range were then fully optimized (CASTEP v20.11^75^). For more details, refer to section 6 of the SI.

#### 2.4.6 Pairwise Intermolecular Energy Calculations

Crystal Explorer V17^76^ and Gaussian 16^77^ were employed to calculate the pairwise intermolecular energies using the B3LYP/6-31G(d,p) wave function.^78^ For this purpose, the PBE-MBD*-optimized structures (see previous section), employing a radius of 3.80 Å, were used to determine the electrostatic, polarization, dispersion, and repulsion energies.

### 2.5 PXRD and Structure Solution from PXRD

An X’pert Pro diffractometer (PANalytical, Almelo, Netherlands) equipped with a PIXcel1D detector and a Cu–K_α1,2_ radiation source, operating at 40 kV and 40 mA was used. The diffractometer was configured with a θ/θ coupled goniometer in a transmission geometry setup. The measurements covered a 2θ range spanning from 2 to 40°, with a step size of 0.013° and 40 s per step, or alternatively from 2 to 70° with a step size of 0.007° and 1600 s per step (multichannel mixed-signal) for structure determinations.

Structure solution was performed using the software programs DASH^79^ and TOPAS V7.^80^ The first 20 reflections were used for indexing the background-subtracted pattern with DICVOL. The absence of specific reflections was then statistically analyzed to determine the space group.^81,82^ Based on the unit cell’s volume and the determined space group, the number of molecules in the asymmetric unit could be established. The internal coordinate descriptions (*Z*-matrix) were derived from the gas-phase minima. Simulated annealing was employed to optimize the models directly in real space. O/N–H distances were normalized to 0.9 Å and C–H distances to 0.95 Å. For each structure, the optimization process involved 100 simulated annealing runs, consisting of 2 × 10^8^ moves per run. In this optimization, each API molecule had 6 external and 2 internal degrees of freedom, while FL had 6 external and 1 internal degree of freedom. The best solution was then minimized (PBE-MBD*), and the optimized structure, with N/O–H distances normalized to 0.9 Å and C–H distances to 0.95 Å, was used as the starting point for rigid-body Rietveld refinements.^83^ For more details, refer to section 7 of the SI.

### 2.6 Hot-Stage Microscopy (HSM)

An Olympus BH2 polarization microscope (Olympus, Austria) equipped with a Kofler hot-stage (Reichert, Austria) and an Olympus DP71 digital camera (Olympus, Austria) were used for the investigations.

### 2.7 Differential Scanning Calorimetry (DSC)

A DSC7 instrument (PerkinElmer, Norwalk, Connecticut) in conjunction with the Pyris 8.0 Software was used to analyze the samples. The samples were precisely weighed using a UM3 ultramicrobalance (Mettler, Greifensee, Switzerland). Approximately 2–5 mg of the sample were used, and the analysis employed a heating rate of 10 °C min^–1^, with a N_2_ purge gas flow of 20 mL min^–1^. Enthalpies were derived from hermetically sealed capsules with a minimum of three measurements, and error estimation was performed at a 95% confidence interval. The solvates were additionally measured with pierced lids. The calorimeter underwent calibration using benzophenone (48.0 °C) and caffeine (236.2 °C) for temperature calibration and indium (28.45 J g^–1^) for enthalpy calibration.

### 2.8 Thermogravimetric Analysis (TGA)

A TGA7 (PerkinElmer, Norwalk, Connecticut) controlled by the Pyris 8.0 Software was used to analyze approximately 3–5 mg of substance. Two-point calibration of the temperature was performed with ferromagnetic materials (Alumel and Ni, Curie-point standards, PerkinElmer). Heating rates of 5 and 10 °C min^–1^ were applied and N_2_ was used as purge gas (sample purge: 20 mL min^–1^, balance purge: 40 mL min^–1^).

### 2.9 Karl Fischer Titration

A C30 coulometric Karl Fischer titrator (Mettler Toledo, Schwarzenbach, Switzerland) was employed to analyze the water content of approximately 20–30 mg of material.

## 3 Results and Discussion

### 3.1 Experimental Cocrystal Screening

Cocrystal screening experiments for SA, DDS, and SG with FL were carried out using grinding and slurry experiments, as well as hot-melt extrusion and the contact preparation method. The products were initially analyzed using PXRD and IR, and the cocrystals were further characterized using HSM, DSC, and TGA.

#### 3.1.1 Contact Preparation Method

**Figure 2:**
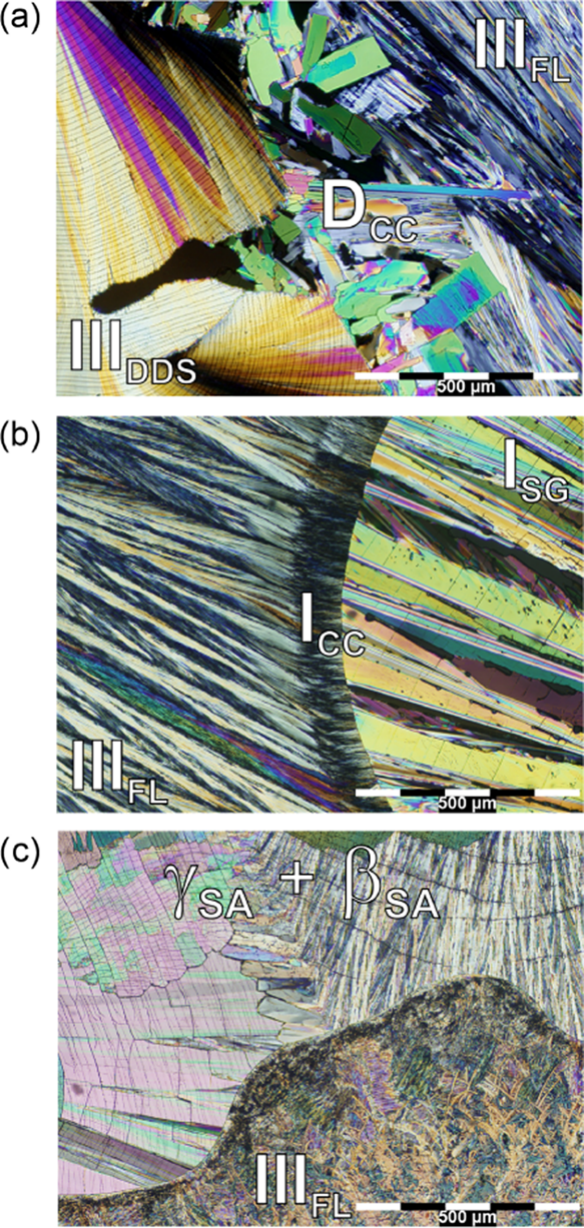
Contact preparation of (a) DDS/FL, (b) SG/FL, and (c) SA/FL; DDS—dapsone, FL—flavone, CC—cocrystal, SG—sulfaguanidine, SA—sulfanilamide.

Contact preparations of the API/coformer combinations were produced and cocrystallization was observed for SG and DDS, as illustrated in Figure 2. The contact preparation of DDS and FL (Figure 2a) resulted in the formation of the cocrystal **D**_**CC**_. Alongside the cocrystal formation, polymorphic transformations of DDS and FL were observed upon heating the sample (section 3.4 of the SI). FL exhibits a polymorphic transformation from **III**_**FL**_ to **II**_**FL**_ at approximately 70 °C, while DDS undergoes a transformation from **III**_**DDS**_ to **II**_**DDS**_ at around 80 °C.^28^ Upon further heating, the first eutectic event becomes apparent at 90 °C (**II**_**FL**_ and **D**_**CC**_). Following this, FL undergoes complete melting at 96 °C. The second eutectic event, involving **II**_**DDS**_ and **D**_**CC**_, occurs within a narrow temperature range of 105–106 °C. This second eutectic is somewhat challenging to distinguish because it almost coincides with the melting point of the cocrystal at 106 °C. During the initial contact preparation procedure, only traces of the cocrystal were observable at the contact zone. However, through subsequent cooling and reheating processes, the cocrystal zone can be significantly increased.

In the case of SG/FL, **II**_**CC**_ formed in small quantities (Figure 2b). Upon heating, the first eutectic occurs at 94 °C (**II**_**CC**_ and FL), immediately followed by the melting of FL at 96 °C. The second eutectic event becomes apparent at 173 °C, followed by the cocrystal’s melting point at 174 °C. The contact preparation also confirmed that FL is polymorphic (section 4 of the SI).

No cocrystallization was observed for SA/FL (Figure 2c). The SA polymorphs (β_SA_ and γ_SA_) crystallized concomitantly, with β_SA_ slowly transforming into γ_SA_, in agreement with literature reports.^84^ The eutectic temperature was determined as 76 °C. Subsequent reheating and cooling processes did not yield a cocrystal (section 2.4 of the SI).

#### 3.1.2 Dry and Liquid-Assisted Grinding Experiments (LAG)

The selection of solvents for the experimental cocrystal screening was based on considerations of solubility, polarity, and the potential to form H-bonds. Quick solubility tests were conducted using 30 different solvents (section 1.2 of the SI). Solvents that exhibited related solubility properties for both the API and the coformer were shortlisted. It has to be noted that no ideal solvent could be found. As a result, for the LAG experiments *n*-heptane was chosen as a nonpolar solvent, while DIPE and *i*-BuOAc were selected as H-bond acceptor solvents. Finally, *t*-butanol and water were chosen as solvents that feature H-bonding donor and acceptor groups. The dry and LAG experiments (Table 1) resulted in the formation of three DSS/FL (**C**_**CC**_, **D**_**CC**_, **E**_**CC**_) and two SG/FL cocrystals (**I**_**CC**_, **II**_**CC**_).

**Table 1:** API/FL Grinding Experiments^a^

	DDS/FL	DDS/FL	SG/FL	SG/FL	SG/FL	SA/FL	SA/FL	SA/FL
solvent	1:1	1:2	1:1	1:2	2:1	1:1	1:2	2:1
DIPE	**C**_**cc**_, H_DDS_*	**D**_**cc**_	**II**_**cc**_, I_SG_, II_FL_	**I**_**cc**_, **II**_cc_*	**I**_**cc**_, **II**_**cc**_, H_SG_	β_SA_, II_FL_	β_SA_, II_FL_	β_SA_, II_FL_
*n*-heptane	**D**_**cc**_, III_DDS_	**D**_**cc**_, III_DDS_, II_FL_	**I**_**cc**_, **II**_**cc**_, I_SG_, II_FL_	**I**_**cc**_, **II**_**cc**_, I_SG_, II_FL_	**I**_cc_*, **II**_**cc**_, I_SG_	β_SA_, II_FL_	β_SA_, II_FL_	β_SA_, II_FL_
water	**C**_**cc**_, H_DDS_*	**C**_cc_*, **D**_**cc**_, II_FL_*	H_SG_, II_FL_	H_SG_, II_FL_	H_SG_, II_FL_	β_SA_, H_SA_, II_FL_	β_SA_, H_SA_, II_FL_	β_SA_, H_SA_, II_FL_
*t*-BuOH	**E**_**cc**_	**E**_**cc**_, II_FL_	**I**_**cc**_	**I**_**cc**_, II_FL_	**I**_**cc**_, H_SG_	β_SA_, II_FL_	β_SA_, II_FL_	β_SA_, II_FL_
*i*-BuOAc	**D**_**cc**_, III_DDS_	**D**_**cc**_	**I**_**cc**_	**I**_**cc**_, II_FL_	**I**_**cc**_, H_SG_	β_SA_, II_FL_	β_SA_, II_FL_	β_SA_, II_FL_
dry	**D**_**cc**_, III_DDS_	**D**_**cc**_	**II**_cc_*, I_SG_, II_FL_	**II**_cc_*, I_SG_, II_FL_	**II**_**cc**_, I_SG_, II_FL_	β_SA_, II_FL_	β_SA_, II_FL_	β_SA_, II_FL_

a SA—sulfanilamide, DDS—dapsone, SG—sulfaguanidine, FL—flavone, H—hydrate, CC—cocrystal, *—small quantities detectable.

In the case of the DDS/FL experiments, the 1:2 cocrystal (**D**_**CC**_) predominantly formed and could be produced phase pure through dry and LAG using DIPE or *i*-BuOAc, when the correct molar ratio was used. However, when *n*-heptane was used, a grinding time of 60 min was insufficient for complete conversion into the cocrystal. This may be attributed to the low solubility of the substances in *n*-heptane. In the 1:2 grinding experiments involving water and *t*-BuOH, mixtures of cocrystals and/or educts were obtained, specifically **C**_**CC**_ and **E**_**CC**_, respectively. Notably, the new cocrystal (**E**_**CC**_) could be synthesized phase pure by starting with a 1:1 ratio and using *t*-BuOH, indicating the formation of a 1:1 cocrystal. However, upon storage under ambient conditions or with prolonged grinding, **E**_**CC**_ underwent desolvation into **A**_**CC**_ and **D**_**CC**_. It is worth noting that **C**_**CC**_ only formed in the presence of water or DIPE. The DIPE used for these experiments contained water as evidenced by the DDS anhydrate-to-hydrate transformation. For further details, see section 3 of the SI.

Two new cocrystals, **I**_**CC**_ and **II**_**CC**_, emerged when starting with SG and FL as reactants. The time-dependent LAG experiments (section 4 of the SI) revealed an interesting trend, where **II**_**CC**_ formed first and then transformed into **I**_**CC**_ (with the exception of *t*-BuOH). In the case of dry grinding experiments, no transformation was observed within the initial 60 min. Then, phase-pure **I**_**CC**_ could be obtained from the grinding experiments. Furthermore, the grinding results suggest a 1:1 stoichiometric ratio for these two cocrystals. However, it is evident that the 60 min duration and 15 Hz frequency employed were often insufficient for complete cocrystal formation. No cocrystallization was observed at all when water was used as the solvent.

In the case of SA/FL, no cocrystal formation was observed (see section 2 of the SI).

#### 3.1.3 Slurry Experiments in Organic Solvents and Water

The slurry experiments, conducted using a subset of the solvents mentioned in Section 3.1.2, reproduced the previously described DDS/FL cocrystals **C**_**CC**_ and **D**_**CC**_,^23^ and the two new SG/FL cocrystals **I**_**CC**_ and **II**_**CC.**_

When using molar ratios of 1:1 or 1:2 for DDS/FL in DIPE or *n*-heptane, **D**_**CC**_ formed along with an excess of reactants for most experiments. Conversely, using ratios of 1:2 phase pure **D**_**CC**_ was obtained in DIPE. **C**_**CC**_ was exclusively obtained from slurry experiments in water (section 3 of the SI), in agreement with the results from the grinding experiments. In the case of SG, both cocrystals were observed in the slurry experiments, although only **I**_**CC**_ could be obtained phase pure. Additionally, it was observed that **II**_**CC**_ transformed into **I**_**CC**_ over time. This observation suggests that at RT, **I**_**CC**_ is the stable polymorph of SG/FL (section 4 of the SI). Slurry experiments using SA did not yield any cocrystals (Table 2).

**Table 2:** API/FL Slurry Experiments^a^

	DDS/FL	DDS/FL	SA/FL	SA/FL	SA/FL	SG/FL	SG/FL	SG/FL
slurry	1:1	1:2	1:1	1:2	2:1	1:1	1:2	2:1
DIPE	**D**_**CC**,_ III_DDS_	**D**_**CC**_	β_SA_, II_FL_	β_SA_, II_FL_	β_SA_, II_FL_	**I**_**CC**_	**I**_**CC**_, II_FL_	**I**_**CC**_, H_SG_
*n*-heptane	**D**_**CC**,_ III_DDS_	**D**_**CC**_, II_FL_*	β_SA_, II_FL_	β_SA_, II_FL_	β_SA_, II_FL_	**I**_**CC**_, I_SG_, II_FL_	**I**_**CC**_, I_SG_, II_FL_	**I**_**CC**_, I_SG_, H_SG_
water	**C**_**C**C_, H_DDS_*, II_FL_*	**D**_**CC**_, **C**_CC_*	α_SA_, γ_SA_, H_SA_, II_FL_	H_SA_, II_FL_	β_SA_, γ_SA_, H_SA_, II_FL_	H_SG_, II_FL_	H_SG_, II_FL_	H_SG_, II_FL_

a SA—sulfanilamide, DDS—dapsone, SG—sulfaguanidine, FL—flavone, H—hydrate, CC—cocrystal, *—only small quantities obtained.

When using molar ratios of 1:1 or 1:2 for DDS/FL in DIPE or *n*-heptane, **D**_**CC**_ formed along with an excess of reactants for most experiments. Conversely, using ratios of 1:2 phase pure **D**_**CC**_ was obtained in DIPE. **C**_**CC**_ was exclusively obtained from slurry experiments in water (section 3 of the SI), in agreement with the results from the grinding experiments. In the case of SG, both cocrystals were observed in the slurry experiments, although only **I**_**CC**_ could be obtained phase pure. Additionally, it was observed that **II**_**CC**_ transformed into **I**_**CC**_ over time. This observation suggests that at RT, **I**_**CC**_ is the stable polymorph of SG/FL (section 4 of the SI). Slurry experiments using SA did not yield any cocrystals (Table 2).

In light of the fact that the presence of water led to the formation of **C**_**CC**_ for the DDS/FL system, the influence of water activity on cocrystal formation was explored. This investigation involved the use of various methanol/water mixtures^85^ and a 1:1 ratio of DDS/FL (section 3.2 of the SI). At lower water activities (≤0.43) **B**_**CC**_ forms, whereas at higher water activities (≥0.52) **C**_**CC**_ forms, indicating that **C**_**CC**_ might be a hydrate and that the critical water activity lies between 0.43 and 0.52.

#### 3.1.4 Hot-Melt Extrusion Experiments

By employing hot-melt extrusion with a 1:1 molar ratio of API/FL, it was possible to produce the cocrystals **D**_**CC**_, **I**_**CC**_, and **II**_**CC**_.

Processing of DDS/FL at temperatures above 86 °C resulted in the formation of **D**_**CC**_. Given the 1:2 molar ratio in the cocrystal, the coexistence of DDS and **D**_**CC**_ is unsurprising. Furthermore, the formation of the **III**_**FL**_ polymorph was seen in hot-melt extrusion experiments above 86 °C (section 3.3 of the SI). The SG/FL experiments showed an appearance of **I**_**CC**_ (as a mixture with **II**_**CC**_) above 100 °C. When the temperature was increased further (165 °C), **II**_**CC**_ formed predominantly (section 4.3 of the SI). Thus, HME can be used to produce phase pure **II**_**CC.**_ No cocrystallization occurred for SA/FL. Instead, depending on the extrusion temperature, either **β**_**SA**_ or **γ**_**SA**_ was present, with **γ**_**SA**_ forming at 120 °C and above (section 2.3 of the SI).

### 3.2 Virtual Cocrystal Screening

Potential energy scans were employed to derive the global and local energy minima of the three APIs and FL. Each two conformers were chosen for SA, SG, and FL, and one for DDS (section 5 of the SI).

#### 3.2.1 Molecular Complementarity

The analysis (SI section 5.3) suggests that SA and DDS are suitable to form cocrystals with FL, while SG is not a suitable candidate. The primary reason for deeming SG unsuitable is its guanidine function, which contains more N atoms than the respective groups of the other two APIs, causing it to exceed the threshold for polar atoms in the molecule.

Additionally, it is worth noting that one conformation of both SA and SG failed the Δ*S*/*L* (smallest/longest dimension). Thus, the choice of input conformation can significantly influence the result, i.e., fail or pass.

#### 3.2.2 Multicomponent Hydrogen-Bond Propensity

The likelihood of cocrystallization with FL versus single-component crystallization appears to be approximately equal for both SA and DDS. A multicomponent H-bond propensity score close to 0 does not favor one outcome over the other (section 5.5 of the SI). In contrast, for SG, a score of −0.25 was calculated, which suggests that cocrystallization with FL is unlikely. However, it has been experimentally demonstrated that both DDS and SG can form cocrystals with FL. The discrepancy may arise from the limitation that the MCHBP tool solely evaluates the strongest H-bonding interaction. However, the selected APIs feature numerous groups capable of forming strong interactions, which remain unaccounted for in this analysis. Therefore, Manian and coauthors have addressed this issue by modifying the tool to an “integrated” HBP method to account for the competitive probabilities of H-bonding.^86^

#### 3.2.3 Molecular Electrostatic Potential Calculations

**Figure 3:**
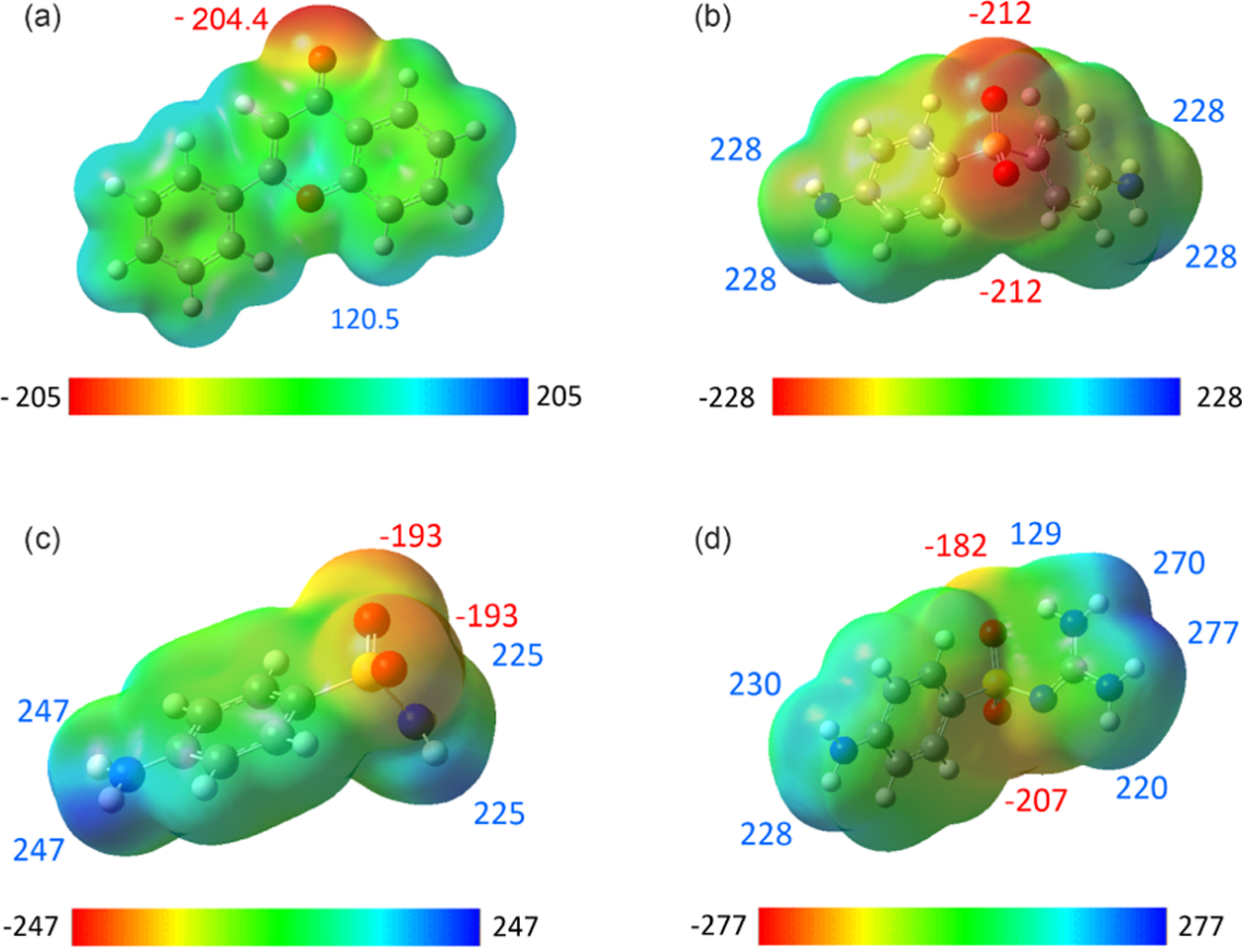
Molecular electrostatic potential maps of (a) FL, (b) DDS, (c) SA, and (d) SG. Note that only the strongest partial negative and positive potentials are shown, and energy values are given in kJ mol^–1^.

Figure 3 shows the MEP maps of the APIs and flavone, with red areas representing partial negative regions and blue areas representing partial positive regions. FL, a relatively flat molecule, features two oxygen atoms in its chromone ring. The ketone oxygen carries a partial negative charge. Conversely, the other oxygen is somewhat shielded by surrounding hydrogens, leading to a partial positive charge on this side of the molecule. The DDS hydrogen atoms of the amine group create a partial positive region, while the oxygens of the sulphonyl group form a partial negative region. SA and SG share structural similarities with dapsone. In the case of SA, one of the *p*-substituted aniline groups is replaced by NH_2_, essentially preserving the potential characteristics. In SG, the guanidine group features four positive regions. Additionally, the guanidine group, which lacks the symmetry of the functional groups seen in the other APIs, also affects the potential of the sulphonyl group’s O atoms.

Energy gain estimations upon cocrystallization, represented as Δ*E*_MEP_, were calculated using eq 1 for the stoichiometric ratios of 1:1, 1:2, and 2:1 (API/FL). Regardless of the API used, similar results were obtained. Specifically, all 1:1 and 2:1 combinations exhibited energy gain values falling in the range of −12 to −15 kJ mol^–1^, while the 1:2 combinations showed values ranging from −22 to −25 kJ mol^–1^. Therefore, the 1:2 stoichiometric ratio appears to be the preferred one, and all three APIs should be capable of forming cocrystals with FL. For more details, refer to section 5.4 of the SI.

### 3.3 Computationally Generated Crystal Energy Landscapes

Single-component crystal energy landscapes were computed for SA and FL to complement the crystal energy landscapes previously generated for DDS^62^ and SG.^53^ It should be highlighted that the energy landscape for DDS was recalculated using the methodology outlined in Section 2.4.5. All of the experimental single-component polymorphs that fell within the criteria of the search were successfully located in the crystal energy landscapes.

#### 3.3.1 Single-Component Lattice Energy Landscapes

**Figure 4:**
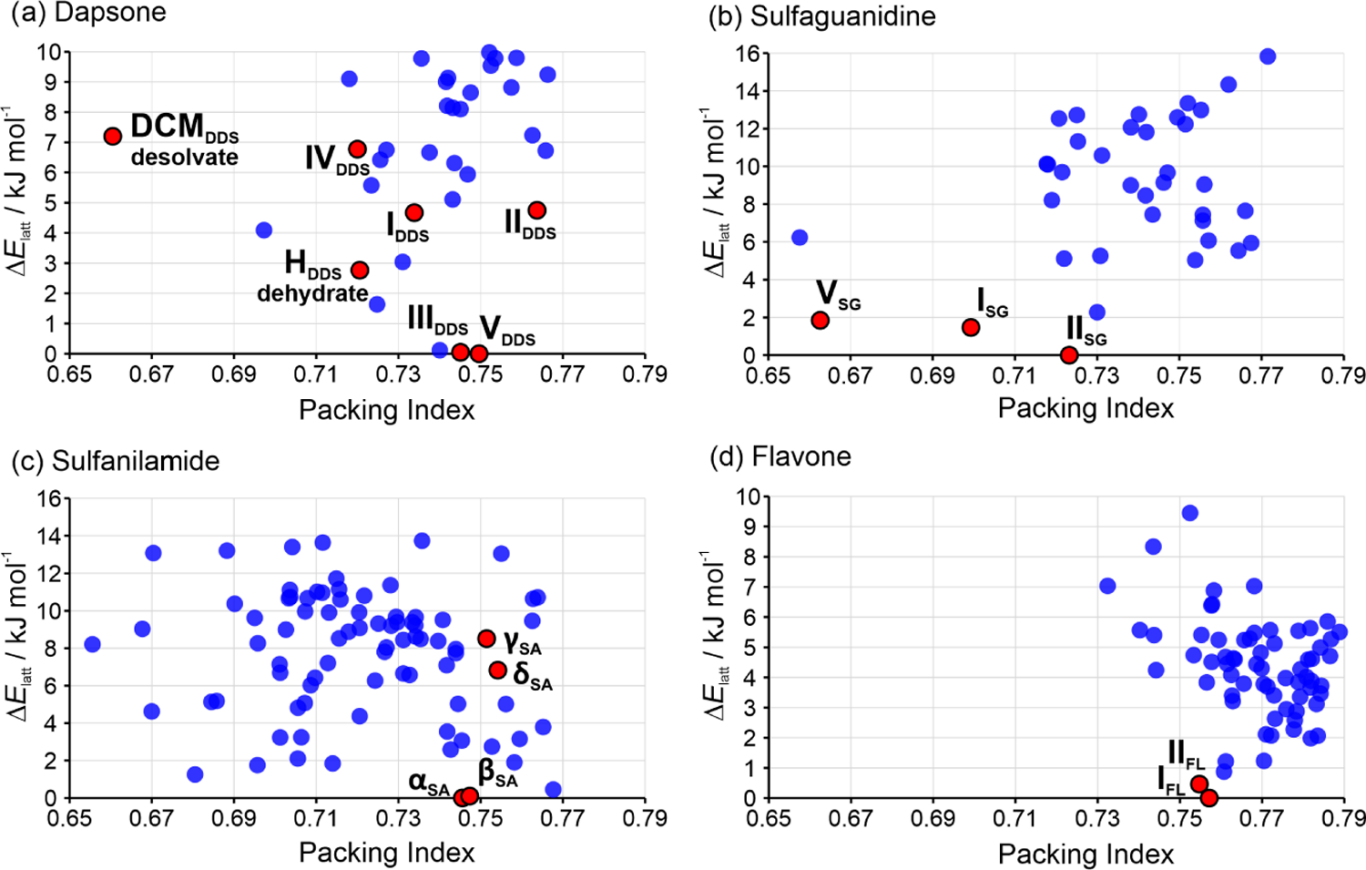
Computationally generated crystal energy landscape of (a) DDS, (b) SG, (c) SA, and (d) FL. The experimental forms are labeled and marked in red.

The DDS lattice energy landscape reveals **V**_**DDS**_ as the global minimum structure, with **III**_**DDS**_ as the second most stable structure. All other characterized polymorphs (**I**_**DDS**_, **II**_**DDS**_, and **IV**_**DDS**_), the isostructural **H**_**DDS**_ dehydrate,^62^ as well as a theoretical isostructural DCM desolvate,^31^ were found within a range of 10 kJ mol^–1^ of the global minimum (Figure 4a). As recently reported by us, the three structurally characterized SG polymorphs (**I**_**SG**_, **III**_**SG**_, and **V**_**SG**_) correspond to the three lowest-energy structures in the crystal energy landscape (Figure 4b).^53^

The SA search reveals that all four known polymorphs were found among the computationally generated structures, with **α**_**SA**_ being the global minimum structure followed by **β**_**SA**_ (nearly equi-energetic). Additionally, **γ**_**SA**_ and **δ**_**SA**_ were found within a range of 5 kJ mol^–1^ of the global minimum (Figure 4c). Toscani et al. reported the experimental stability order of the polymorphs as **β**_**SA**_ (most stable) > **α**_**SA**_ > **γ**_**SA**_, with an energy difference of only 0.2 kJ mol^–1^ between **β**_**SA**_ and **α**_**SA**_.^87,88^ Thus, these two structures are very close in terms of energy, a conclusion that aligns with our calculations. Concerning FL, the structurally characterized form, **I**_**FL**_ was identified as the global minimum structure (Figure 4d). The second most stable structure, which was found to be 0.5 kJ mol^–1^ less stable, was identified through structural and PXRD comparisons as **II**_**FL**_.

#### 3.3.2 Sulfanilamide/Flavone PES and Lattice Energy Landscape

**Figure 5:**
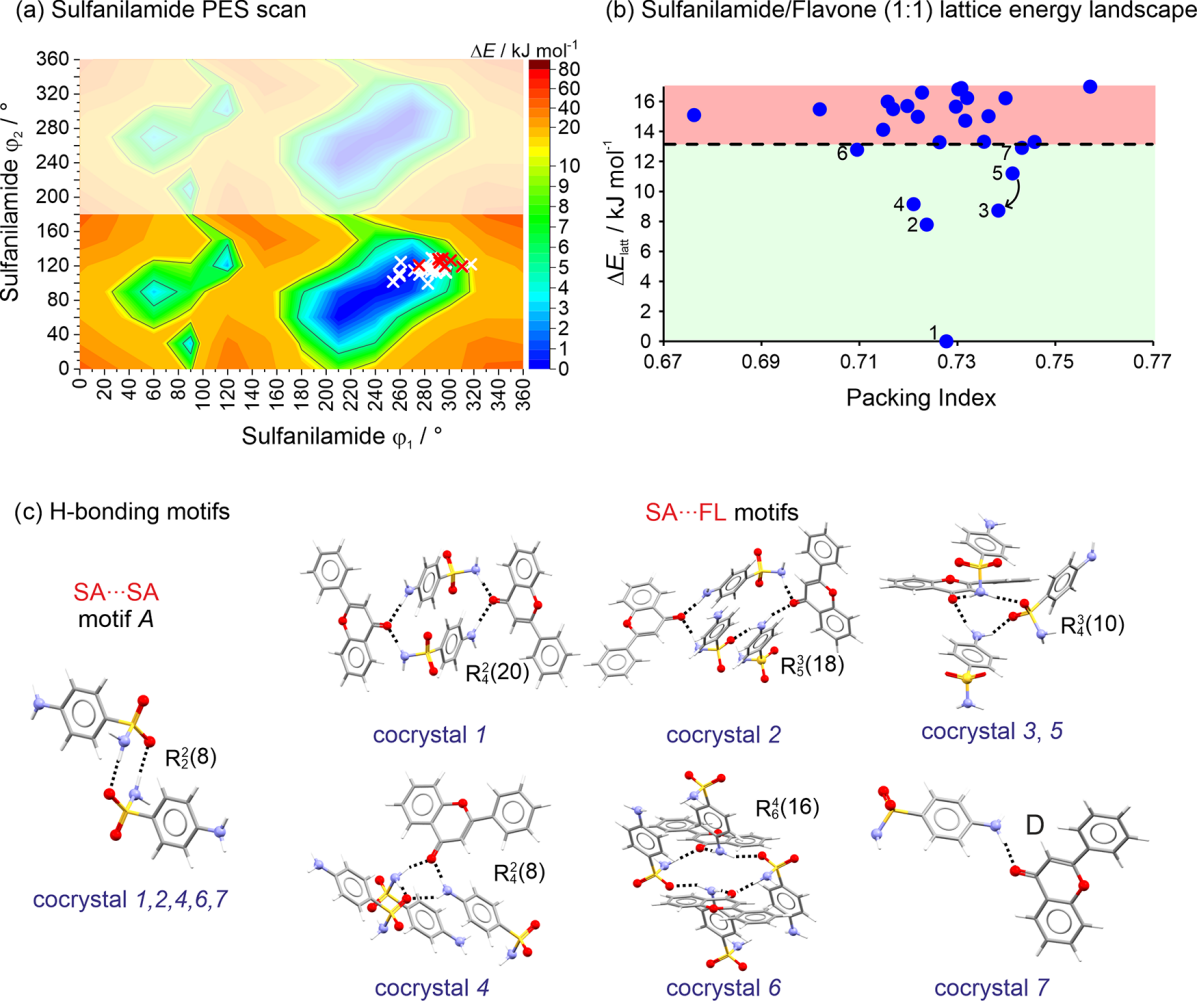
(a) Potential energy surface scan of SA. White crosses: conformations seen in hypothetical higher-energy structures; red crosses: SA conformations seen in lowest-energy cocrystals; regions that are symmetry-equivalent appear as blurred. (b) SA/FL (1:1) cocrystal lattice energy landscape. The cocrystal formation energy cutoff is indicated by the dashed line. Structures situated below this cutoff represent cocrystal structures that are energetically favorable over the individual single-component structures. (c) Key H-bonded motifs seen in the lowest-energy cocrystal structures.

The PES analysis of SA revealed several minima (Figure 5a), which can roughly be reduced to four minima regions: (1) dihedral angles ϕ_1_: 210–270°/ϕ_2_: 240–300°, (2) ϕ_1_: 210–270°/ϕ_2_: 60–120°, (3) ϕ_1_: 60–120°/ϕ_2_: 270–300°, and (4) ϕ_1_: 60–120°/ϕ_2_: 90–120°. These regions primarily differ in the pyramidalization of the −NH_2_ groups. Depending on the constraints, such as enforcing planar −NH_2_ groups, it becomes possible to generate a PES map featuring four equal low-energy regions, separated by energy barriers of approximately 15 kJ mol^–1^. Ignoring the pyramidalization of the aniline group, all generated cocrystal structures can be related to one low energy minimum and barely differ in ϕ_2_. Many conformations of SA in unstable predicted cocrystals fall within the 0–5 kJ mol^–1^ range of the PES scan, while those in stable cocrystals tend to be found between 4 and 9 kJ mol^–1^. This suggests that SA can adjust its conformation in favor of forming strong intermolecular interactions.

The lattice energies of the computationally generated cocrystals were then compared to those of the single-component structures. To estimate the cocrystal formation enthalpy, eq 1 was adapted by utilizing the respective *E*_latt_ values for *E*_CC_, *E*_API_, and *E*_FL_.

The SA/FL lattice energy landscape (Figure 5b) comprises seven structures, all of which exhibit a lattice energy lower than the sum of **α**_**SA**_ and **I**_**FL**_, and, thus, are potential cocrystals. Notably, one of these structures surpasses the stability of the individual components by 13 kJ mol^–1^ and is 7.8 kJ mol^–1^ more stable than the second most favorable predicted structure. This lowest-energy SA/FL structure adopts the *Pbca* space group, with all four H-bond donor groups of the SA molecule forming strong intermolecular interactions with neighboring SA and FL molecules. Furthermore, additional strong N–H···O, N–H···N, and π···π interactions are observed. For details, refer to section 11 of the SI.

A comparison of the thermodynamically feasible cocrystal structures reveals the presence of six distinct packing arrangements. Notably, structures 3 and 5 are very similar, and it is conceivable that structure 5 may transform into structure 3, making it imperceptible as a distinct packing arrangement. The majority of the structures form the strong dimer motif A (Figure 5c). This H-bonding motif dominates the energy landscape of the predicted cocrystals and only one of the lowest-energy packing arrangements (i.e., structures 3 and 5) does not feature this interaction. Conversely, in all six structurally distinct lowest-energy structures, unique H-bonding interactions exist between the SA and FL molecules. These include tetrameric [structure 1: *R*_4_^2^(20); 3,5: *R*_4_^3^(10); 4: *R*_4_^2^(8)], pentameric [2: *R*_5_^3^(18)], and hexameric [6: *R*_6_^4^(16)] ring motifs, as well as a D H-bonding motif observed in structure 7. In all of these structures FL forms two strong H-bonding interactions, each involving an aniline and a sulfonamide NH group, except for structure 7. Hence, the homomeric SA *R*_2_^2^(8) motif appears to be the favored building block in these cocrystals, which can also be found in 3 out of the 4 experimental structures of sulfanilamide. This motif allows a remarkable diversity of SO_2_NH_2_···FL interactions, ultimately leading to distinct computed packing arrangements.

#### 3.3.3 Sulfaguanidine/Flavone PES and Lattice Energy Landscape

**Figure 6:**
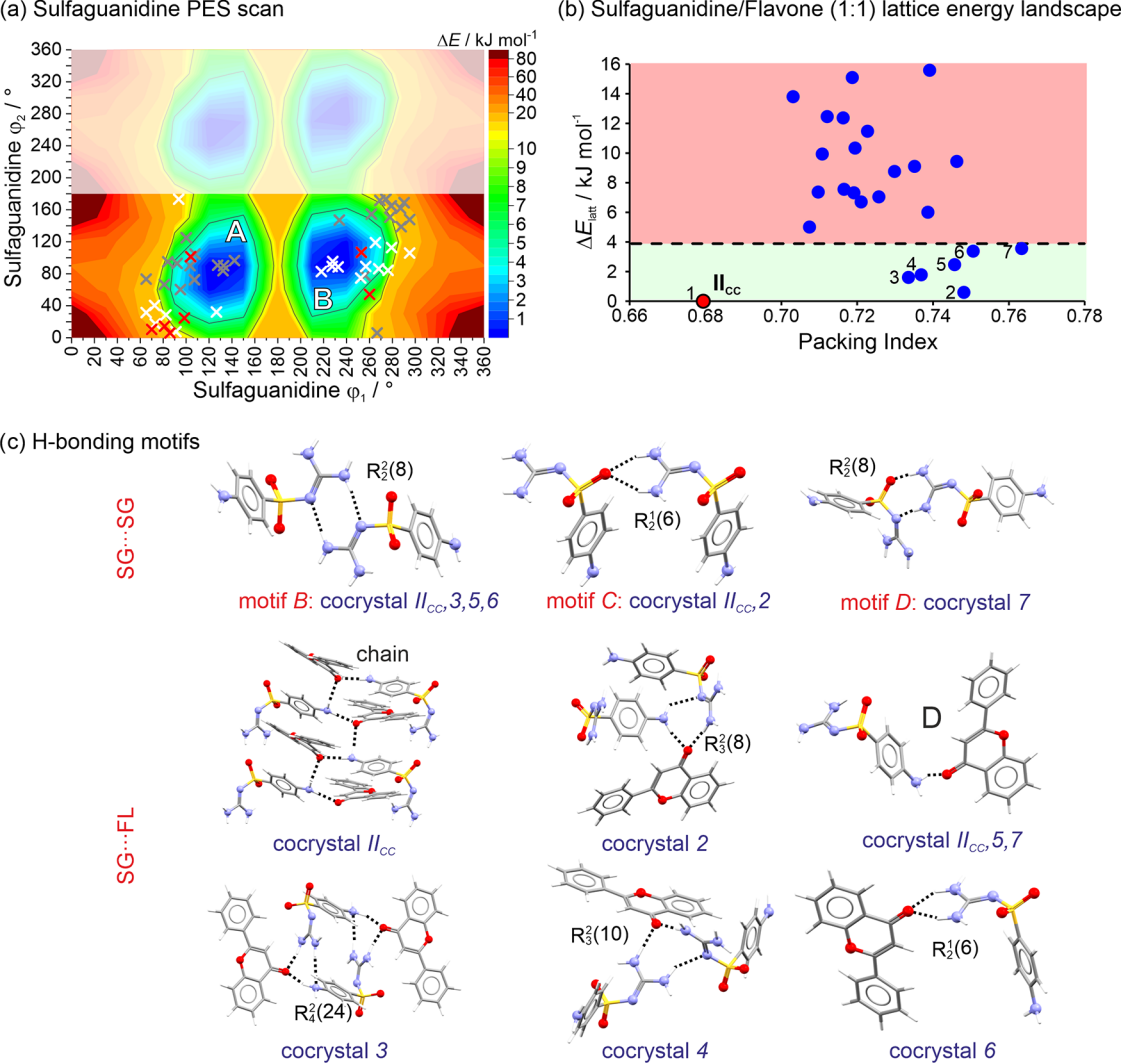
(a) Potential energy surface scan of SG. White crosses: conformations seen in hypothetical higher-energy structures; red crosses: SG conformations seen in lowest-energy cocrystals; gray crosses: alternative conformations seen in centrosymmetric space groups. Regions that are symmetry-equivalent appear as blurred. (b) SG/FL (1:1) cocrystal lattice energy landscape. The cocrystal formation energy cutoff is indicated by the dashed line. Structures situated below this cutoff represent cocrystal structures that are energetically favorable. **II**_**CC**_, a *Z*′ = 2 cocrystal has been added to the figure. (c) Key H-bonded motifs seen in the lowest-energy cocrystal structures.

For SG (planar −NH_2_ groups) two minima were observed (Figure 6a). This means that the aniline group remains conformationally identical when exceeding 180°, and the rotation of the guanidine group results in an inversion symmetry relation, creating regions A and B. In the cocrystal searches, two conformations were utilized: the global minimum and a higher-energy conformation, in alignment with our previous SG study.^53^

The SG/FL crystal energy landscape encompasses the predicted *Z*′ = 1 structures and **II**_**CC**_, a *Z*′ = 2 structure that was beyond the scope of the search. As for **I**_**CC**_, its structure remains elusive, but there are indications that **I**_**CC**_ is a *Z*′ > 1 structure (from indexing and IR spectroscopy). The calculations suggest that seven of the structures depicted in Figure 6b represent feasible cocrystal packings, with **II**_**CC**_ emerging as the most stable structure. The stabilization energy of **II**_**CC**_ is notably lower than that of the hypothetical SA/FL cocrystal structures, specifically −3.8 kJ mol^–1^ compared to −13.1 kJ mol^–1^. One intriguing aspect of Figure 6b is that the majority of the structures deviate significantly from the gas-phase minimum conformation (Figure 6a). Nevertheless, none of these conformations deviate by more than 18 kJ mol^–1^ in intramolecular energy. This suggests that unfavorable conformations are offset by the formation of strong intermolecular interactions, such as H-bonding and aromatic interactions.

Among the seven thermodynamically feasible cocrystal structures, each is unique. Six out of these seven structures include at least one of the three SG···SG motifs depicted in Figure 6c. Two of these motifs (B and D) can also be found in the polymorphs of SG. Among the seven cocrystal structures, six distinct SG···FL H-bonding interaction motifs are observed, encompassing a D-motif, a chain motif (**II**_**CC**_), and four distinct ring motifs: a dimeric [structure 6: *R*_2_^1^(6)], two trimeric [2: *R*_3_^2^(8); 4: *R*_3_^2^(10)], and a tetrameric [3: *R*_4_^2^(24)]. In contrast to the SA/FL cocrystal structures, the additional H-bonding capabilities of the SG molecule promote the formation of a wider range of distinct SG···SG H-bonding interactions, alongside the diversity of SG···FL interactions, as also observed in SA/FL.

#### 3.3.4 Dapsone/Flavone PES and Lattice Energy Landscape

**Figure 7:**
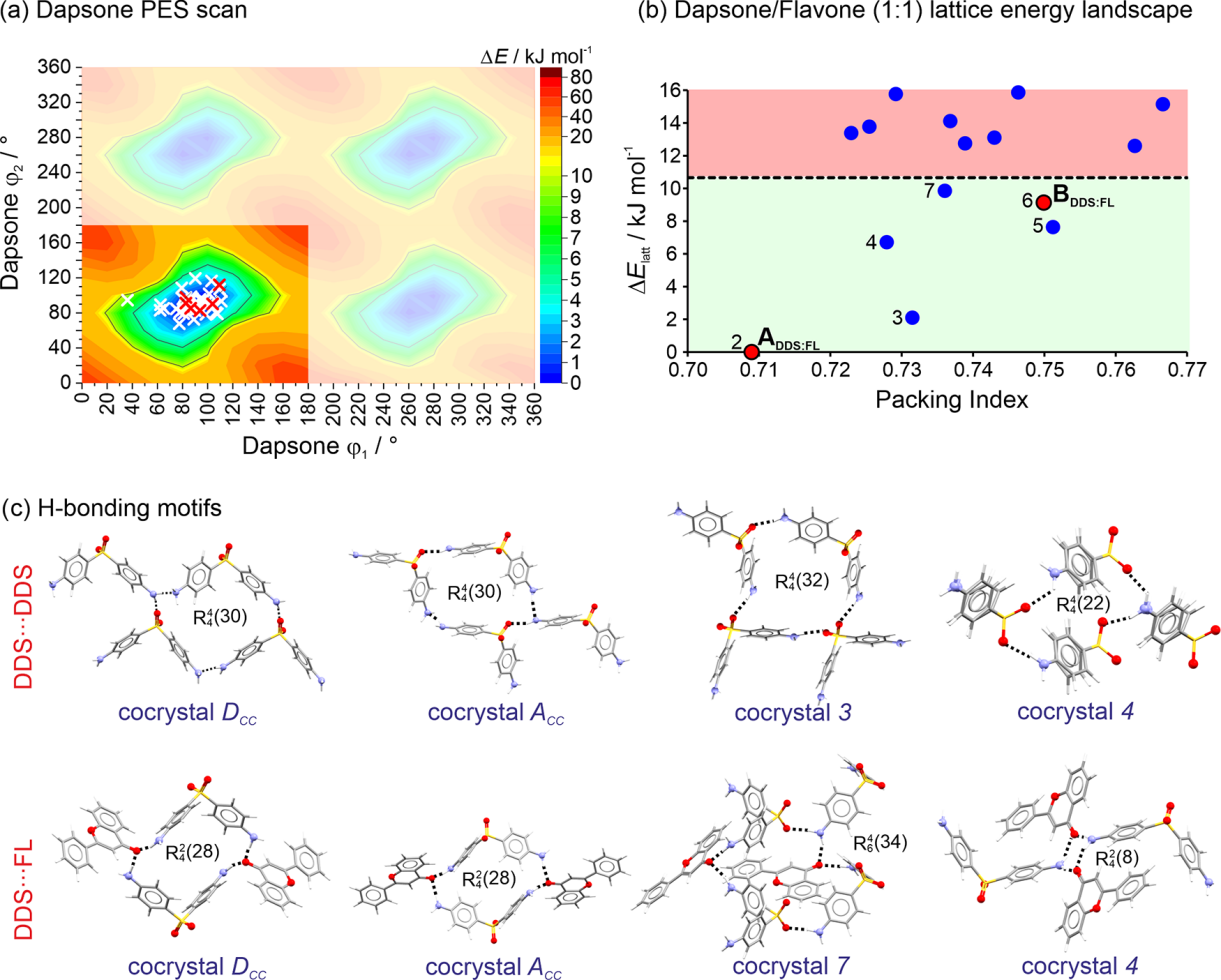
(a) Potential energy surface scan of DDS. White crosses: conformations seen in hypothetical higher-energy structures; red crosses: DDS conformations seen in lowest-energy cocrystals; regions that are symmetry-equivalent appear as blurred. (b) DDS/FL (1:1) cocrystal lattice energy landscape. The cocrystal formation energy cutoff is indicated by the dashed line. Structures situated below this cutoff represent cocrystal structures that are energetically favorable. (c) Key H-bonded motifs seen in the lowest-energy cocrystal structures.

The PES scan for DDS revealed four minima, which are identical when the −NH_2_ group is modeled as planar (Figure 7a). It has been previously reported that the values of ϕ_1_ and ϕ_2_ can vary considerably with only a minimal energy penalty (Δ*E*_intra_).^62^ In the DDS/FL (1:1) crystal energy landscape, there are seven structures within 10 kJ mol^–1^ of the global minimum structure (section 6 of the SI). These structures are all potential cocrystals based on lattice energy estimations. Notably, among these structures are the two (1:1) polymorphs of DDS/FL, **A**_**CC**_ and **B**_**CC**_ (Figure 7b). The other three DDS/FL cocrystals lie outside specified search criteria.

The intramolecular energy penalty for the observed DDS conformations in the computed cocrystal structures generally lies within 5 kJ mol^–1^ of the gas-phase minimum (Figure 7a). However, the ϕ_1_ and ϕ_2_ angles deviate by approximately ±30° across these conformations.

All lowest-energy (1:1) cocrystal structures, **D**_**CC**_, and the anhydrates of DDS form C(8) chain motifs involving the amine and sulfone groups. Furthermore, C(12) chain motifs are formed in **D**_**CC**_ and **A**_**CC**_. In four of the cocrystal structures (**D**_**CC**_, **A**_**CC**_, 2, and 3) the combination of chain motifs translates into characteristic ring motifs (Figure 7c). Four DDS molecules form an *R*_4_^4^(30) motif in **D**_**CC**_ and **A**_**CC**_, an *R*_4_^4^(32) motif in structure 2, and an *R*_4_^4^(22) motif in structure 3. The flavone molecules of the cocrystals are all bound through O···H–N to DDS, involving the carbonyl O atom. In four of the lowest-energy structures heteromolecular ring motifs are formed. Two DDS and two FL molecules form an *R*_4_^2^(28) motif in **D**_**CC**_ and **A**_**CC**_ and an *R*_4_^2^(8) motif in structure 3. A hexameric *R*_6_^4^(34) ring motif, involving four DDS and two FL molecules, is present in structure 6.

Overall, the DDS molecules show preferential intermolecular interactions, i.e., a chain motif; however, the packing diversity of DDS chains is significantly increased through the addition of the flavone molecule compared to the DDS anhydrate crystal energy landscape.^27^ Furthermore, the calculations suggest a strong driving force for DDS/FL cocrystal formation.

### 3.4 Flavone Polymorphism

#### 3.4.1 Thermal Analysis, IR Spectroscopy, and PXRD

PXRD characterization of the starting material revealed that it did not correspond to the phase described by Waller et al.^59^ Instead, it represents a polymorphic form, **II**_**FL**_, which exhibits a melting point at 96.5 ± 0.1 °C along with a heat of fusion of 21.1 ± 0.2 kJ mol^–1^. Upon cooling the melt, another polymorphic form, **III**_**FL**_, recrystallizes at approximately 78 °C. Form **III**_**FL**_ often recrystallizes concomitantly with **II**_**FL**_. The melting point of **III**_**FL**_ was determined at 95.6 ± 0.2 °C, with a heat of fusion of 19.6 ± 0.2 kJ mol^–1^. Thus, based on the heat of fusion rule,^89,90^ the polymorphs **II**_**FL**_ and **III**_**FL**_ are monotropically related. Hot-stage microscopic investigations showed the transition from **III**_**FL**_ to **II**_**FL**_ (section 8.4 of the SI).

The IR spectra and PXRD diffractograms of **II**_**FL**_ and **III**_**FL**_ polymorphs exhibit a high degree of similarity, whereas **I**_**FL**_ can be easily distinguished from the two polymorphs (section 8 of the SI). The crystal structure of **I**_**FL**_ has already been published^59^ and is being discussed alongside **II**_**FL**_, which was determined from PXRD data.

#### 3.4.2 Crystal Structures

**Figure 8:**
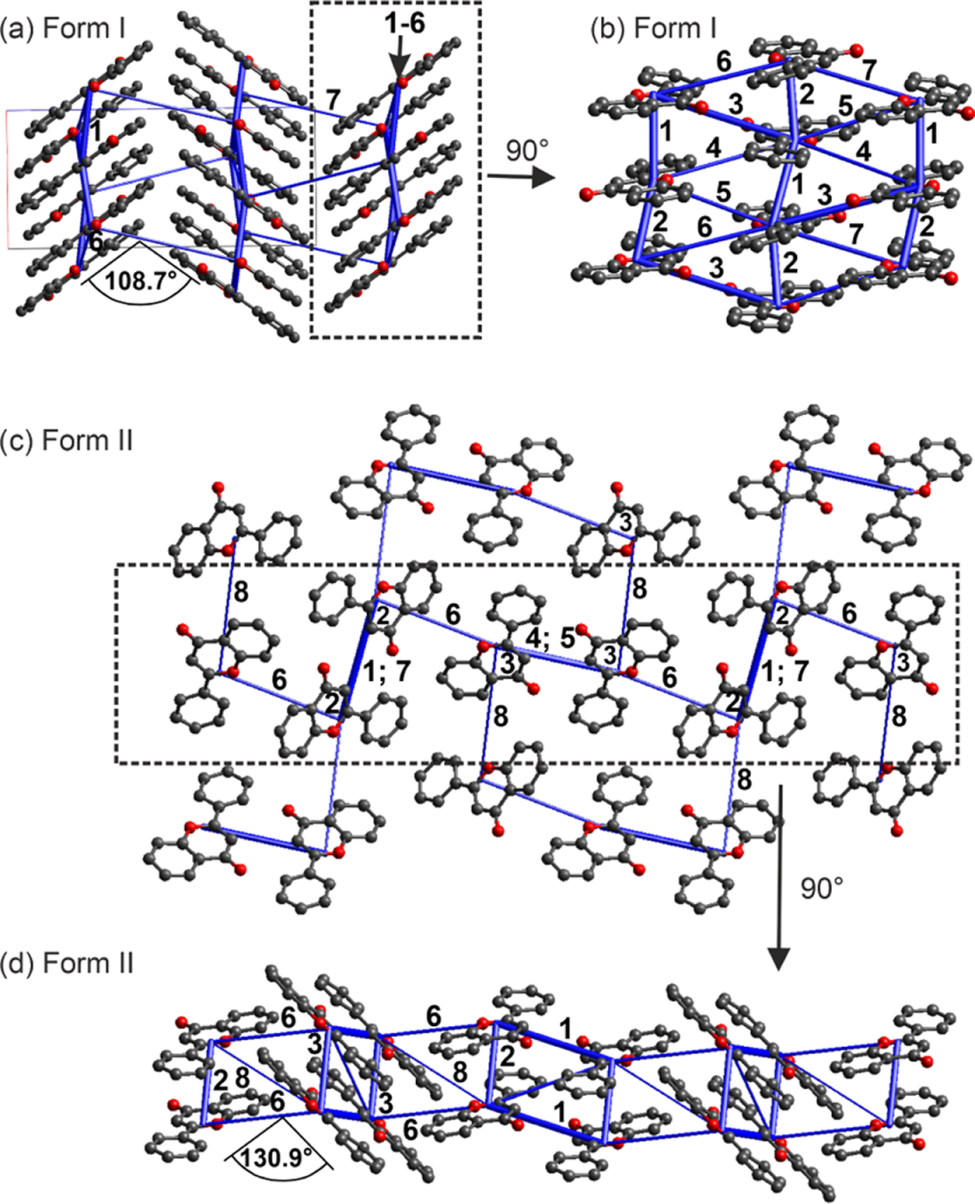
Energy framework diagrams (total energy) for (a, b) **I**_**FL**_ and (c, d) **II**_**FL**_. The energy scale factor is 50. Stabilizing contacts are shown in blue, and the thickness corresponds to the strength. Pairwise interaction energies <10 kJ mol^–1^, and H atoms are omitted for clarity.

**I**_**FL**_ crystallizes in the orthorhombic space group *P*2_1_2_1_2_1_ with two molecules in the asymmetric unit. The conformations deviate by less than 0.5 kJ mol^–1^ in energy from the global minimum conformation. As shown in Figure 8a, the FL molecules are arranged in a herringbone motif, forming an angle of 108.7° with respect to each other. The symmetry-independent molecules are stacked alternatively along the crystallographic *a* axis, forming strong aromatic interactions, which account for −42.9 and −41.3 kJ mol^–1^ in pairwise energy (Figure 8b, labeled as 1 + 2; section 11 of the SI). Furthermore, C–H···O as well as C–H···π interactions (Figure 8b, labeled as 3–7) are formed.

The second polymorph, **II**_**FL**_, crystallizes in the monoclinic space group *P*2_1_/*n*, with *Z*′ = 2. Similar to **I**_**FL**_, the conformation of FL with torsion angles of 169.6 and 175.7° deviates by less than 0.5 kJ mol^–1^ in intramolecular energy from the global minimum conformation. A herringbone-like arrangement can be observed for the flavone molecules, with each of the two symmetry-independent molecules forming a distinct herringbone motif, one along the crystallographic *a* axis and the other along the crystallographic *c* axis. The strongest interaction is a C–H···O close contact (−42.6 kJ mol^–1^ in pairwise energy, contact 1 in Figure 8c,d). For more details, refer to section 11 of the SI.

**III**_**FL**_ could be indexed to a potential monoclinic cell (*P*2_1_, *Z*′ = 4), and based on the lattice parameters, appears to be related to **II**_**FL**_. However, we were not able to solve the structure. Disorder may be present.

### 3.5 Sulfaguanidine/Flavone Cocrystal System

The experimental cocrystal screening yielded two SG/FL cocrystals, both displaying a 1:1 molar ratio of the two compounds, effectively establishing them as cocrystal polymorphs.

#### 3.5.1 PXRD and IR Spectroscopy

**Figure 9:**
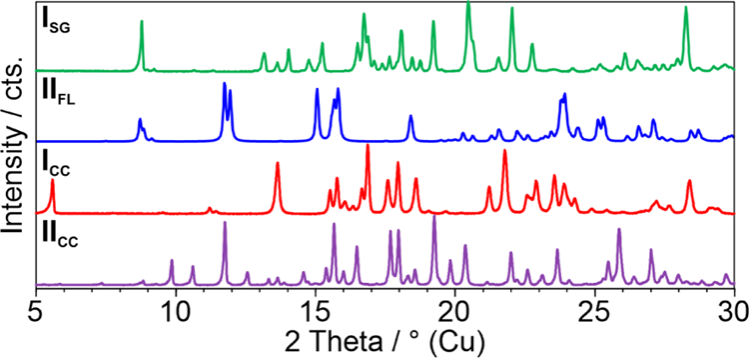
PXRD pattern of SG form I (**I**_**SG**_), flavone (**II**_**FL**_), sulfaguanidine/flavone form I (**I**_**CC**_), and sulfaguanidine/flavone form II (**II**_**CC**_).

The PXRD patterns (Figure 9) and IR spectra differ significantly from those of the parent compounds. To offer a more concise representation, only the reactants and the resulting cocrystals are presented. The cocrystal diffraction patterns are distinct from all solid-state forms of the starting materials (section 9 of the SI).

The IR spectra (section 9 of the SI) reveal significant shifts in the υ-NH bands, specifically at 3335 cm^–1^ in **I**_**CC**_ and 3341 cm^–1^ in **II**_**CC**_. Both cocrystals exhibit a shift in the symmetric SO_2_ vibration, from 1120 cm^–1^ in **I**_**SG**_ to 1126 cm^–1^ in **I**_**CC**_ and 1129 cm^–1^ in **II**_**CC**_. Additionally, the υ(C=O) vibration of FL at 1640 cm^–1^ shifts to 1635 cm^–1^ in both cocrystals. Furthermore, both cocrystal forms display bands between 3420 and 3440 cm^–1^, indicating the presence of H-bonding interactions.

#### 3.5.2 Thermal Analysis and Thermodynamic Stability

**Figure 10:**
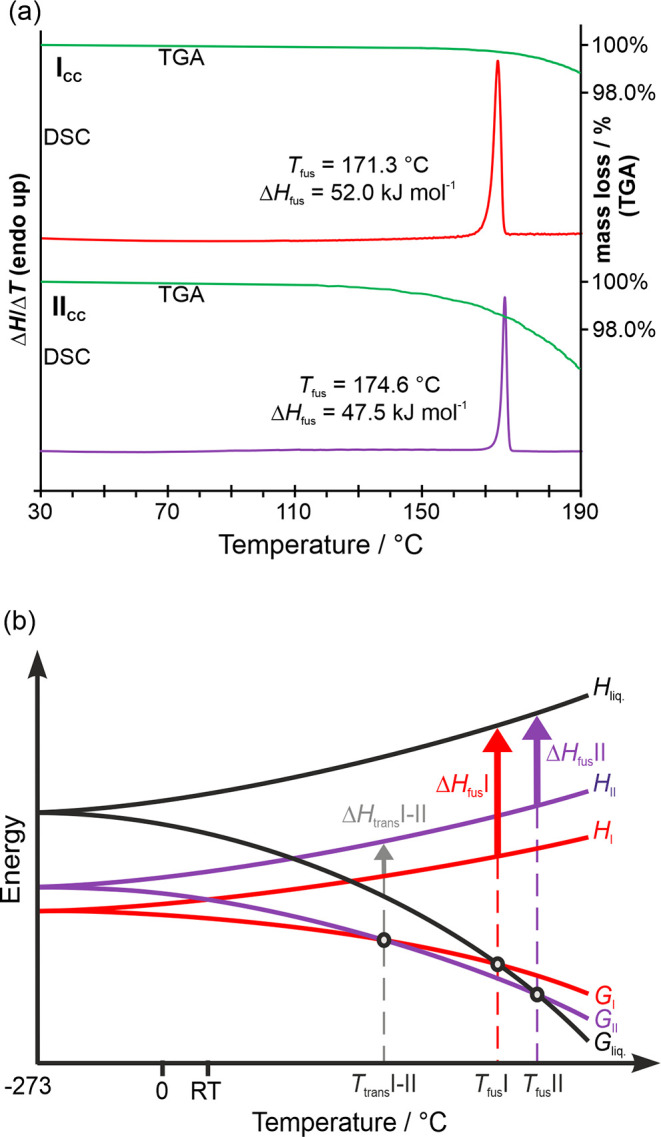
Thermal analysis of SG/FL cocrystal polymorphs. (a) DSC (red—**I**_**CC**_, purple—**II**_**CC**_) and TGA (green) thermograms, recorded with a heating rate of 10 °C min^–1^. (b) Semischematic energy/temperature diagram of **I**_**CC**_ and **II**_**CC**_; *T*_fus_—melting point, *T*_trans_—transition point, *H*_fus_—enthalpy of fusion, *H*_trans_—enthalpy of transition, *H*_liq._—enthalpy of the melt, *G*—Gibbs free energy.

Thermal analysis techniques revealed that the **I**_**CC**_ and **II**_**CC**_ cocrystals are anhydrous forms. When subjected to heating from 25 to 205 °C at a rate of 10 °C min^–1^, they exhibit minimal mass loss, amounting to less than 2% (TGA). This mass loss can be attributed to the sublimation of FL from the cocrystals (Figure 10a, green). The DSC diagram of **I**_**CC**_ shows the melting of the cocrystal at 171.3 ± 0.1 °C, with a heat of fusion of 52.0 ± 0.3 kJ mol^–1^. Upon cooling and/or reheating, either **I**_**CC**_ or a mixture of **I**_**CC**_ and **II**_**CC**_ crystallizes from the melt. This aligns with the findings from the hot-melt extrusion experiments, where **I**_**CC**_ was primarily obtained at lower temperatures, specifically ≤145 °C, whereas **II**_**CC**_ was formed at higher temperatures, generally ≥165 °C. The phase pure **II**_**CC**_ melts at 174.6 ± 0.1 °C with an energy of fusion of 47.5 ± 0.1 kJ mol^–1^. Therefore, following the heat of fusion rule,^89,90^ the cocrystal polymorphs are enantiotropically related, with **II**_**CC**_ being the high-temperature form. The heat of transition enthalpy of **I**_**CC**_ to **II**_**CC**_ was calculated as 4.5 ± 0.4 kJ mol^–1^. Based on these results, a semischematic temperature/energy diagram was constructed (Figure 10b), and the transition temperature could be estimated^91,92^ as 142 °C.

#### 3.5.3 **II_CC_** Crystal Structure

**Figure 11:**
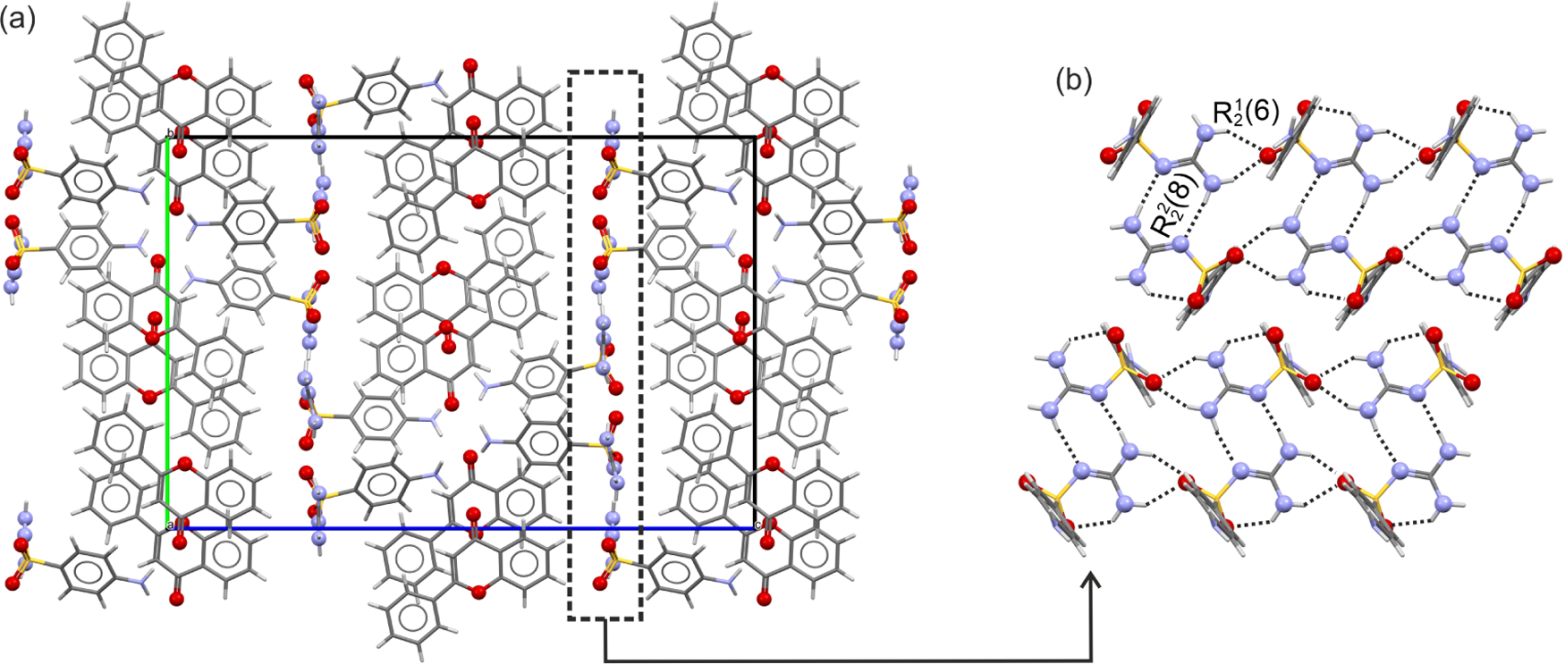
SG/FL II_CC_ crystal structure. (a) Packing diagram viewed along the crystallographic *a* axis and (b) strong H-bonding interactions seen in the layers formed by the guanidinium and SO_2_ functions.

**II**_**CC**_ crystallizes in the space group *P*2_1_2_1_2_1_ with two SG and two FL molecules in the asymmetric unit. Each of the two symmetry-equivalent SG molecules is stacked in the direction of the crystallographic *a* axis, forming *R*_2_^1^(6) motifs between the SO_2_ and guanidinium functions (Figure 11b), which account for −62.5 and −59.6 kJ mol^–1^ in pairwise energy (section 11 of the SI). The guanidine and sulfonyl groups of SG are arranged in a plane parallel to (001), with the strongest interaction being an *R*_2_^2^(8) motif (−105.9 kJ mol^–1^, Figure 11b), formed by the two crystallographically independent molecules (2× N–H···N). The *p*-aminophenyl groups stick out of the planes (Figure 11), and the FL molecule interacts with them through N–H···O and π···π interactions. Furthermore, the SG molecules form strong C–H···π close contacts. Adjacent layers of the SG planes are related by 2_1_ symmetry.

### 3.6 Dapsone/Flavone Cocrystal System

We were able to reproduce and characterize the four literature cocrystals^22,23^ alongside a fifth novel cocrystal, **E**_**CC**_. The PXRD patterns differ significantly from those of the starting materials and notable distinctions exist among the cocrystals. Moreover, the IR spectra of the five cocrystals exhibit notable differences in their ν(N–H), ν(S=O), and ν(C=C) bands. For more details, see section 10 of the SI.

#### 3.6.1 Thermal Analysis and Lattice Energy Calculations

**Figure 12:**
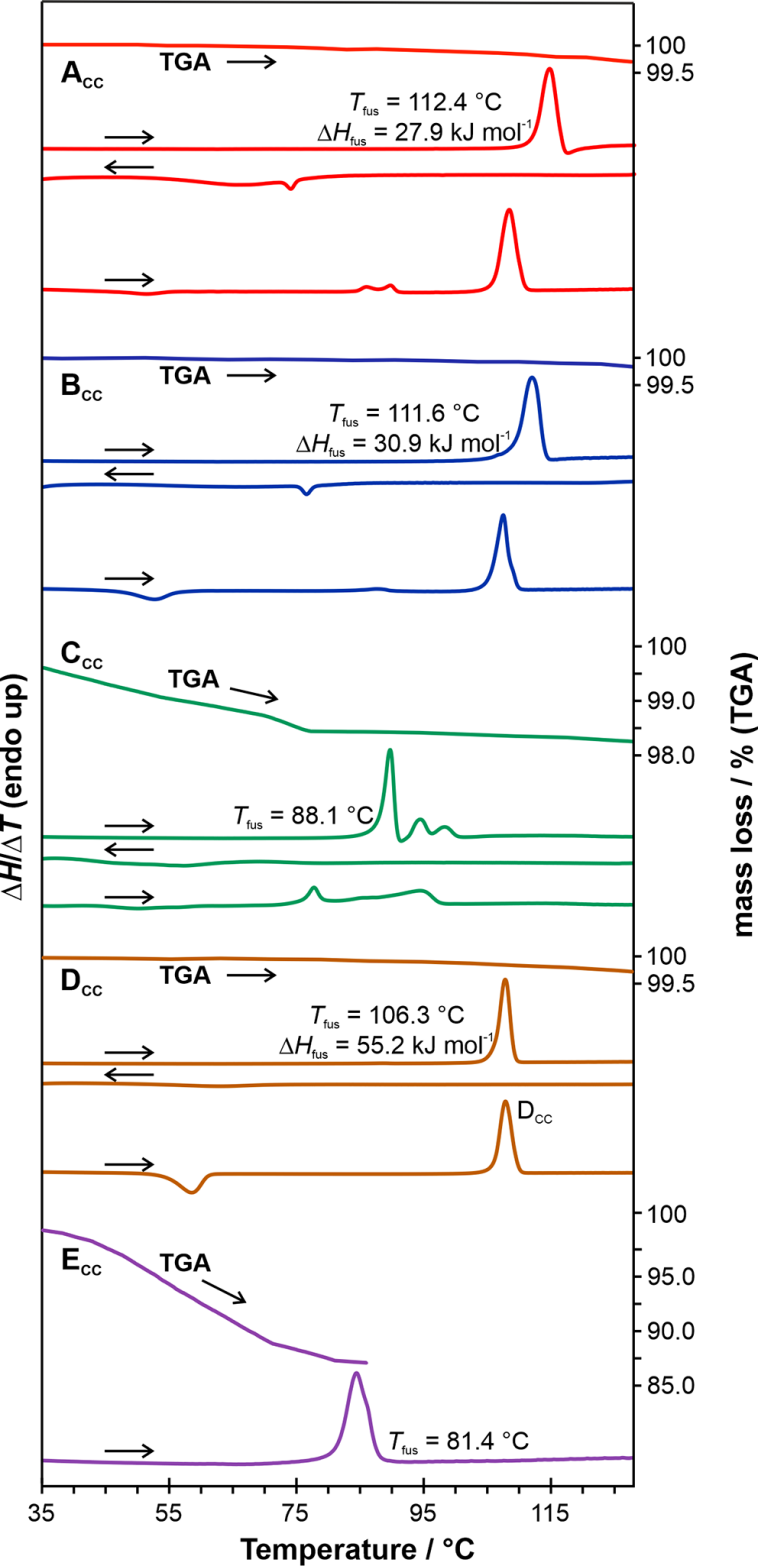
DSC and TGA thermograms of the five DDS/FL cocrystals: **A**_**CC**_, **B**_**CC**_, **C**_**CC**_, **D**_**CC**_, and **E**_**CC**_.

**A**_**CC**_ melts at 112.4 ± 0.1 °C with a heat of 27.3 ± 0.4 kJ mol^–1^ (Figure 12, red curves). When **A**_**CC**_ is heated above its melting temperature, **II**_**DDS**_ recrystallizes, and, upon cooling, **D**_**CC**_ crystallizes. The cooling curve shows a sharp exotherm related to the transformation of **II**_**DDS**_ to **III**_**DDS**_ (red curve), and the second, broad exotherm corresponds to the recrystallization of **D**_**CC**_, which is also observed upon reheating the sample. The first endotherm upon reheating corresponds to the **III**_**DDS**_ to **II**_**DDS**_ transformation,^28^ and the second endotherm represents the eutectic temperature between **D**_**CC**_ and **II**_**DDS**_ (86–87 °C), followed by the dissolution of **D**_**CC**_ in the melt. The mass loss between 20 and 130 °C with a heating rate of 10 °C min^–1^ is less than 0.2%, indicating the presence of an anhydrate.

**B**_**CC**_ melts at 109.5 ± 0.1 °C with a heat of 30.1 ± 0.4 kJ mol^–1^ (Figure 12, blue curves). **B**_**CC**_ displays similar behavior to **A**_**CC**_, with **II**_**DDS**_ recrystallizing at the melting temperature of **B**_**CC**_. Thermogravimetric analysis showed a mass loss of about 0.1%, confirming that this cocrystal is an anhydrate. Based on the heat of fusion rule it can be concluded that **A**_**CC**_ and **B**_**CC**_ are enantiotropically^89,90^ related (enthalpies were used despite the fact that recrystallization of **II**_**DDS**_ occurred, but in same quantities), with **A**_**CC**_ being the high-temperature form and **B**_**CC**_ the RT and low-temperature form. This observation is in contrast to the literature, where **A**_**CC**_ and **B**_**CC**_ were described as a monotropic system,^23^ but agrees with the fact that **B**_**CC**_ is obtained in slurry experiments at RT, and **A**_**CC**_ shows a higher melting point than **B**_**CC**_. The relative heat of fusion difference reported in the literature is in line with our measurements; however, the melting point temperatures of **A**_**CC**_ and **B**_**CC**_ were reported reversed^23^ to what we observed.

**C**_**CC**_ exhibits an inhomogeneous melting behavior and decomposes into **D**_**CC**_ and **II**_**DDS**_. TGA reveals a mass loss of 1.8%, which corresponds to approximately 0.5 mol of water per mole of cocrystal (Figure 12, green curves), as additionally confirmed by Karl Fischer titration. Subsequent moisture (de)sorption analysis unveiled that **C**_**CC**_ is not a neat cocrystal, as previously described in the literature,^23^ but a nonstoichiometric cocrystal hydrate with 0–0.67 mol of water per mole (1:1) cocrystal (section 10 of the SI).

**D**_**CC**_ melts at 106.2 ± 0.1 °C with a heat of fusion of 55.2 ± 0.1 kJ mol^–1^. No recrystallization of DDS is observed at the melting temperature of **D**_**CC**_. Upon reheating the cooled sample, **D**_**CC**_ recrystallizes. TGA indicates a mass loss of 0.2%, confirming that it is an anhydrate (Figure 12, orange curves).

The cocrystal solvate, **E**_**CC**_, melts at approximately 81 °C. Thermogravimetric analysis reveals a mass loss of 13.1%, equivalent to 1.0 mol of *t*-butanol per mole of 1:1 cocrystal. Therefore, **E**_**CC**_ is a monosolvate. Storage of the cocrystal solvate at various relative humidities (0, 50, and 100%) led to its decomposition into cocrystal **A**_**CC**_, **D**_**CC**_, and **III**_**DDS**_.

Lattice energy calculations (DFT-*d*) were employed to assess the stability order of three DDS/FL cocrystals, using different dispersion corrections alongside two sets of pseudopotentials (CASTEP standard and C19 OTF ultrasoft pseudopotentials). To enable a comparison of the three cocrystals’ stability, the lattice energy of FL was subtracted from **D**_**CC**_. Among the six methods employed (Table 3), five consistently identified **D**_**CC**_ as the structure with the lowest energy (stoichiometry correction applied). Depending on the dispersion correction used, either **A**_**CC**_ or **B**_**CC**_ emerged as the second most stable cocrystal. Combining both, experimental and computational data, the thermodynamic stability at 0 K is as follows: **D**_**CC**_ (most stable) > **B**_**CC**_ > **A**_**CC**_ (least stable). Therefore, the calculated stability order of cocrystals **A**_**CC**_ and **B**_**CC**_ in Figure 7b is incorrect. This discrepancy is accurately described in qualitative terms by the PBE-TS and PBE-D2 methods. Further investigation is required to precisely calculate the energy difference; however, it is noteworthy that both structures were predicted to be stable.

**Table 3:** Lattice Energy Differences between DDS/FL Cocrystals

	Δ*E*_latt_ (relative to D_CC_)/kJ mol^–1^					
cocrystal	PBE-TS^93^	PBE-MBD*^73^	PBE-MBD*^73^^a^	PBE-D2^94^	PBE-D3-BJ^95^	PBE-D4^96^
**A**_**CC**_	13.52	–2.66	1.07	3.18	5.41	4.67
**B**_**CC**_	11.95	6.47	2.92	2.97	8.23	11.28
**D**_**CC**_	0	0	0	0	0	0

a C19 OTF ultrasoft pseudopotentials were used.

#### 3.6.2 E_CC_ Crystal Structure

The crystal structures of cocrystals **A**_**CC**_, **B**_**CC**_, and **D**_**CC**_ have already been published.^23^ Cocrystal **E**_**CC**_ is described below, while **C**_**CC**_ still remains elusive. As already described, **A**_**CC**_, **B**_**CC**_, and **D**_**CC**_ share common H-bonding motifs.

**Figure 13:**
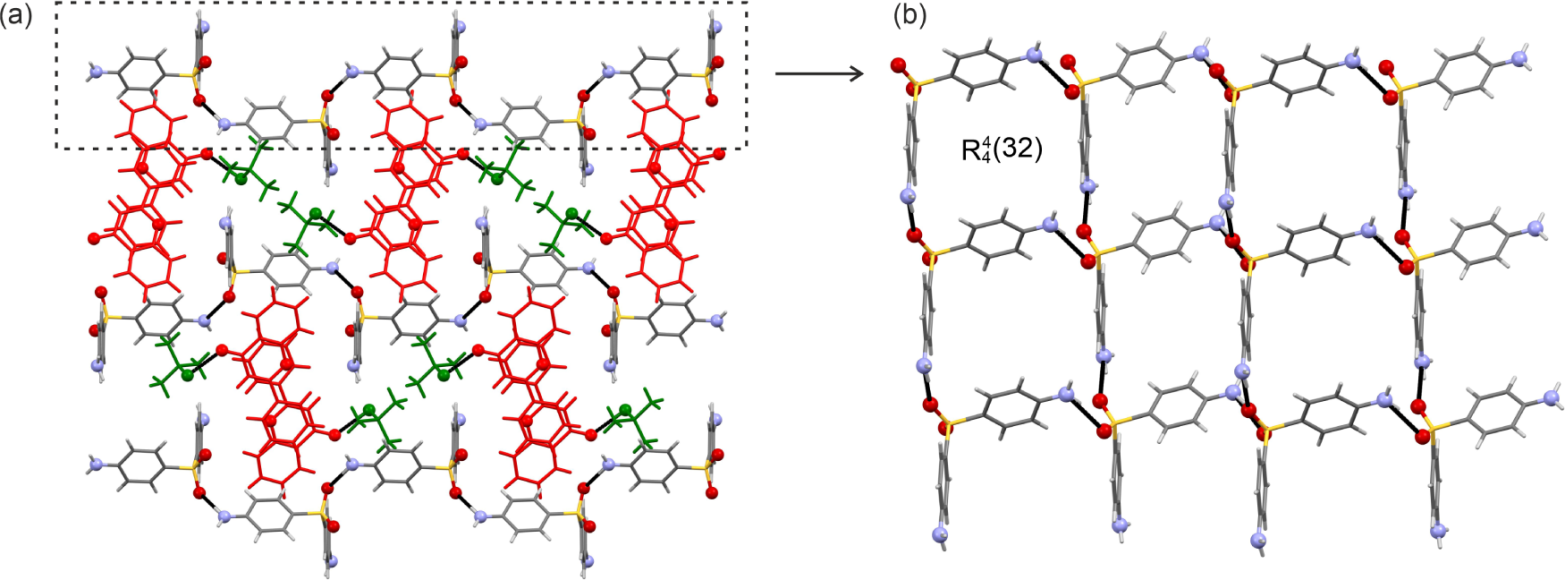
**E**_**CC**_ crystal structure: (a) packing diagram viewed along the crystallographic *a* axis with DDS color-coded by element (FL in red and *t*-BuOH in green). (b) H-bonded layer network of DDS.

**E**_**CC**_ crystallizes in the monoclinic *P*2_1_/*c* space group, with one molecule of DDS, FL, and *t*-butanol each in its asymmetric unit. Each of the four DDS H-bond donor groups forms strong intermolecular interactions: two N–H···O_DDS_, one N–H···O_FL_, and one N–H···O_*t*-BuOH_. Consequently, all three distinct entities are interconnected through H-bonding. The O atoms of the SO_2_ group serve as acceptors for the two N–H···O_DDS_ interactions, resulting in tetrameric *R*_4_^4^(32) ring motifs and interconnected layers of DDS molecules (Figure 13). These DDS layers align parallel to the (010) plane, and the strong H-bonding interactions account for −33.2 and −33.6 kJ mol^–1^ in pairwise interaction energies. The FL···DDS H-bonding interaction contributes −20.7 kJ mol^–1^, while the *t*-BuOH···DDS contributes −32.0 kJ mol^–1^ in pairwise energy. In addition to these classical and strong H-bonding interactions, aromatic interactions play a significant role in stabilizing the **E**_**CC**_ lattice. In **E**_**CC**_, π···π interactions of FL molecules are the strongest contributors (−48.4 kJ mol^–1^ in pairwise energy).

### 3.7 Comparison of API/Flavone Cocrystals

A comparison of the nature and strength of pairwise intermolecular interactions within the API, coformer, and cocrystal structures was performed, using CrystalExplorer.^76^ The pairwise intermolecular interactions, all within a 3.8 Å radius around the central molecule, were calculated. In cases of higher *Z*′ structures, average interaction energies were used. Subsequently, the energies of individual components, specifically the strongest interaction and the sum of all interactions, were contrasted (Table 4).

**Table 4:** Pairwise Intermolecular Interaction Energy Comparisons of API, FL, and Cocrystal Structures^a^

solid form	most stable interaction	∑API interaction/kJ mol^–1^	∑FL interaction/kJ mol^–1^	strongest pairwise API interaction/kJ mol^–1^	strongest pairwise FL interaction/kJ mol^–1^
**II**_**FL**_	C–H···O		–114.3		–42.6
**III**_**DDS**_	N–H···O	–172.8		–43.1	
**V**_**DDS**_	N–H···O	–175.6		–50.2	
**I**_**SG**_	2× N–H···N	–244.9		–117.4	
**II**_**SG**_	2× N–H···N	–200.4		–91.8	
**β**_**SA**_	N–H···O	–160.9		–47.2	
**A**_**CC**_	API···FL (aromatic)	–157.5	–124.4	–34.7	–34.7
**B**_**CC**_	API···FL (aromatic)	–169.0	–122.8	–37.8	–37.8
**D**_**CC**_	FL···FL (C–H···O)	–150.2	–119.2	–32.0	–39.0
**II**_**CC**_	SG···SG (2× N–H···N)	–199.9	–114.8	–105.9	–37.6
SA/FL (1)	SA···SA (2× N–H···O)	–146.7	–134.7	–77.9	–42.0

a Note that, in contrast to the *E*_latt_ calculations, intramolecular energy penalties are disregarded.

Starting with the single-component crystals, there is a notable prevalence for strong π···π and C–H···O interactions in the coformer (FL), accompanied by comparatively weaker C–H···π interactions. In the DDS polymorphs, strong N–H···N, N–H···O, π···π, N–H···π, and C–H···π interactions are observed. On the other hand, the SA polymorphs rely significantly on H-bonds for their stability. Across all four polymorphs, N–H···O interactions emerge as the strongest, closely followed by C–H···π and C–H···N interactions. SG stands out due to its exceptional strength of the H-bonding interactions, particularly involving the guanidine group and the sulfone oxygen atoms.

Notably among the DDS/FL cocrystals, the strongest interactions observed in **A**_**CC**_ and **B**_**CC**_ involve aromatic interactions (DDS···FL), contrary to the anticipated H-bonding interactions. Additionally, a comparison of the sum of all DDS interactions in both single- and multicomponent crystals revealed that DDS can form more stable interactions in the single-component crystals than in the cocrystal. Conversely, FL exhibits the opposite behavior, with the sum of FL interactions being stronger in the DDS cocrystal structures. This difference can be attributed to FL’s ability to form H-bonding interactions only in the cocrystals. The DDS···FL interactions contribute more to the lattice energy in **A**_**CC**_ and **D**_**CC**_ than the DDS···DDS interactions. However, the sums of DDS···FL and DDS···DDS interactions in **B**_**CC**_ are of similar strengths. When considering only the strongest intermolecular interaction observed in DDS, FL, and DDS/FL, it was found that the strength of intermolecular interactions is higher in the single-component forms than in the cocrystals, a surprising outcome, especially for FL. Overall, these findings highlight the importance and strength of aromatic interactions and the importance of the interplay of all potential interactions. The fact that FL can form stronger interactions in the cocrystals overall (sum of interactions considered) may be the driving force behind cocrystallization of DDS with FL. The inclusion of water or *t*-BuOH might even offer better interaction possibilities, as evidenced by the strongest pairwise intermolecular interaction found in the cocrystal solvate **E**_**CC**_.

In the case of the SG/FL **II**_**CC**_, where SG interactions are weaker than those observed in the SG polymorphs, the surprising finding is that FL interactions exhibit comparable strength in both single- and multicomponent crystals. This aligns with the lattice energy calculations of the cocrystals, i.e., **II**_**CC**_ is less stabilized than **A**_**CC**_ and the SA/FL 1 cocrystal (based on lattice energies compared to the lattice energies of the individual components). The motivation for cocrystallization becomes evident when considering that SG molecules can engage in several strong intermolecular interactions with both SG and FL in the cocrystal.

In the context of the SA/FL 1 cocrystal (hypothetical cocrystal), both the cumulative FL interactions and the strongest API interaction favor cocrystallization. Consequently, in line with the results from CSP, one should anticipate cocrystallization. The observation that identical strong H-bonding motifs, including related conformations, are present in both single- and multicomponent crystals may imply that the choice of the solvent molecule for crystallization could have a significant impact on the outcome. This highlights the importance of a more thorough solvent screening to better understand and optimize (co)crystallization conditions.

It is noteworthy that all cocrystals significantly benefit from aromatic interactions. This observation may provide an explanation for the lack of success of the MCHBP predictions, as this method relies exclusively on the best H-bonding interaction. On the other hand, MEP calculations, which are based on partial charges, agree with the CSP results.

## 4 Conclusions

In this study, we effectively reproduced known cocrystals using traditional screening methods and identified new multicomponent forms, a fifth DDS/FL cocrystal, along with the first SG/FL cocrystals. Hot-melt extrusion, a nonconventional cocrystal screening method, enabled the upscaling of the cocrystal **II**_**CC**_. Moreover, the study successfully resolved the thermodynamic relations of the cocrystal systems at 0 K using a combination of experimental and theoretical approaches.

The experimentally screened cocrystal systems were used to assess virtual cocrystal screening approaches. However, methods such as MCHBP, MEPS, and MC did not yield consistent conclusions about cocrystal formation. The MC method faced limitations, as the size and quantity of heteroatoms proved unreliable as variables for the studied systems. Conflicting results have already been reported for the MC method in other studies, for example in the case of nitrofurantoin cocrystals.^86^ Aromatic interactions emerged as the primary driving force for API/FL cocrystallization, explaining why the MCHBP tool, relying on the best H-bonding interaction, indicated no preferentiality for single- or multicomponent formation for SA/FL and DDS/FL, while SG/FL was even wrongly predicted to crystallize as pure components. In contrast, MEP calculations suggested cocrystal formation for all three systems, consistent with the CSP studies. In agreement with other virtual cocrystal screening outcomes,^86,97−99^ each of the three methods (MCHBP, MEPS, and MC) has its limitations. The primary limitation lies in the fact that none of these methods consider the 3D packing aspect.

Experimentally, the DDS/FL and SG/FL combinations form cocrystals, but SA/FL does not. This raises questions about the adequacy of the experimental search space, especially considering the exceptional stability of the predicted SA/FL cocrystal over single-component crystal structures. Solubility differences between FL and SA posed challenges in experimental approaches, suggesting the need for more specific conditions for cocrystal production. Identical dimeric H-bonding motifs in single-component (SA) and hypothetical multicomponent crystals further indicate the crucial role of crystallization conditions for the SA/FL system. The other two investigated cocrystal systems exhibited more variability in H-bonding/aromatic motifs and packing arrangement of identical motifs.

All three API/FL systems share the characteristic of weaker API interactions (sum of all interactions) in cocrystals compared to single-component crystals. Conversely, FL interactions (sum) are stronger in cocrystals than in FL. This discrepancy was attributed to FL’s lack of H-bond donors. Notably, the coformer, not the API, appears to be the driving force for cocrystallization. Moreover, this study points out that the overall interaction possibility determines cocrystallization.

In conclusion, this multifaceted approach highlights the complexity of cocrystallization processes and provides a valuable strategy for systematically exploring cocrystals.

## Supporting Information Available

Experimental solid form screening: grinding, slurry, hot-melt extrusion, and contact preparation; virtual solid form screening: crystal structure prediction studies, structure similarity dendrogram; pairwise intermolecular energy calculations; solid form characterization: powder X-ray diffraction, IR spectroscopy, moisture sorption desorption; and crystallographic information (PDF)
