# Supplementary material for: Flavone Cocrystals: A Comprehensive Approach Integrating Experimental and Virtual Methods
Source: Cryst Growth Des. 2024 May 6;24(10):4195–212. doi: 10.1021/acs.cgd.4c00293 (PMC11099919; doi:10.1021/acs.cgd.4c00293)
Supplement: Supplementary file 1 — cg4c00293_si_001.pdf [file cg4c00293_si_001.pdf]

Supplementary Information

Flavone Cocrystals: A Comprehensive Approach Integrating Experimental and Virtual Methods

Tom L. Petrick, Alexandra Grünwald, and Doris E. Braun*

Institute of Pharmacy, University of Innsbruck, Innrain 52c, 6020 Innsbruck, Austria

*Email: doris.braun@uibk.ac.at

Table of Contents

A) SOLID FORM SCREENING 5
1. Pre-screening experiments 5
1.1. Hot-Melt Extrusion Optimization: Exploratory Experiments for Identifying Optimal Extrusion Temperatures 5
1.2. API and flavone solubility estimations in (organic) solvents 6
2. Experimental sulfanilamide/flavone cocrystal screening 7
2.1. Grinding experiments 7
2.2. Slurry experiments in organic solvents and water 10
2.3. Hot-melt Extrusion 11
2.4. Contact preparation method 11
3. Experimental dapsone/flavone cocrystal screening 12
3.1. Grinding experiments 12
3.2. Slurry experiments in organic solvents and water 14
3.3. Hot-melt Extrusion 16
3.4. Contact preparation method 16
4. Experimental sulfaguanidine/flavone cocrystal screening 17
4.1. Grinding experiments 17
4.2. Slurry experiments in organic solvents and water 20
4.3. Hot-melt Extrusion 21
4.4. Contact preparation method 22
5. Virtual cocrystal screening 23
5.1. Conformational energy scans 23
5.2. Flavone: conformational analysis 24
5.3. Molecular complementarity (MC) 25
5.4. Molecular Electrostatic potential maps 25
5.5. Multi-component hydrogen-bond propensity 25
6. Computational generation of the single-component and cocrystal energy landscapes 26
6.1. Method - Periodic electronic structure calculations 26
6.2. Calculated low-energy sulfanilamide/flavone structures 26
6.3. Calculated low-energy dapsone/flavone structures 29
6.4. Calculated low-energy sulfaguanidine/flavone structures 30
6.5. Calculated low-energy sulfanilamide structures 31
6.6. Calculated low-energy dapsone structures (recalculated) 33
6.7. Calculated low-energy flavone structures 34
6.8. Crystal packing similarity of predicted cocrystal structures 35
6.8.1. Sulfanilamide/flavone packing similarity dendrogram 35
6.8.2. Dapsone/flavone Dendrogram 36

6.8.3. Sulfaguanidine/flavone Dendrogram 38
B) SOLID-STATE FORM CHARACTERIZATION 39
7. Structure solution form PXRD data 39
8. Flavone polymorphs 42
8.1. Preparation of FL_{II} 42
8.2. Powder X-ray diffraction 42
8.3. IR Spectroscopy 42
8.4. Thermal analysis: Differential scanning calorimetry and hot-stage microscopy 43
8.5. Pawley fit flavone Form III 44
9. Sulfaguanidine/flavone cocrystals 45
9.1. Powder X-ray diffraction 45
9.2. IR spectroscopy 45
9.3. Gravimetric Moisture (de)sorption experiments 46
10. Dapsone/flavone cocrystals 47
10.1. Powder X-ray diffraction 47
10.2. IR spectroscopy 47
10.3. Gravimetric Moisture (de)sorption experiments 49
11. Pairwise intermolecular energy calculations 50
11.1. Flavone form I 50
11.2. Flavone form II 50
11.3. Sulfaguanidine form I 51
11.4. Sulfaguanidine form II 52
11.5. Sulfanilamide form α 52
11.6. Sulfanilamide form β 53
11.7. Sulfanilamide form γ 53
11.8. Sulfanilamide form δ 54
11.9. Dapsone form I 54
11.10. Dapsone form II 55
11.11. Dapsone form III 55
11.12. Dapsone form V 56
11.13. Dapsone/flavone A_{CC} 57
11.14. Dapsone/flavone B_{CC} 57
11.15. Dapsone/flavone D_{CC} 58
11.16. Dapsone/flavone E_{CC} 59
11.17. Sulfaguanidine/flavone II_{CC} 60
11.18. Sulfanilamide/flavone 61
12. References 62

List of abbreviations

Abbreviation	Definition
A_{CC}	Dapsone/flavone cocrystal A (1:1)
B_{CC}	Dapsone/flavone cocrystal B (1:1)
C_{CC}	Dapsone/flavone cocrystal C (1:1)hydrate
DDS	Dapsone
D_{CC}	Dapsone/flavone cocrystal D (1:2)
DDSFL	Dapsone/flavone
DIPE	Diisopropyl ether
E_{CC}	Dapsone/flavone cocrystal E (1:1) *t*-butanol solvate
FL_{A}	Flavone conformation A
FL_{B}	Flavone conformation B
GR	Grinding
H_{DDS}	Dapsone hydrate
HME	Hot-melt extrusion
H_{SA}	Sulfanilamide hydrate
i-BuOAc	Isobutyl acetate
I_{CC}	Sulfaguanidine/flavone cocrystal I (1:1)
I_{DDS}	Dapsone form I
I_{FL}	Flavone form I
II_{CC}	Sulfaguanidine/flavone cocrystal II (1:1)
II_{DDS}	Dapsone form II
II_{FL}	Flavone form II
III_{DDS}	Dapsone form III
III_{SG}	Sulfaguanidine form III
II_{SG}	Sulfaguanidine form II
I_{SG}	Sulfaguanidine form I
IV_{DDS}	Dapsone form IV
IV_{SG}	Sulfaguanidine form IV
SA	Sulfanilamide
SAa	Sulfanilamide conformations A (used in CSP)
SAb	Sulfanilamide conformations B (used in CSP)
SAFL	Sulfanilamide/flavone
SG	Sulfaguanidine
SGa	Sulfaguanidine conformations A (used in CSP)
SGb	Sulfaguanidine conformations B (used in CSP)
SGFL	Sulfaguanidine/flavone
SL	Slurry
t-BuOH	*tert*-Butanol
V_{DDS}	Dapsone form V
V_{SG}	Sulfaguanidine form V
α_{SA}	Sulfanilamide form α
β_{SA}	Sulfanilamide form β
γ_{SA}	Sulfanilamide form γ
δ_{SA}	Sulfanilamide form δ

A) SOLID FORM SCREENING

1. Pre-screening experiments

1.1. Hot-Melt Extrusion Optimization: Exploratory Experiments for Identifying Optimal Extrusion Temperatures

A 1:1 physical mixture comprising the API and flavone was prepared and subjected to DSC analysis, extending up to 10 °C above the melting point of the substance with the higher melting point. The behavior of the mixture and the solidified melt was closely observed throughout the heating process.

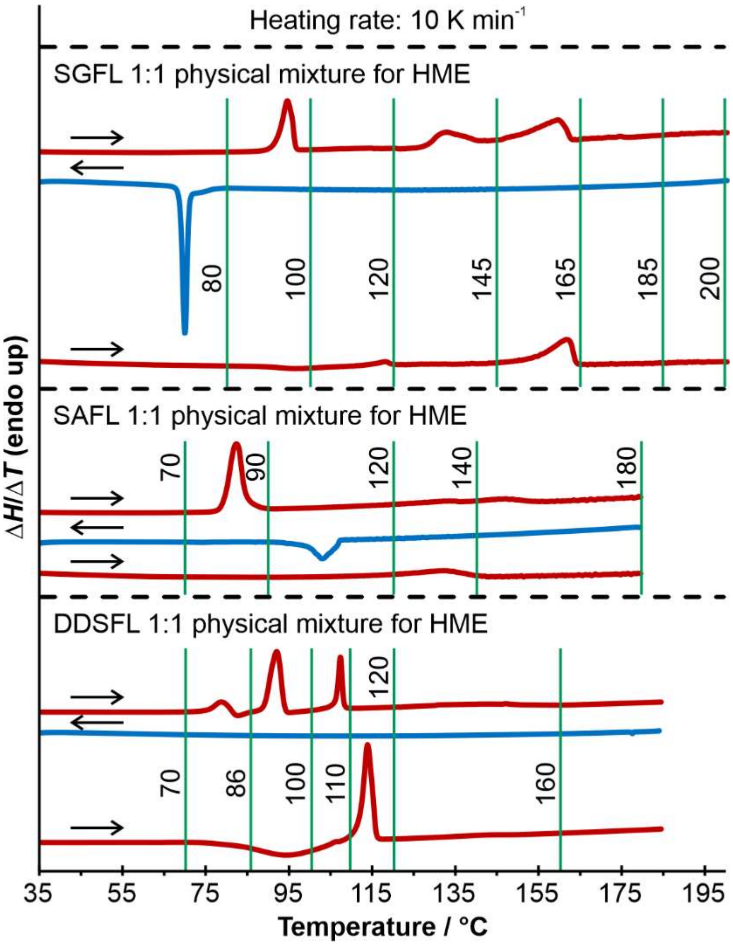

Figure S1. The temperatures selected for the hot-melt extrusion experiments were determined through DSC measurements. The combinations include SGFL (sulfaguanidine and flavone), SAFL (sulfanilamide and flavone), and DDSFL (dapsone and flavone). Chosen temperatures for hot-melt extrusion are represented by green lines. Heating-cooling-heating curves were recorded.

1.2. API and flavone solubility estimations in (organic) solvents

To 15 mg of the substance, solvent was incrementally added in 0.1 mL intervals (up to 1 mL) and subsequently in 0.5 mL increments (up to a maximum of 20 mL). The point at which the substance became visibly dissolved was recorded.

Table S1. Solubility estimations of the investigated substances in different solvents.

**Solvent**	**Dapsone** mg/mL	**Sulfanilamide** mg/mL	**Sulfaguanidine** mg/mL	**Flavone** mg/mL
Acetone	250	159	6.00	153
Acetonitrile	125	74.0	2.52	151
1-Butanol	1.73	2.24	0.75	153
t-Butanol	3.57	1.64	0.76	76.5
Butyl acetate	8.33	4.55	0.75	153
Chloroform	1.00	0.77	0.75	155
Cyclohexane	0.17	0.78	0.76	3.38
Cyclohexanone	54.0	75.9	0.99	150
Dichloromethane	2.94	0.77	0.74	153
Diethyl ether	0.40	0.76	0.75	30.7
Diisopropyl ether	0.42	0.76	0.75	5.08
Dimethyl acetamide	67.5	156	37.4	155
Dimethyl carbonate	10.0	12.5	0.73	157
1,4-Dioxane	7.14	152	0.75	150
Dimethylformamide	260	148	153	151
Dimethyl sulfoxide	260	151	74.5	154
Ethyl acetate	10.0	6.02	0.76	151
Ethanol	7.43	7.53	0.94	75.2
Heptane	0.42	0.74	0.75	3.34
Isobutyl acetate	5.00	2.99	0.75	76.5
Methanol	32.5	50.0	7.50	150
Methyl ethyl ketone	125	52.5	1.48	152
Methyl isobutyl ketone	25.0	9.73	0.74	156
Methyl-*t*-butyl ether	1.25	0.77	0.76	30.2
Nitromethane	13.0	19.1	0.77	153
1-Propanol	4.73	3.02	0.75	75.0
2-Propanol	2.89	3.88	0.78	50.7
Tetrahydrofuran	25.0	51.7	0.76	150
Toluene	0.36	0.77	0.75	153
Water	0.31	3.10	0.75	0.76

2. Experimental sulfanilamide/flavone cocrystal screening

2.1. Grinding experiments

Table S2. Sulfanilamide/flavone liquid-assisted and dry grinding experiments at 15 Hz: SA – sulfanilamide, FL – flavone, α_{SA} – sulfanilamide form α, β_{SA} – sulfanilamide form β, γ_{SA} – sulfanilamide form γ, H_{SA} – sulfanilamide hydrate, II_{FL} – flavone form II, DIPE – Diisopropyl ether, *t*-BuOH – *tert*-Butanol, *i*-BuOAc – isobutyl acetate, * – small quantities detectable.

Solvent	SA / mg	FL / mg	Ratio [SA/FL]	Time / min	Solid-state form				
					α_{SA}	β_{SA}	γ_{SA}	H_{SA}	II_{FL}
DIPE	65.50	84.50	1:1	0		X			X
DIPE	65.50	84.50	1:1	5		X			X
DIPE	65.50	84.50	1:1	10		X			X
DIPE	65.50	84.50	1:1	15		X			X
DIPE	65.50	84.50	1:1	30		X			X
DIPE	65.50	84.50	1:1	45		X			X
DIPE	65.50	84.50	1:1	60		X			X
DIPE	41.95	108.15	1:2	0		X			X
DIPE	41.95	108.15	1:2	5		X			X
DIPE	41.95	108.15	1:2	10		X			X
DIPE	41.95	108.15	1:2	15		X			X
DIPE	41.95	108.15	1:2	30		X			X
DIPE	41.95	108.15	1:2	45		X			X
DIPE	41.95	108.15	1:2	60		X			X
DIPE	91.15	58.85	2:1	0		X			X
DIPE	91.15	58.85	2:1	5		X			X
DIPE	91.15	58.85	2:1	10		X			X
DIPE	91.15	58.85	2:1	15		X			X
DIPE	91.15	58.85	2:1	30		X			X
DIPE	91.15	58.85	2:1	45		X			X
DIPE	91.15	58.85	2:1	60		X			X
n-heptane	65.50	84.50	1:1	0		X			X
n-heptane	65.50	84.50	1:1	5		X			X
n-heptane	65.50	84.50	1:1	10		X			X
n-heptane	65.50	84.50	1:1	15		X			X
n-heptane	65.50	84.50	1:1	30		X			X
n-heptane	65.50	84.50	1:1	45		X			X
n-heptane	65.50	84.50	1:1	60		X			X
n-heptane	42.05	108.15	1:2	0		X			X
n-heptane	42.05	108.15	1:2	5		X			X
n-heptane	42.05	108.15	1:2	10		X			X
n-heptane	42.05	108.15	1:2	15		X			X
n-heptane	42.05	108.15	1:2	30		X			X
n-heptane	42.05	108.15	1:2	45		X			X
n-heptane	42.05	108.15	1:2	60		X			X
n-heptane	91.20	59.00	2:1	0		X			X
n-heptane	91.20	59.00	2:1	5		X			X
n-heptane	91.20	59.00	2:1	10		X			X
n-heptane	91.20	59.00	2:1	15		X			X
n-heptane	91.20	59.00	2:1	30		X			X
n-heptane	91.20	59.00	2:1	45		X			X
n-heptane	91.20	59.00	2:1	60		X			X

Solvent	SA / mg	FL / mg	Ratio [SA/FL]	Time / min	Solid-state form				
					α_{SA}	β_{SA}	γ_{SA}	H_{SA}	II_{FL}
water	65.40	84.60	1:1	0		X			X
water	65.40	84.60	1:1	5		X			X
water	65.40	84.60	1:1	10		X		X	X
water	65.40	84.60	1:1	15		X		X	X
water	65.40	84.60	1:1	30		X		X	X
water	65.40	84.60	1:1	45		X		X	X
water	65.40	84.60	1:1	60		X		X	X
water	41.85	108.20	1:2	0		X			X
water	41.85	108.20	1:2	5		X			X
water	41.85	108.20	1:2	10		X		X	X
water	41.85	108.20	1:2	15		X		X	X
water	41.85	108.20	1:2	30		X		X	X
water	41.85	108.20	1:2	45		X		X	X
water	41.85	108.20	1:2	60		X		X	X
water	91.05	58.75	2:1	0		X			X
water	91.05	58.75	2:1	5		X			X
water	91.05	58.75	2:1	10		X			X
water	91.05	58.75	2:1	15		X		X	X
water	91.05	58.75	2:1	30		X		X	X
water	91.05	58.75	2:1	45		X		X	X
water	91.05	58.75	2:1	60		X		X	X
t-BuOH	65.45	84.60	1:1	0		X			X
t-BuOH	65.45	84.60	1:1	5		X			X
t-BuOH	65.45	84.60	1:1	10		X			X
t-BuOH	65.45	84.60	1:1	15		X			X
t-BuOH	65.45	84.60	1:1	30		X			X
t-BuOH	65.45	84.60	1:1	45		X			X
t-BuOH	65.45	84.60	1:1	60		X			X
t-BuOH	42.00	108.15	1:2	0		X			X
t-BuOH	42.00	108.15	1:2	5		X			X
t-BuOH	42.00	108.15	1:2	10		X			X
t-BuOH	42.00	108.15	1:2	15		X			X
t-BuOH	42.00	108.15	1:2	30		X			X
t-BuOH	42.00	108.15	1:2	45		X			X
t-BuOH	42.00	108.15	1:2	60		X			X
t-BuOH	91.20	58.95	2:1	0		X			X
t-BuOH	91.20	58.95	2:1	5		X			X
t-BuOH	91.20	58.95	2:1	10		X			X
t-BuOH	91.20	58.95	2:1	15		X			X
t-BuOH	91.20	58.95	2:1	30		X			X
t-BuOH	91.20	58.95	2:1	45		X			X
t-BuOH	91.20	58.95	2:1	60		X			X
i-BuOAc	65.45	84.40	1:1	0		X			X
i-BuOAc	65.45	84.40	1:1	5		X			X
i-BuOAc	65.45	84.40	1:1	10		X			X
i-BuOAc	65.45	84.40	1:1	15		X			X
i-BuOAc	65.45	84.40	1:1	30		X			X
i-BuOAc	65.45	84.40	1:1	45		X			X
i-BuOAc	65.45	84.40	1:1	60		X			X

Solvent	SA / mg	FL / mg	Ratio [SA/FL]	Time / min	Solid-state form				
					α_{SA}	β_{SA}	γ_{SA}	H_{SA}	II_{FL}
i-BuOAc	41.95	108.10	1:2	0		X			X
i-BuOAc	41.95	108.10	1:2	5		X			X
i-BuOAc	41.95	108.10	1:2	10		X			X
i-BuOAc	41.95	108.10	1:2	15		X			X
i-BuOAc	41.95	108.10	1:2	30		X			X
i-BuOAc	41.95	108.10	1:2	45		X			X
i-BuOAc	41.95	108.10	1:2	60		X			X
i-BuOAc	91.25	58.80	2:1	0		X			X
i-BuOAc	91.25	58.80	2:1	5		X			X
i-BuOAc	91.25	58.80	2:1	10		X			X
i-BuOAc	91.25	58.80	2:1	15		X			X
i-BuOAc	91.25	58.80	2:1	30		X			X
i-BuOAc	91.25	58.80	2:1	45		X			X
i-BuOAc	91.25	58.80	2:1	60		X			X
Dry	65.50	84.50	1:1	0		X			X
Dry	65.50	84.50	1:1	5		X			X
Dry	65.50	84.50	1:1	10		X			X
Dry	65.50	84.50	1:1	15		X			X
Dry	65.50	84.50	1:1	30		X			X
Dry	65.50	84.50	1:1	45		X			X
Dry	65.50	84.50	1:1	60		X			X
Dry	41.90	108.10	1:2	0		X			X
Dry	41.90	108.10	1:2	5		X			X
Dry	41.90	108.10	1:2	10		X			X
Dry	41.90	108.10	1:2	15		X			X
Dry	41.90	108.10	1:2	30		X			X
Dry	41.90	108.10	1:2	45		X			X
Dry	41.90	108.10	1:2	60		X			X
Dry	91.25	58.95	2:1	0		X			X
Dry	91.25	58.95	2:1	5		X			X
Dry	91.25	58.95	2:1	10		X			X
Dry	91.25	58.95	2:1	15		X			X
Dry	91.25	58.95	2:1	30		X			X
Dry	91.25	58.95	2:1	45		X			X
Dry	91.25	58.95	2:1	60		X			X

2.2. Slurry experiments in organic solvents and water

Table S3. Sulfanilamide/flavone slurry experiments stirred at temperatures cycling between 10 °C and 30 °C: SA – sulfanilamide, FL – flavone, α_{SA} – sulfanilamide form α, β_{SA} – sulfanilamide form β, γ_{SA} – sulfanilamide form γ, H_{SA} – sulfanilamide hydrate, II_{FL} – flavone form II, DIPE – Diisopropyl ether, * – small quantities detectable.

Solvent	SA / mg	FL / mg	Ratio [SA/FL]	Time / days	Solid-state form				
					α_{SA}	β_{SA}	γ_{SA}	H_{SA}	II_{FL}
DIPE	65.50	84.50	1:1	0		X			X
DIPE	65.50	84.50	1:1	1		X			X
DIPE	65.50	84.50	1:1	5		X			X
DIPE	65.50	84.50	1:1	8		X			X
DIPE	65.50	84.50	1:1	21		X			X
DIPE	41.95	108.15	1:2	0		X			X
DIPE	41.95	108.15	1:2	1		X			X
DIPE	41.95	108.15	1:2	5		X			X
DIPE	41.95	108.15	1:2	8		X			X
DIPE	41.95	108.15	1:2	21		X			X
DIPE	91.15	58.85	2:1	0		X			X
DIPE	91.15	58.85	2:1	1		X			X
DIPE	91.15	58.85	2:1	5		X			X
DIPE	91.15	58.85	2:1	8		X			X
DIPE	91.15	58.85	2:1	21		X			X
n-heptane	65.50	84.50	1:1	0		X			X
n-heptane	65.50	84.50	1:1	1		X			X
n-heptane	65.50	84.50	1:1	5		X			X
n-heptane	65.50	84.50	1:1	8		X			X
n-heptane	65.50	84.50	1:1	21		X			X
n-heptane	42.05	108.15	1:2	0		X			X
n-heptane	42.05	108.15	1:2	1		X			X
n-heptane	42.05	108.15	1:2	5		X			X
n-heptane	42.05	108.15	1:2	8		X			X
n-heptane	42.05	108.15	1:2	21		X			X
n-heptane	91.20	59.00	2:1	0		X			X
n-heptane	91.20	59.00	2:1	1		X			X
n-heptane	91.20	59.00	2:1	5		X			X
n-heptane	91.20	59.00	2:1	8		X			X
n-heptane	91.20	59.00	2:1	21		X			X
water	65.40	84.60	1:1	0		X			X
water	65.40	84.60	1:1	1	X*	X			X
water	65.40	84.60	1:1	5		X*	X		X
water	65.40	84.60	1:1	8			X	X	X
water	41.85	108.20	1:2	0		X			X
water	41.85	108.20	1:2	1	X*	X			X
water	41.85	108.20	1:2	5			X		X
water	41.85	108.20	1:2	8				X	X
water	91.05	58.75	2:1	0		X			X
water	91.05	58.75	2:1	1	X*	X			X
water	91.05	58.75	2:1	5		X	X		X
water	91.05	58.75	2:1	8		X	X	X	X

2.3. Hot-melt Extrusion

Table S4. Sulfanilamide/flavone hot-melt extrusion: SA – sulfanilamide, FL – flavone, β_{SA} – sulfanilamide form β, γ_{SA} – sulfanilamide form γ, II_{FL} – flavone form II, III_{FL} – flavone form III, * – small quantities detectable.

	SA / mg	FL / mg	Ratio [SA/FL]	Temperature / °C	Solid-state form			
					β_{SA}	γ_{SA}	II_{FL}	III_{FL}
SA/FL	145.53	187.80	1:1	25	X		X	
SA/FL	145.53	187.80	1:1	70	X		X	
SA/FL	145.53	187.80	1:1	90	X			X
SA/FL	145.53	187.80	1:1	120	X	X		X
SA/FL	145.53	187.80	1:1	140		X		X
SA/FL	145.53	187.80	1:1	180	X*	X		X
SA	300.00		1:0	25	X			
SA	300.00		1:0	70	X			
SA	300.00		1:0	90	X			
SA	300.00		1:0	120	X	X*		
SA	300.00		1:0	140		X		
SA	300.00		1:0	180		X		
FL		300.00	0:1	25			X	
FL		300.00	0:1	70			X	
FL		300.00	0:1	90			X	
FL		300.00	0:1	120			X*	X
FL		300.00	0:1	140			X	X*
FL		300.00	0:1	180			X	

2.4. Contact preparation method

No crystallization was observed for sulfanilamide and flavone.

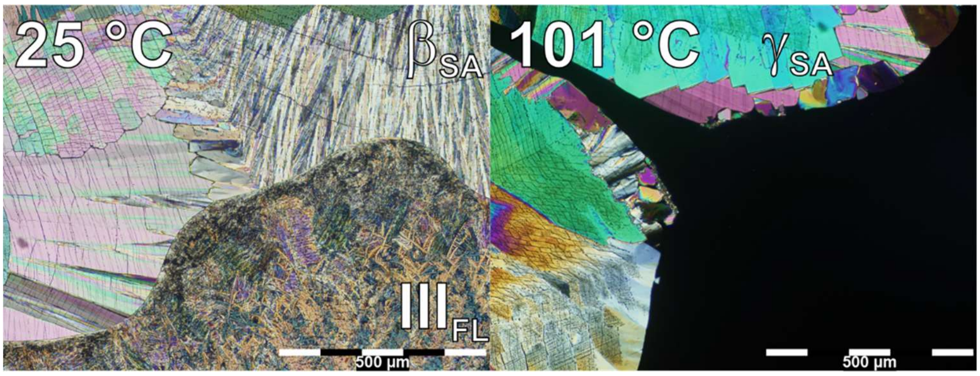

Figure S2. Contact preparation of sulfanilamide/flavone: III_{FL} – flavone form III, β_{SA} – sulfanilamide form β, γ_{SA} – sulfanilamide form γ.

3. Experimental dapsone/flavone cocrystal screening

3.1. Grinding experiments

Table S5. Dapsone/flavone liquid-assisted and dry grinding experiments at 15 Hz: DDS – dapsone, FL – flavone, III_{DDS} – dapsone form III, IV_{DDS} – dapsone form IV, V_{DDS} – dapsone form V, H_{DDS} – dapsone hydrate, II_{FL} – flavone form II, Sol – Solvate formation, A_{CC} – dapsone/flavone cocrystal A (1:1), C_{CC} – dapsone/flavone cocrystal C (1:1), D_{CC} – dapsone/flavone cocrystal D (1:2), DIPE – Diisopropyl ether, *t*-BuOH – *tert*-Butanol, *i*-BuOAc – isobutyl acetate, * – small quantities detectable.

Solvent	Ratio [DDS/FL]	Time / min	Solid-state form						
			III_{DDS}	H_{DDS}	II_{FL}	Sol	A_{CC}	C_{CC}	D_{CC}
DIPE	1:1	0	X		X				
DIPE	1:1	5	X*					X	
DIPE	1:1	10	X*					X	
DIPE	1:1	15	X*					X	
DIPE	1:1	30		X*				X	
DIPE	1:1	45		X*				X	
DIPE	1:1	60		X*				X	
DIPE	1:2	0	X		X				
DIPE	1:2	5	X		X				X
DIPE	1:2	10	X		X				X
DIPE	1:2	15	X		X				X
DIPE	1:2	30							X
DIPE	1:2	45							X
DIPE	1:2	60							X
n-heptane	1:1	0	X		X				
n-heptane	1:1	5	X		X				X
n-heptane	1:1	10	X		X*				X
n-heptane	1:1	15	X						X
n-heptane	1:1	30	X						X
n-heptane	1:1	45	X						X
n-heptane	1:1	60	X						X
n-heptane	1:2	0	X		X				
n-heptane	1:2	5	X		X				X
n-heptane	1:2	10	X		X				X
n-heptane	1:2	15	X		X				X
n-heptane	1:2	30	X		X				X
n-heptane	1:2	45	X		X				X
n-heptane	1:2	60	X		X				X
water	1:1	0	X		X				
water	1:1	5	X		X				X
water	1:1	10	X		X				X
water	1:1	15	X		X				X
water	1:1	30		X				X	
water	1:1	45		X*				X	
water	1:1	60						X	
water	1:2	0	X		X				
water	1:2	5	X		X				X
water	1:2	10	X		X				X
water	1:2	15	X		X				X
water	1:2	30	X		X				X
water	1:2	45			X*			X*	X
water	1:2	60			X*			X*	X

Solvent	Ratio [DDS/FL]	Time / min	Solid-state form						
			III$_{DDS}$	H$_{DDS}$	II$_{FL}$	Sol	A$_{CC}$	C$_{CC}$	D$_{CC}$
t-BuOH	1:1	0	X		X				
t-BuOH	1:1	5	X		X	X			X*
t-BuOH	1:1	10	X		X	X			X*
t-BuOH	1:1	15	X		X	X			X*
t-BuOH	1:1	30	X		X	X	X*		X
t-BuOH	1:1	45	X		X*	X	X		X
t-BuOH	1:1	60	X*			X	X		X
t-BuOH	1:2	0	X		X				
t-BuOH	1:2	5	X		X	X*			X
t-BuOH	1:2	10	X		X	X			X
t-BuOH	1:2	15	X		X	X			X
t-BuOH	1:2	30	X		X	X			X
t-BuOH	1:2	45	X*		X	X			X
t-BuOH	1:2	60			X	X			
i-BuOAc	1:1	0	X		X				
i-BuOAc	1:1	5	X						X
i-BuOAc	1:1	10	X						X
i-BuOAc	1:1	15	X						X
i-BuOAc	1:1	30	X						X
i-BuOAc	1:1	45	X						X
i-BuOAc	1:1	60	X						X
i-BuOAc	1:2	0	X		X				
i-BuOAc	1:2	5	X		X				X
i-BuOAc	1:2	10							X
i-BuOAc	1:2	15							X
i-BuOAc	1:2	30							X
i-BuOAc	1:2	45							X
i-BuOAc	1:2	60							X
dry	1:1	0	X		X				
dry	1:1	5	X		X				X
dry	1:1	10	X		X				X
dry	1:1	15	X						X
dry	1:1	30	X						X
dry	1:1	45	X						X
dry	1:1	60	X						X
dry	1:2	0	X		X				
dry	1:2	5	X		X				X
dry	1:2	10	X		X				X
dry	1:2	15	X		X				X
dry	1:2	30							X
dry	1:2	45							X
dry	1:2	60							X

3.2. Slurry experiments in organic solvents and water

Table S6. Dapsone/flavone slurry experiments stirred at temperatures cycling between 10 °C and 30 °C: DDS – dapsone, FL – flavone, III_{DDS} - dapsone form III, H_{DDS} – dapsone hydrate, II_{FL} – flavone form II, C_{CC} – dapsone/flavone cocrystal C (1:1), D_{CC} – dapsone/flavone cocrystal D (1:2), DIPE – Diisopropyl ether, * – small quantities detectable.

Solvent	DDS / mg	FL / mg	Ratio [DDS/FL]	Time / days	Solid state form				
					III_{DDS}	H_{DDS}	II_{FL}	C_{CC}	D_{CC}
DIPE	52.77	47.23	1:1	0	X		X		
DIPE	52.77	47.23	1:1	1	X				X
DIPE	52.77	47.23	1:1	2	X				X
DIPE	52.77	47.23	1:1	3	X				X
DIPE	35.84	64.16	1:2	0	X		X		
DIPE	35.84	64.16	1:2	1					X
DIPE	35.84	64.16	1:2	2					X
n-heptane	52.77	47.23	1:1	0	X		X		
n-heptane	52.77	47.23	1:1	1	X				X
n-heptane	52.77	47.23	1:1	2	X				X
n-heptane	52.77	47.23	1:1	3	X				X
n-heptane	35.84	64.16	1:2	0	X		X		
n-heptane	35.84	64.16	1:2	1			X*		X
n-heptane	35.84	64.16	1:2	2			X*		X
water	52.77	47.23	1:1	0	X		X		
water	52.77	47.23	1:1	1		X	X	X	X*
water	52.77	47.23	1:1	2		X*	X*	X	
water	35.84	64.16	1:2	0	X		X		
water	35.84	64.16	1:2	1			X*		X
water	35.84	64.16	1:2	2				X*	X

In addition, dapsone/flavone mixtures were subjected to stirring under varying water activities at 25 °C (using methanol/water mixtures).

DDS/FL was stirred (500 r.p.m.) in 3-15 mL of methanol/water mixtures, each containing a different mole fraction of water corresponding to a defined water activity[1, 2] at 25.0 ± 0.1 °C for at least 14 days. Samples were withdrawn periodically, filtered and the resulting phase was determined using powder X-ray diffraction, Karl-Fischer titration, and thermogravimetric analysis.

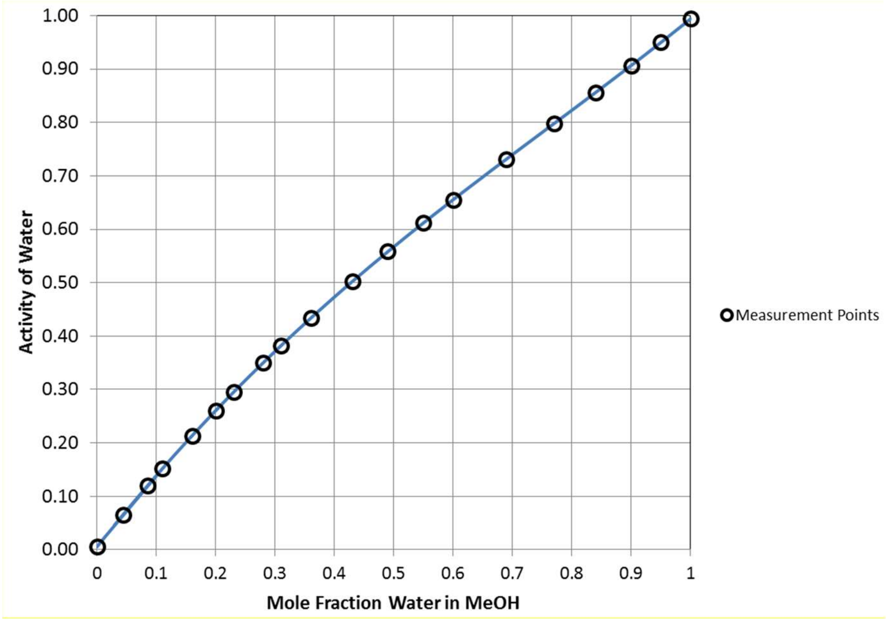

Figure S3. Plot of the water activity versus the mole fraction of water in methanol/water mixtures as 25 °C.

Table S7. Slurry experiments of dapsone/flavone in methanol/water mixtures differing in water activity; B_{CC} – dapsone/flavone cocrystal B (1:1), C_{CC} – dapsone/flavone cocrystal C (1:1), D_{CC} – dapsone/flavone cocrystal D (1:2).

Water activity	DDS/FL Molar ratio	Result	Time (days)
0.23	1:1	B_{CC}	14
0.32	1:1	B_{CC} > D_{CC}	2
0.43	1:1	B_{CC} > D_{CC}	4
0.52	1:1	C_{CC} > D_{CC}	4
0.71	1:1	C_{CC} > D_{CC}	4
0.82	1:1	C_{CC}	3
0.91	1:1	C_{CC}	4

3.3. Hot-melt Extrusion

Table S8. Dapsone/flavone hot-melt extrusion: DDS – dapsone, FL – flavone, III_{DDS} – dapsone form III, II_{FL} – flavone form II, III_{FL} – flavone form III, A_{CC} – dapsone/flavone cocrystal A (1:1), D_{CC} – dapsone/flavone cocrystal D (1:2) , * – small quantities detectable.

DDS / mg	FL / mg	Ratio [DDS/FL]	Temperature / °C	Solid-state form			
				III_{DDS}	II_{FL}	III_{FL}	D_{CC}
113.08	101.21	1:1	25	X	X		
113.08	101.21	1:1	70	X	X		
113.08	101.21	1:1	86	X	X		X*
113.08	101.21	1:1	100	X		X	X
113.08	101.21	1:1	110	X			X
113.08	101.21	1:1	120	X			X
113.08	101.21	1:1	160	X			X

3.4. Contact preparation method

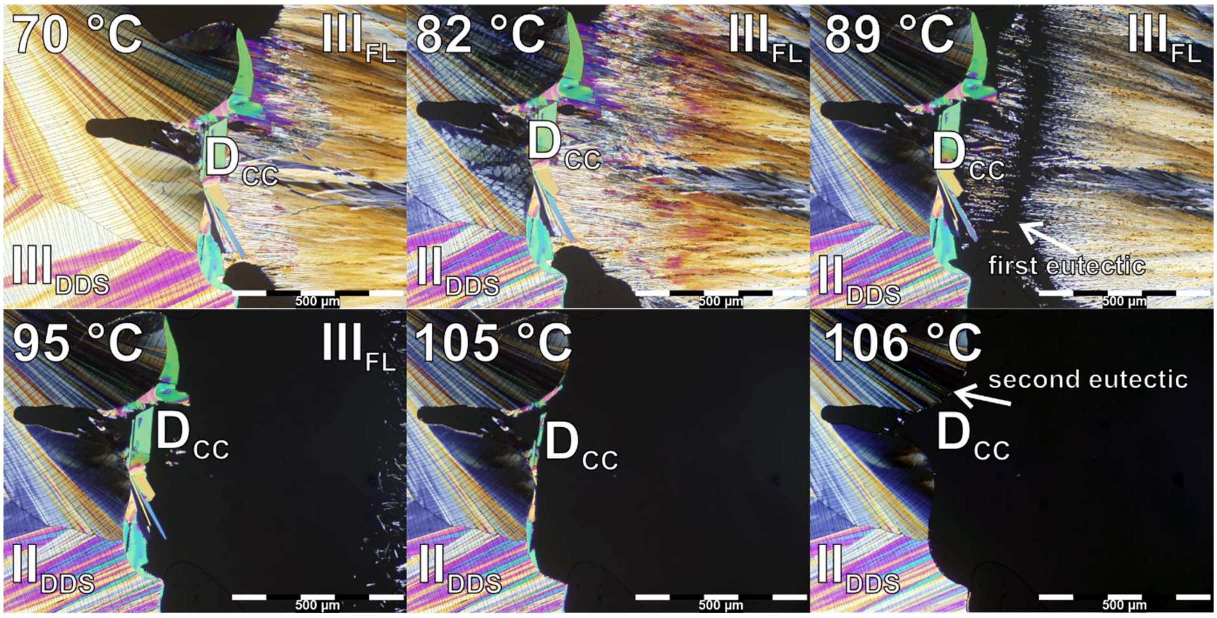

Figure S4. Contact preparation of dapsone/flavone: II_{DDS} – dapsone form II, III_{DDS} – dapsone form III, III_{FL} – flavone form III, D_{CC} – dapsone/flavone cocrystal D (1:2).

4. Experimental sulfaguanidine/flavone cocrystal screening
4.1. Grinding experiments

Table S9. Sulfaguanidine/flavone liquid-assisted and dry grinding experiments at 15 Hz: SG – sulfaguanidine, FL – flavone, I_{SG} – sulfaguanidine form I, H_{SG} – sulfaguanidine hydrate, II_{FL} – flavone form II, I_{CC} – sulfaguanidine/flavone cocrystal I (1:1), II_{CC} – sulfaguanidine/flavone cocrystal II (1:1), DIPE – Diisopropyl ether, *t*-BuOH – *tert*-Butanol, *i*-BuOAc – isobutyl acetate, * – small quantities detectable.

Solvent	SG / mg	FL / mg	Ratio [SG/FL]	Time / min	Solid-state form				
					I_{SG}	H_{SG}	II_{FL}	I_{CC}	II_{CC}
DIPE	73.50	76.25	1:1	0	X		X		
DIPE	73.50	76.25	1:1	5	X	X	X		X*
DIPE	73.50	76.25	1:1	10	X	X	X		X*
DIPE	73.50	76.25	1:1	15	X	X*	X		X
DIPE	73.50	76.25	1:1	30	X		X		X
DIPE	73.50	76.25	1:1	45	X		X		X
DIPE	73.50	76.25	1:1	60	X		X		X
DIPE	48.85	101.25	1:2	0	X		X		
DIPE	48.85	101.25	1:2	5	X*	X			X*
DIPE	48.85	101.25	1:2	10	X	X			X*
DIPE	48.85	101.25	1:2	15	X	X*			X*
DIPE	48.85	101.25	1:2	30	X			X	X*
DIPE	48.85	101.25	1:2	45	X*			X	X*
DIPE	48.85	101.25	1:2	60				X	X*
DIPE	98.65	51.00	2:1	0	X		X		
DIPE	98.65	51.00	2:1	5	X	X	X		X*
DIPE	98.65	51.00	2:1	10	X	X	X		X
DIPE	98.65	51.00	2:1	15	X	X	X		X
DIPE	98.65	51.00	2:1	30	X	X		X	X
DIPE	98.65	51.00	2:1	45		X		X	X
DIPE	98.65	51.00	2:1	60		X		X	X
n-heptane	73.55	76.30	1:1	0	X		X		
n-heptane	73.55	76.30	1:1	5	X	X	X		
n-heptane	73.55	76.30	1:1	10	X	X	X		
n-heptane	73.55	76.30	1:1	15	X	X	X		X*
n-heptane	73.55	76.30	1:1	30	X	X*	X		X
n-heptane	73.55	76.30	1:1	45	X		X	X*	X
n-heptane	73.55	76.30	1:1	60	X		X	X	X
n-heptane	48.85	101.20	1:2	0	X		X		
n-heptane	48.85	101.20	1:2	5	X*	X	X		
n-heptane	48.85	101.20	1:2	10	X*	X	X		
n-heptane	48.85	101.20	1:2	15	X	X	X		
n-heptane	48.85	101.20	1:2	30	X	X*	X		X*
n-heptane	48.85	101.20	1:2	45	X		X	X*	X*
n-heptane	48.85	101.20	1:2	60	X		X	X	X
n-heptane	61.55	31.90	2:1	0	X		X		X
n-heptane	61.55	31.90	2:1	5	X	X	X		X
n-heptane	61.55	31.90	2:1	10	X	X	X		X
n-heptane	61.55	31.90	2:1	15	X	X*	X		X
n-heptane	61.55	31.90	2:1	30	X				X
n-heptane	61.55	31.90	2:1	45	X				X
n-heptane	61.55	31.90	2:1	60	X			X*	X

Solvent	SG / mg	FL / mg	Ratio [SG/FL]	Time / min	Solid-state form				
					I_{SG}	H_{SG}	II_{FL}	I_{CC}	II_{CC}
water	73.60	76.40	1:1	0	X		X		
water	73.60	76.40	1:1	5		X	X		
water	73.60	76.40	1:1	10		X	X		
water	73.60	76.40	1:1	15		X	X		
water	73.60	76.40	1:1	30		X	X		
water	73.60	76.40	1:1	45		X	X		
water	73.60	76.40	1:1	60		X	X		
water	48.70	101.25	1:2	0	X		X		
water	48.70	101.25	1:2	5		X	X		
water	48.70	101.25	1:2	10		X	X		
water	48.70	101.25	1:2	15		X	X		
water	48.70	101.25	1:2	30		X	X		
water	48.70	101.25	1:2	45		X	X		
water	48.70	101.25	1:2	60		X	X		
water	98.70	51.20	2:1	0	X		X		
water	98.70	51.20	2:1	5		X	X		
water	98.70	51.20	2:1	10		X	X		
water	98.70	51.20	2:1	15		X	X		
water	98.70	51.20	2:1	30		X	X		
water	98.70	51.20	2:1	45		X	X		
water	98.70	51.20	2:1	60		X	X		
t-BuOH	73.60	76.35	1:1	0	X		X		
t-BuOH	73.60	76.35	1:1	5	X		X	X*	
t-BuOH	73.60	76.35	1:1	10	X		X	X	
t-BuOH	73.60	76.35	1:1	15	X		X	X	
t-BuOH	73.60	76.35	1:1	30	X		X*	X	
t-BuOH	73.60	76.35	1:1	45	X*		X*	X	
t-BuOH	73.60	76.35	1:1	60				X	
t-BuOH	48.85	101.25	1:2	0	X		X		
t-BuOH	48.85	101.25	1:2	5	X		X	X	
t-BuOH	48.85	101.25	1:2	10	X		X	X	
t-BuOH	48.85	101.25	1:2	15	X		X	X	
t-BuOH	48.85	101.25	1:2	30	X*		X	X	
t-BuOH	48.85	101.25	1:2	45			X	X	
t-BuOH	48.85	101.25	1:2	60			X	X	
t-BuOH	98.70	51.20	2:1	0	X		X		
t-BuOH	98.70	51.20	2:1	5	X		X	X	
t-BuOH	98.70	51.20	2:1	10	X		X	X	
t-BuOH	98.70	51.20	2:1	15	X			X	
t-BuOH	98.70	51.20	2:1	30	X	X		X	
t-BuOH	98.70	51.20	2:1	45		X		X	
t-BuOH	98.70	51.20	2:1	60		X		X	
i-BuOAc	73.50	76.30	1:1	0	X		X		
i-BuOAc	73.50	76.30	1:1	5	X		X	X*	X
i-BuOAc	73.50	76.30	1:1	10	X		X	X	X
i-BuOAc	73.50	76.30	1:1	15	X		X	X	X
i-BuOAc	73.50	76.30	1:1	30	X		X	X	X*
i-BuOAc	73.50	76.30	1:1	45	X*		X*	X	X*
i-BuOAc	73.50	76.30	1:1	60				X	

Solvent	SG / mg	FL / mg	Ratio [SG/FL]	Time / min	Solid-state form				
					I_{SG}	H_{SG}	II_{FL}	I_{CC}	II_{CC}
i-BuOAc	48.70	101.15	1:2	0	X		X		
i-BuOAc	48.70	101.15	1:2	5	X		X		X*
i-BuOAc	48.70	101.15	1:2	10	X		X	X*	X*
i-BuOAc	48.70	101.15	1:2	15	X		X	X	X*
i-BuOAc	48.70	101.15	1:2	30			X	X	
i-BuOAc	48.70	101.15	1:2	45			X	X	
i-BuOAc	48.70	101.15	1:2	60			X	X	
i-BuOAc	98.85	51.25	2:1	0	X		X		
i-BuOAc	98.85	51.25	2:1	5	X		X		X
i-BuOAc	98.85	51.25	2:1	10	X	X*		X	X
i-BuOAc	98.85	51.25	2:1	15	X*	X		X	X
i-BuOAc	98.85	51.25	2:1	30		X		X	X*
i-BuOAc	98.85	51.25	2:1	45		X		X	
i-BuOAc	98.85	51.25	2:1	60		X		X	
dry	73.65	76.40	1:1	0	X		X		
dry	73.65	76.40	1:1	5	X	X*	X		
dry	73.65	76.40	1:1	10	X		X		
dry	73.65	76.40	1:1	15	X		X		
dry	73.65	76.40	1:1	30	X		X		X*
dry	73.65	76.40	1:1	45	X		X		X*
dry	73.65	76.40	1:1	60	X		X		X*
dry	48.70	101.15	1:2	0	X		X		
dry	48.70	101.15	1:2	5	X		X		
dry	48.70	101.15	1:2	10	X		X		
dry	48.70	101.15	1:2	15	X		X		
dry	48.70	101.15	1:2	30	X		X		
dry	48.70	101.15	1:2	45	X		X		
dry	48.70	101.15	1:2	60	X		X		X*
dry	98.65	51.15	2:1	0	X		X		
dry	98.65	51.15	2:1	5	X	X*	X		
dry	98.65	51.15	2:1	10	X	X*	X		
dry	98.65	51.15	2:1	15	X	X*	X		
dry	98.65	51.15	2:1	30	X		X		X*
dry	98.65	51.15	2:1	45	X		X		X
dry	98.65	51.15	2:1	60	X		X		X

4.2. Slurry experiments in organic solvents and water

Table S10. Sulfaguanidine/flavone slurry experiments stirred at temperatures cycling between 10 °C and 30 °C: SG – sulfaguanidine, FL – flavone, I_{SG} – sulfaguanidine form I, H_{SG} – sulfaguanidine hydrate, II_{FL} – flavone form II, I_{CC} – sulfaguanidine/flavone cocrystal I (1:1), II_{CC} – sulfaguanidine/flavone cocrystal II (1:1), DIPE – Diisopropyl ether, * – small quantities detectable.

Solvent	SG / mg	FL / mg	Ratio [SG/FL]	Time / days	Solid-state form				
					I_{SG}	H_{SG}	II_{FL}	I_{CC}	II_{CC}
DIPE	49.10	51.00	1:1	0	X		X		
DIPE	49.10	51.00	1:1	1	X		X	X	X
DIPE	49.10	51.00	1:1	2	X*		X	X	X
DIPE	49.10	51.00	1:1	3			X	X	
DIPE	49.10	51.00	1:1	7			X*	X	
DIPE	49.10	51.00	1:1	11				X	
DIPE	32.45	67.40	1:2	0	X		X		
DIPE	32.45	67.40	1:2	1	X*		X	X*	X
DIPE	32.45	67.40	1:2	2			X	X	X
DIPE	32.45	67.40	1:2	3			X	X	X*
DIPE	32.45	67.40	1:2	7			X	X	
DIPE	32.45	67.40	1:2	11			X	X	
DIPE	65.85	34.15	2:1	0	X		X		
DIPE	65.85	34.15	2:1	1	X		X*	X	X
DIPE	65.85	34.15	2:1	2	X*	X		X	X*
DIPE	65.85	34.15	2:1	3		X		X	
DIPE	65.85	34.15	2:1	7		X		X	
DIPE	65.85	34.15	2:1	11		X		X	
n-heptane	49.10	51.00	1:1	0	X		X		
n-heptane	49.10	51.00	1:1	1	X		X	X*	
n-heptane	49.10	51.00	1:1	2	X		X	X	
n-heptane	49.10	51.00	1:1	3	X		X	X	
n-heptane	49.10	51.00	1:1	7	X		X	X	
n-heptane	49.10	51.00	1:1	11	X		X	X	
n-heptane	32.45	67.40	1:2	0	X		X		
n-heptane	32.45	67.40	1:2	1	X		X	X*	
n-heptane	32.45	67.40	1:2	2	X		X	X*	
n-heptane	32.45	67.40	1:2	3	X		X	X	
n-heptane	32.45	67.40	1:2	7	X		X	X	
n-heptane	32.45	67.40	1:2	11	X		X	X	
n-heptane	65.95	34.20	2:1	0	X		X		
n-heptane	65.95	34.20	2:1	1	X		X	X	
n-heptane	65.95	34.20	2:1	2	X		X*	X	
n-heptane	65.95	34.20	2:1	3	X	X*		X	
n-heptane	65.95	34.20	2:1	7	X	X*		X	
n-heptane	65.95	34.20	2:1	11	X	X*		X	
water	49.25	51.10	1:1	0	X		X		
water	49.25	51.10	1:1	1		X	X		
water	49.25	51.10	1:1	2		X	X		
water	49.25	51.10	1:1	3		X	X		
water	49.25	51.10	1:1	7		X	X		
water	49.25	51.10	1:1	11		X	X		

Solvent	SG / mg	FL / mg	Ratio [SG/FL]	Time / days	Solid-state form				
					I_{SG}	H_{SG}	II_{FL}	I_{CC}	II_{CC}
water	32.50	67.45	1:2	0	X		X		
water	32.50	67.45	1:2	1		X	X		
water	32.50	67.45	1:2	2		X	X		
water	32.50	67.45	1:2	3		X	X		
water	32.50	67.45	1:2	7		X	X		
water	32.50	67.45	1:2	11		X	X		
water	66.20	34.50	2:1	0	X		X		
water	66.20	34.50	2:1	1		X	X		
water	66.20	34.50	2:1	2		X	X		
water	66.20	34.50	2:1	3		X	X		
water	66.20	34.50	2:1	7		X	X		
water	66.20	34.50	2:1	11		X	X		

4.3. Hot-melt Extrusion

Table S11. Sulfaguanidine/flavone hot-melt extrusion: SG – sulfaguanidine, FL – flavone, I_{SG} – sulfaguanidine form I, H_{SG} – sulfaguanidine hydrate, II_{FL} – flavone form II, III_{FL} – flavone form III, I_{CC} – sulfaguanidine/flavone cocrystal I (1:1), II_{CC} – sulfaguanidine/flavone cocrystal II (1:1) , * – small quantities detectable.

SG / mg	FL / mg	Ratio [SG/FL]	Temper-ature / °C	Solid state form					
				I_{SG}	H_{SG}	II_{FL}	III_{FL}	I_{CC}	II_{CC}
92.03	95.47	1:1	25		X	X			
92.03	95.47	1:1	80		X	X			
92.03	95.47	1:1	100		X	X	X	X*	
92.03	95.47	1:1	120		X	X*	X	X	X*
92.03	95.47	1:1	145	X*			X	X	X
92.03	95.47	1:1	165				X	X*	X
92.03	95.47	1:1	185				X		X
92.03	95.47	1:1	200						X

4.4. Contact preparation method

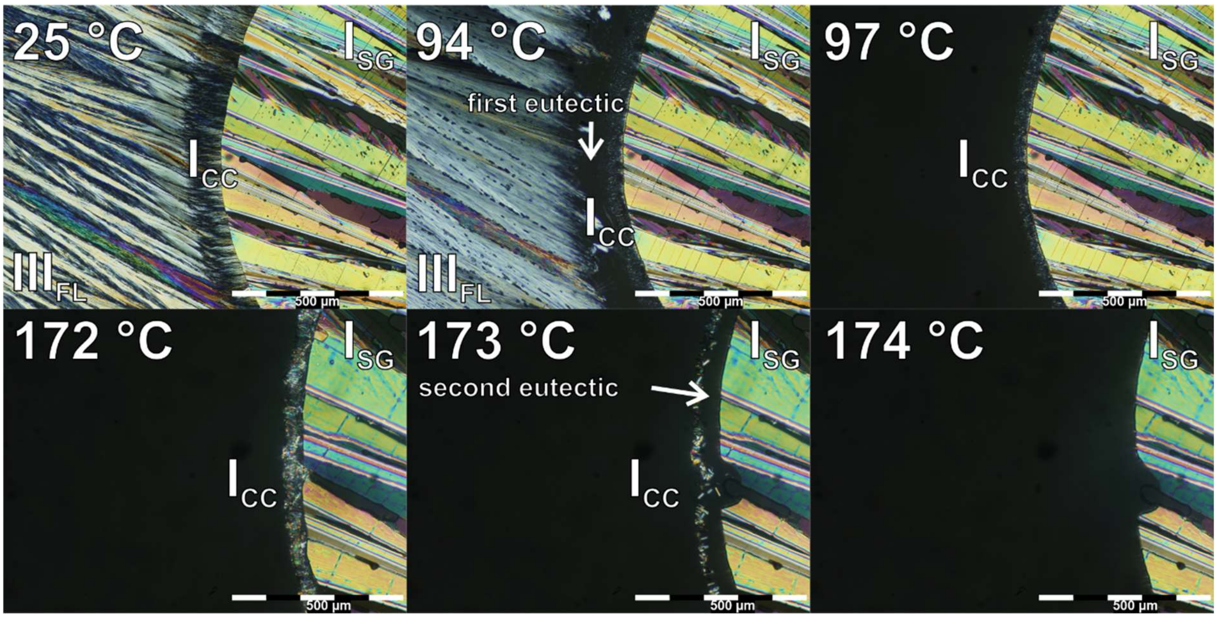

Figure S5. Contact preparation of sulfaguanidine/flavone: I_{SG} – sulfaguanidine form I, III_{FL} – flavone form III, I_{CC} – sulfaguanidine/flavone cocrystal I (1:1).

5. Virtual cocrystal screening

5.1. Conformational energy scans

Conformational energy scans [using PBE0/6-31G(d,p)] were conducted to determine the low-energy conformations of the three APIs and FL. The dihedrals for sulfanilamide and sulfaguanidine were scanned with a 30° increment size, while DDS was scanned with a 20° increment size, and flavone with a 15° increment size. The red “X” on the plots represent the selected conformations used for the virtual screening.

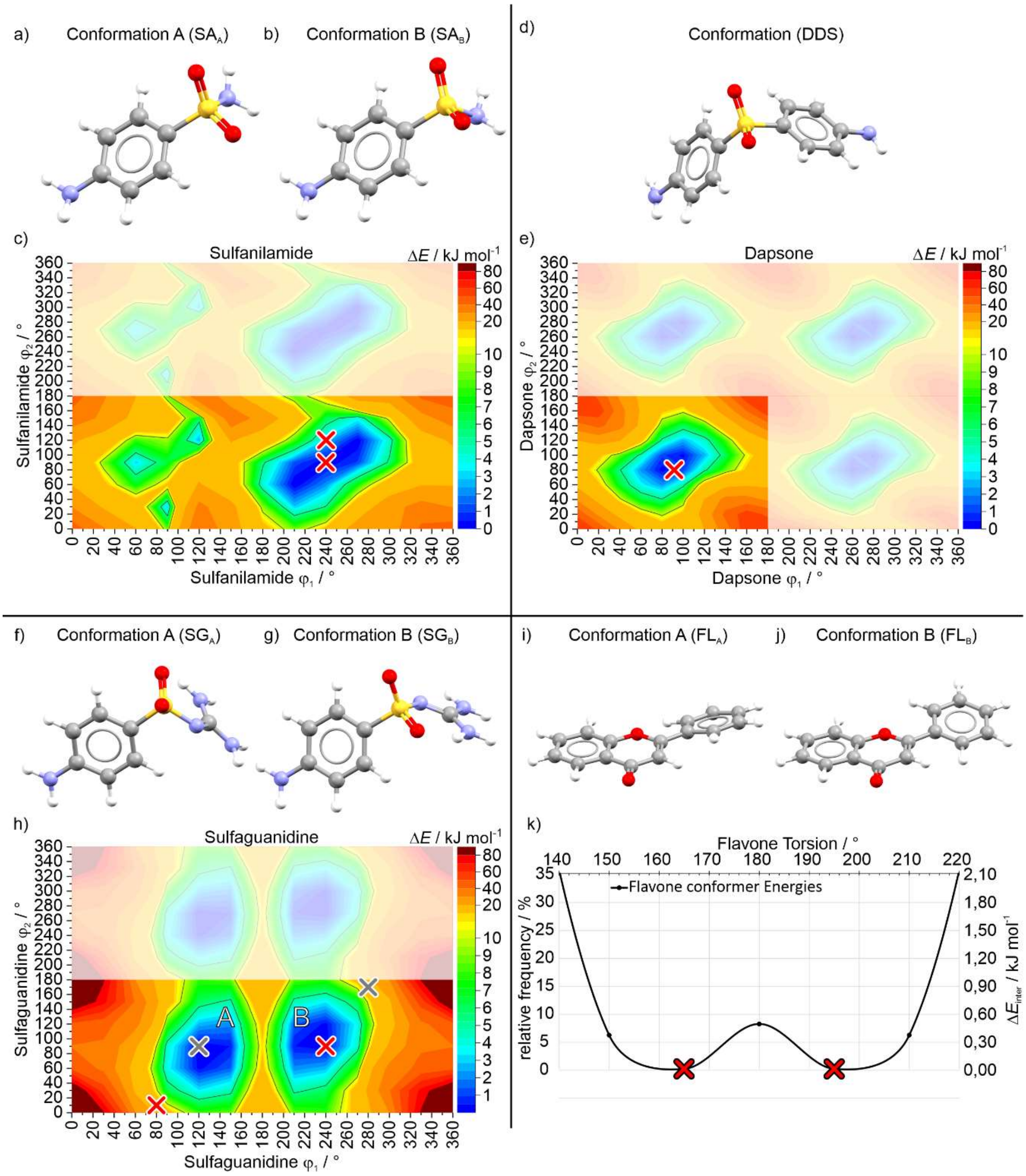

Figure S6. Potential energy scans of the four chosen molecules and conformations chosen as input for the virtual cocrystal screening methods: a) and b) conformations of sulfanilamide, (c) sulfanilamide PES scan with chosen conformations highlighted, d) dapsone conformation, e) dapsone PES scan with chosen conformations highlighted, f) and g) conformation of sulfaguanidine A (SG_A) and B (SG_B), h) sulfaguanidine PES scan with chosen conformations highlighted in red and alternative conformations seen in centrosymmetric space groups in grey, i) and j) conformation of flavone A (FL_A) and B (FL_B), k) flavone PES scan with chosen conformations highlighted.

5.2. Flavone: conformational analysis

Graphic 6 shows the relative frequencies of the flavone dihedrals found in the sulfanilamide/flavone, sulfaguanidine/flavone, and dapsone/flavone cocrystals generated by CSP.

The PES scan of the FL φ_1 dihedral angle (Figure S7) reveals that the phenyl ring can rotate significantly with minimal intramolecular energy cost. This observation explains why the φ_1 angle values vary between 140° and 190° in the predicted structures. Furthermore, the analysis reveals two energy minima at 160° and 210°, each twisted 20° away from the planar conformation. The planar structure represents a local maximum, albeit only 0.5 kJ mol^{-1} higher in energy than the global minima. None of the experimental and predicted FL conformers deviate by more than 0.5 kJ mol^{-1} in intramolecular energy from the global minimum.

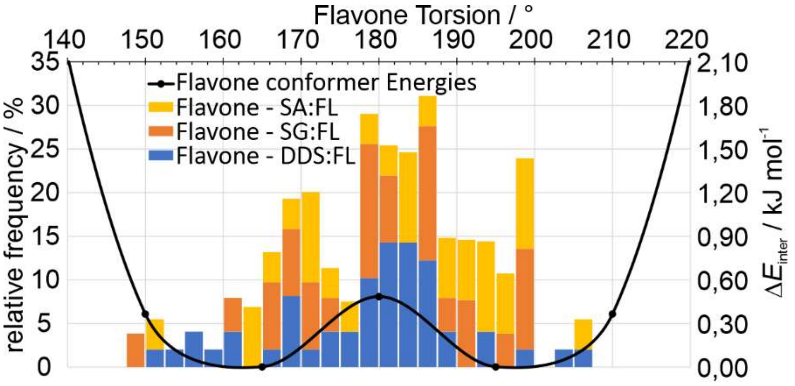

Figure S7 - Potential energy surface scan of flavone (black) with the relative frequency of the conformations of FL observed in the computationally generated structures (sulfanilamide/flavone yellow; sulfaguanidine/flavone orange; dapsone/flavone blue).

5.3. Molecular complementarity (MC)

Table S12. Virtual cocrystal screening: Molecular complementarity; FL_A – flavone conformer A, FL_B – flavone conformer B, SA_A – sulfanilamide conformer A, SA_B – sulfanilamide conformer B, DDS – dapsone, SG_A – sulfaguanidine conformer A, SG_B – sulfaguanidine conformer B, fail/pass – result of the MC test, Δ*M*/*L*, Δ*S*, and Δ*S*/*L* – size desciptors in the MC test, ΔDipol – descriptor test to dipol moment differences, ΔNO – descriptor for N and O atom differences.

Combinations API/coformer	fail/pass	Molecular complementarity tests Δ*M*/*L*	Δ*S*	Δ*S*/*L*	ΔDipol	ΔNO
SA_A/FL_A	pass	pass	pass	pass	pass	pass
SA_A/FL_B	pass	pass	pass	pass	pass	pass
SA_B/FL_A	fail	pass	pass	fail	pass	pass
SA_B/FL_B	fail	pass	pass	fail	pass	pass
DDS/FL_A	pass	pass	pass	pass	pass	pass
DDS/FL_B	pass	pass	pass	pass	pass	pass
SG_A/FL_A	fail	pass	pass	fail	pass	fail
SG_A/FL_B	fail	pass	pass	fail	pass	fail
SG_B/FL_A	fail	pass	pass	pass	pass	fail
SG_B/FL_B	fail	pass	pass	pass	pass	fail

5.4. Molecular Electrostatic potential maps

Table S13. Calculated ΔE values based on MEP calculations: FL – flavone, SA – sulfanilamide, DDS – dapsone, SG – sulfaguanidine.

Ratio / API	FL 1:1	1:2	2:1
**SA**	-12 to -13	-23	-13
**DDS**	-14	-24	-15
**SG**	-12 to -13	-22 to -25	-13

The values for various conformations of SA_A and SA_B, or SG_A and SG_B, with FL_A and FL_B exhibit only marginal differences. Hence, these conformations have been consolidated in the table.

5.5. Multi-component hydrogen-bond propensity

Table S14. Virtual cocrystal screening: multi-component hydrogen-bond propensity results; A – API, C – coformer (flavone).

API (A)	Multi-component score	max interaction	max C/A or A/C propensity	max C/C propensity	max A/A propensity
Sulfanilamide	-0.01	A/A	0.63	0.00	0.64
Dapsone	-0.01	A/A	0.66	0.00	0.67
Sulfaguanidine	-0.25	A/A	0.55	0.00	0.80

6. Computational generation of the single-component and cocrystal energy landscapes

6.1. Method - Periodic electronic structure calculations

The maximum spacing was set to 2π0.07 $Å^{-1}$, a basis cutoff of 560 eV for single point calculations or 780 eV for optimizations and the MDB* dispersion correction was used. The structures were considered as converged if the energy change was less than $2x10^{-5}$ $eVÅ^{-1}$, the atomic displacement less than $1x10^{-3}$ $Å^{-1}$, and the maximum stress less than 0.1 GPa.

The following section provides the CSP results (PBE-MBD*) in a tabulated form.

6.2. Calculated low-energy sulfanilamide/flavone structures

Table S15. Sulfanilamide/flavone: Computationally generated low-energy PBE-MBD* cocrystal structures.

Structure	Space group	Cell parameters						Energy	Packing
		a / Å	*b* / Å	*c* / Å	α / °	β / °	γ / °	kJ mol^{-1}	Index
1	*Pbca*	21.52	11.36	14.95	90.0	90.0	90.0	-589754.84	0.73
2	*Pbca*	8.27	20.46	21.63	90.0	90.0	90.0	-589747.05	0.72
3	$P2_1/c$	7.97	23.65	9.92	90.0	74.6	90.0	-589746.12	0.74
4	*P*-1	9.39	9.87	11.25	107.6	91.6	68.2	-589745.68	0.72
5	$P2_1/c$	8.35	22.78	9.88	90.0	72.8	90.0	-589743.63	0.74
6	*P*-1	8.38	11.45	10.16	93.1	93.0	73.6	-589742.05	0.71
7	$P2_1/c$	7.82	8.06	32.11	90.0	118.0	90.0	-589741.93	0.74
8	$P2_1/c$	11.44	10.27	15.65	90.0	83.4	90.0	-589741.55	0.73
9	$P2_1/c$	7.87	8.10	31.70	90.0	117.5	90.0	-589741.53	0.75
10	$P2_1/c$	16.39	12.41	9.30	90.0	107.0	90.0	-589741.53	0.74
11	*P*-1	8.25	11.39	10.99	71.4	72.4	80.0	-589740.72	0.71
12	*Pbca*	22.56	10.09	16.04	90.0	90.0	90.0	-589740.12	0.73
13	$P2_1/c$	10.65	13.88	12.90	90.0	76.6	90.0	-589739.86	0.72
14	$P2_1/c$	5.09	29.79	14.41	90.0	56.0	90.0	-589739.82	0.74
15	*P*-1	8.23	11.37	10.91	82.4	93.8	76.8	-589739.74	0.68
16	$P2_1/c$	10.55	11.46	15.83	90.0	83.2	90.0	-589739.35	0.70
17	$P2_1/c$	8.60	23.61	10.03	90.0	114.7	90.0	-589739.35	0.72
18	*Cc*	16.71	5.31	21.13	90.0	76.6	90.0	-589739.18	0.73
19	$P2_1/c$	11.73	9.43	16.82	90.0	95.9	90.0	-589739.14	0.72
20	*P*-1	11.76	7.92	12.52	115.3	61.8	103.6	-589738.85	0.72
21	*P*-1	8.22	12.87	9.28	91.3	72.9	74.8	-589738.62	0.74
22	$P2_1/c$	15.35	5.05	24.42	90.0	73.2	90.0	-589738.61	0.73
23	$P2_1/c$	7.84	20.92	12.01	90.0	68.4	90.0	-589738.25	0.72
24	$P2_1/c$	8.78	27.37	8.66	90.0	119.3	90.0	-589738.03	0.73
25	*P*-1	9.29	10.74	11.15	65.1	111.4	111.1	-589737.94	0.73
26	$P2_12_12_1$	22.27	15.37	5.15	90.0	90.0	90.0	-589737.85	0.76
27	$P2_12_12_1$	21.73	16.67	5.05	90.0	90.0	90.0	-589737.47	0.73
28	$P2_1/c$	5.21	15.59	22.49	90.0	79.4	90.0	-589736.52	0.74
29	$P2_1/c$	10.24	23.86	8.17	90.0	65.7	90.0	-589734.95	0.73

Figure S8 shows the crystal packing of the lowest energy sulfanilamide/flavone cocrystal (ID: *1*).

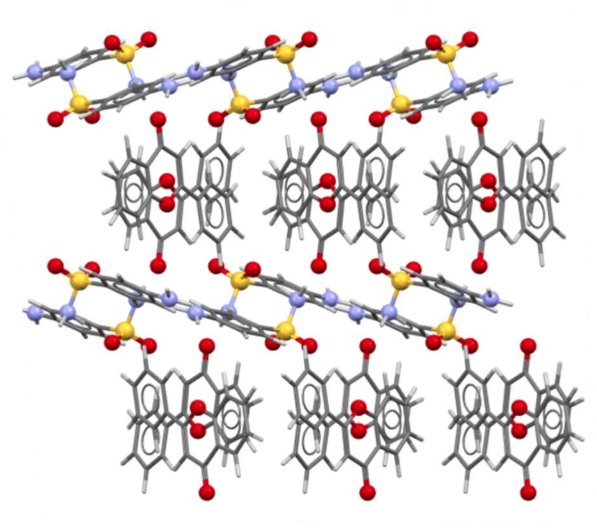

Figure S8. Packing diagram of the lowest-energy sulfanilamide/flavone structure, viewed along the crystallographic *b*-axis.

The *.res file for the lowest-energy sulfanilamide/flavone cocrystal structure is given below:

```
TITL Sulfanilamide/flavone cocrystal
CELL 0.71073 21.5186 11.3594 14.9526 90 90 90
ZERR 8 0 0 0 0 0 0
LATT 1
SYMM 1/2+x,1/2-y,-z
SYMM -x,1/2+y,1/2-z
SYMM 1/2-x,-y,1/2+z
SFAC C H N O S
H1     2 -0.011074  0.276465  0.776235  1.000000 0.010000
H2     2  0.004555  0.127694  0.892223  1.000000 0.010000
H3     2  0.083702  0.390347  1.066523  1.000000 0.010000
H4     2  0.067931  0.541709  0.949200  1.000000 0.010000
H5     2  0.036097  0.585589  0.797544  1.000000 0.010000
H6     2  0.003473  0.476669  0.727308  1.000000 0.010000
H7     2 -0.041498  0.175495  1.130163  1.000000 0.010000
H8     2 -0.037223  0.055193  1.059794  1.000000 0.010000
C1     1  0.027463  0.420613  0.853695  1.000000 0.010000
C2     1  0.010252  0.301740  0.839346  1.000000 0.010000
C3     1  0.019149  0.217991  0.904843  1.000000 0.010000
C4     1  0.046043  0.249571  0.986917  1.000000 0.010000
C5     1  0.063397  0.366394  1.002385  1.000000 0.010000
C6     1  0.054746  0.450542  0.936649  1.000000 0.010000
N1     3  0.015360  0.504209  0.790647  1.000000 0.010000
N2     3 -0.013902  0.105346  1.107166  1.000000 0.010000
O1     4  0.080635  0.036520  1.031290  1.000000 0.010000
O2     4  0.088006  0.197383  1.145109  1.000000 0.010000
S1     5  0.055371  0.144021  1.070832  1.000000 0.010000
```

H9 2 0.139783 0.549214 0.726039 1.000000 0.010000
H10 2 0.126467 0.889860 0.924537 1.000000 0.010000
H11 2 0.205708 1.014580 0.994638 1.000000 0.010000
H12 2 0.318296 0.968767 0.973262 1.000000 0.010000
H13 2 0.350058 0.797767 0.878956 1.000000 0.010000
H14 2 0.357282 0.571247 0.717255 1.000000 0.010000
H15 2 0.409183 0.414533 0.630680 1.000000 0.010000
H16 2 0.347488 0.251930 0.563499 1.000000 0.010000
H17 2 0.232483 0.247799 0.583483 1.000000 0.010000
H18 2 0.180415 0.399212 0.672160 1.000000 0.010000
C7 1 0.234487 0.588265 0.754568 1.000000 0.010000
C8 1 0.171720 0.605722 0.762622 1.000000 0.010000
C9 1 0.146525 0.697557 0.816888 1.000000 0.010000
C10 1 0.191880 0.773100 0.861137 1.000000 0.010000
C11 1 0.255426 0.749788 0.849662 1.000000 0.010000
C12 1 0.175195 0.869951 0.914288 1.000000 0.010000
C13 1 0.219916 0.939863 0.953936 1.000000 0.010000
C14 1 0.283067 0.913858 0.941723 1.000000 0.010000
C15 1 0.301271 0.818872 0.889780 1.000000 0.010000
C16 1 0.265058 0.497618 0.700797 1.000000 0.010000
C17 1 0.329788 0.500416 0.688142 1.000000 0.010000
C18 1 0.358926 0.412652 0.638759 1.000000 0.010000
C19 1 0.324317 0.321614 0.600914 1.000000 0.010000
C20 1 0.259957 0.318873 0.612435 1.000000 0.010000
C21 1 0.230459 0.405595 0.662401 1.000000 0.010000
O3 4 0.275754 0.658075 0.798033 1.000000 0.010000
O4 4 0.088342 0.712791 0.826004 1.000000 0.010000
END

6.3. Calculated low-energy dapsone/flavone structures

Table S16. Dapsone/flavone: Computationally generated low-energy PBE-MBD* cocrystal structures, with the experimental structures marked in bold (and red).

Structure	Space group	Cell parameters						Energy	Packing
		a / Å	*b* / Å	*c* / Å	α / °	β / °	γ / °	kJ mol^{-1}	Index
**1 (A_{CC})**	***$P2_1/c$***	**11.17**	**11.48**	**18.04**	**90.0**	**101.5**	**90.0**	**-685690.30**	**0.71**
2	*$P2_1/c$*	9.08	15.83	15.91	90.0	104.0	90.0	-685688.20	0.73
3	*$P2_1/c$*	5.70	11.85	32.93	90.0	94.4	90.0	-685683.58	0.73
4	*$P2_1/c$*	13.19	9.21	17.72	90.0	90.0	90.0	-685682.65	0.75
**5 (B_{CC})**	***Fdd*2**	**22.28**	**70.95**	**5.49**	**90.0**	**90.0**	**90.0**	**-685681.16**	**0.75**
6	*$P2_1/c$*	10.21	13.94	15.36	90.0	88.9	90.0	-685680.44	0.74
7	*$P2_1$*	6.00	19.32	9.27	90.0	99.1	90.0	-685677.70	0.76
8	*$P2_12_12_1$*	5.83	12.85	29.36	90.0	90.0	90.0	-685677.54	0.74
9	*$P2_1/c$*	17.25	14.83	8.58	90.0	99.0	90.0	-685677.19	0.74
10	*Pbca*	15.32	15.94	18.33	90.0	90.0	90.0	-685676.91	0.72
11	*$P2_12_12_1$*	5.66	11.49	34.21	90.0	90.0	90.0	-685676.52	0.73
12	*$P2_12_12_1$*	6.79	12.71	25.38	90.0	90.0	90.0	-685676.18	0.74
13	*$P2_1/c$*	20.56	12.98	7.91	90.0	92.7	90.0	-685675.15	0.77
14	*Cc*	10.74	10.74	19.02	49.1	49.1	46.2	-685674.53	0.73
15	*$P2_1/c$*	11.60	17.90	11.72	90.0	117.1	90.0	-685674.42	0.75
16	P-1	8.27	11.06	12.97	102.4	104.0	103.9	-685673.85	0.76
17	*$P2_1/c$*	10.21	14.71	14.41	90.0	92.7	90.0	-685673.29	0.75
18	*$P2_1/c$*	10.27	26.05	8.89	90.0	114.4	90.0	-685672.73	0.75
19	*P*-1	8.09	9.02	16.31	108.5	92.5	104.1	-685672.58	0.74
20	*P*-1	9.30	11.21	11.71	97.9	99.3	113.3	-685672.14	0.75
21	*Pbca*	18.15	9.05	26.34	90.0	90.0	90.0	-685672.08	0.75
22	*$P2_1/c$*	6.68	19.84	16.71	90.0	99.3	90.0	-685671.92	0.74
23	*$P2_1/c$*	15.41	8.44	17.62	90.0	106.2	90.0	-685671.91	0.74
24	*P*-1	8.64	9.24	14.43	94.4	98.6	105.4	-685670.75	0.75
25	*$P2_1/c$*	8.82	32.57	8.47	90.0	111.2	90.0	-685670.71	0.71
26	*$P2_1/c$*	17.66	7.73	16.85	90.0	109.2	90.0	-685670.00	0.74
27	*P*-1	10.06	10.62	11.77	63.2	74.1	84.9	-685669.68	0.75
28	*$P2_1/c$*	5.79	11.92	31.19	90.0	92.0	90.0	-685669.62	0.75
29	*$P2_1$*	7.38	16.45	9.08	90.0	91.2	90.0	-685669.59	0.74
30	*$P2_1/c$*	7.15	22.68	13.65	90.0	96.5	90.0	-685669.48	0.74
31	*C2/c*	17.22	17.22	30.75	32.4	32.4	30.2	-685668.90	0.72
32	*$P2_1/c$*	8.09	29.65	9.36	90.0	101.3	90.0	-685668.57	0.74
33	*P*-1	6.88	9.20	17.99	96.1	100.4	92.5	-685667.88	0.73
34	*$P2_1/c$*	6.88	42.34	7.75	90.0	100.0	90.0	-685667.87	0.72
35	*$P2_12_12_1$*	6.69	13.49	24.27	90.0	90.0	90.0	-685667.46	0.74
36	*P*-1	8.13	9.04	15.13	96.1	93.9	93.6	-685667.28	0.74
37	*P*-1	8.52	9.20	14.85	77.7	82.4	75.4	-685666.83	0.74
38	*$P2_1/c$*	6.83	13.82	23.23	90.0	98.2	90.0	-685666.41	0.74
39	*$P2_1/c$*	7.04	21.79	14.18	90.0	90.0	90.0	-685666.04	0.75
40	*$Pna2_1$*	32.73	5.72	11.73	90.0	90.0	90.0	-685665.66	0.74
41	*$P6_1$*	26.72	26.72	5.96	90.0	90.0	120.0	-685665.41	0.66
42	*P*-1	9.19	10.93	12.49	72.9	73.3	67.3	-685663.16	0.74
43	*$P2_1/c$*	21.05	12.81	7.96	90.0	91.0	90.0	-685661.56	0.76

6.4. Calculated low-energy sulfaguanidine/flavone structures

Table S17. Sulfaguanidine/flavone: Computationally generated low-energy PBE-MBD* cocrystal structures, with the experimental structures marked in bold (and red). **II**$_{CC}$, a Z'=2 structure was added to the list. The optimization of IICC used identical settings as those employed for generating the 1:1 cocrystals.

Structure	Space group	Cell parameters						Energy kJ mol^{-1}	Packing Index
		a / Å	*b* / Å	*c* / Å	α / °	β / °	γ / °		
**1 (II$_{CC}$)**	***P*2$_1$2$_1$2$_1$**	**6.98**	**19.66**	**29.36**	**90.0**	**90.0**	**90.0**	**-660195.72**	**0.68**
2	*P*2$_1$/c	11.77	7.00	24.08	90.0	79.9	90.0	-660195.11	0.75
3	*P*-1	10.70	12.12	13.27	77.4	117.2	56.8	-660194.11	0.73
4	*P*2$_1$2$_1$2$_1$	11.82	23.87	6.96	90.0	90.0	90.0	-660193.93	0.74
5	*P*2$_1$/*c*	7.16	14.50	22.65	90.0	56.3	90.0	-660193.25	0.75
6	*P*2$_1$/*c*	15.15	9.54	13.63	90.0	81.2	90.0	-660192.34	0.75
7	*P*2$_1$2$_1$2$_1$	34.62	5.63	9.84	90.0	90.0	90.0	-660192.15	0.76
8	*P*2$_1$2$_1$2$_1$	10.68	9.63	20.02	90.0	90.0	90.0	-660190.70	0.71
9	*P*-1	7.08	15.52	9.26	88.2	76.2	89.7	-660189.71	0.74
10	*P*-1	7.09	10.09	17.39	111.3	115.9	91.9	-660189.02	0.72
11	*P*2$_1$/*c*	10.69	10.41	18.00	90.0	88.0	90.0	-660188.67	0.73
12	*P*2$_1$/*c*	11.38	7.08	27.20	90.0	67.0	90.0	-660188.40	0.72
13	*P*2$_1$/*c*	8.22	24.23	10.75	90.0	73.9	90.0	-660188.35	0.71
14	*P*2$_1$/*c*	16.59	12.97	9.62	90.0	101.8	90.0	-660188.16	0.72
15	*P*2$_1$/*c*	14.52	19.58	7.06	90.0	84.3	90.0	-660186.95	0.73
16	*P*-1	10.02	9.81	14.61	70.2	119.1	74.0	-660186.62	0.74
17	*P*2$_1$/*c*	10.87	22.71	8.21	90.0	75.0	90.0	-660186.28	0.75
18	*P*-1	14.42	9.68	14.57	134.4	112.7	98.9	-660185.78	0.71
19	*P*2$_1$/*c*	9.87	29.70	7.01	90.0	82.9	90.0	-660185.38	0.72
20	*C*2/*c*	22.98	9.51	18.98	90.0	77.3	90.0	-660184.24	0.72
21	*P*2$_1$/*c*	14.21	11.65	12.89	90.0	73.2	90.0	-660183.34	0.72
22	*P*2$_1$2$_1$2$_1$	32.08	5.85	10.89	90.0	90.0	90.0	-660183.25	0.71
23	*P*-1	10.35	10.37	10.04	75.5	88.1	82.8	-660181.91	0.70
24	*P*2$_1$/*c*	16.61	7.08	17.26	90.0	86.0	90.0	-660180.62	0.72
25	*P*2$_1$2$_1$2$_1$	5.47	29.25	12.35	90.0	90.0	90.0	-660180.13	0.74
26	*P*2$_1$2$_1$2$_1$	5.13	36.68	10.48	90.0	90.0	90.0	-660177.59	0.74

6.5. Calculated low-energy sulfanilamide structures

Table S18. Sulfanilamide: Computationally generated low-energy PBE-MBD* structures, with the experimental structures marked in bold (and red).

Structure	Space group	Cell parameters						Energy	Packing
		a / Å	*b* / Å	*c* / Å	α / °	β / °	γ / °	kJ mol^{-1}	Index
**1 (α_{SA})**	***Pbca***	**18.35**	**14.42**	**5.50**	**90.0**	**90.0**	**90.0**	**-265643.07**	**0.75**
**2 (β_{SA})**	***$P2_1/c$***	**16.80**	**8.87**	**9.88**	**90.0**	**99.7**	**90.0**	**-265642.97**	**0.75**
3	$Pna2_1$	16.40	8.56	5.03	90.0	90.0	90.0	-265642.62	0.77
4	$P2_12_12_1$	8.58	5.92	15.56	90.0	90.0	90.0	-265641.82	0.68
5	$P2_1/c$	5.69	8.64	15.65	90.0	88.9	90.0	-265641.32	0.70
6	$P2_1/c$	10.11	15.39	9.82	90.0	81.2	90.0	-265641.23	0.71
7	$P2_1/c$	9.34	7.77	11.06	90.0	63.4	90.0	-265641.17	0.76
8	$P2_1/c$	7.58	15.46	6.46	90.0	88.5	90.0	-265640.97	0.71
9	*Pbca*	8.74	16.55	10.09	90.0	90.0	90.0	-265640.48	0.74
10	$P2_1$	5.51	18.16	7.19	90.0	90.1	90.0	-265640.33	0.75
11	*Pbca*	16.81	19.70	8.80	90.0	90.0	90.0	-265640.01	0.75
12	$P2_1/c$	14.91	17.40	5.57	90.0	81.9	90.0	-265639.92	0.76
13	*Pbcn*	9.47	16.17	10.03	90.0	90.0	90.0	-265639.84	0.70
14	*P*-1	15.43	7.60	6.45	91.5	89.0	89.1	-265639.83	0.71
**15 (δ_{SA})**	***Pbca***	**8.50**	**17.82**	**9.70**	**90.0**	**90.0**	**90.0**	**-265639.53**	**0.74**
16	$P2_1/c$	15.98	8.93	10.21	90.0	102.4	90.0	-265639.28	0.77
17	$P2_12_12_1$	15.70	9.98	4.77	90.0	90.0	90.0	-265638.70	0.72
18	$P2_1/c$	10.81	5.43	15.63	90.0	119.9	90.0	-265638.45	0.67
19	$P2_1/c$	9.14	10.57	9.23	90.0	120.4	90.0	-265638.25	0.71
20	$P2_12_12_1$	16.82	5.01	8.52	90.0	90.0	90.0	-265638.05	0.76
**21 (γ_{SA})**	***$P2_1/c$***	**9.32**	**12.82**	**7.59**	**90.0**	**126.7**	**90.0**	**-265638.05**	**0.74**
22	*Pbca*	15.95	12.02	8.04	90.0	90.0	90.0	-265638.00	0.71
23	*Pbca*	15.48	18.52	5.47	90.0	90.0	90.0	-265637.94	0.68
24	$Pca2_1$	11.48	15.73	8.69	90.0	90.0	90.0	-265637.89	0.69
25	$P2_1$	7.63	5.72	8.63	90.0	88.1	90.0	-265637.05	0.71
26	$P2_1/c$	17.53	8.91	9.97	90.0	75.2	90.0	-265636.80	0.72
27	$P2_1/c$	10.06	19.61	9.22	90.0	57.1	90.0	-265636.65	0.71
28	*Pbca*	9.91	33.84	8.82	90.0	90.0	90.0	-265636.50	0.73
29	$P2_1/c$	16.72	9.37	10.07	90.0	110.5	90.0	-265636.42	0.73
30	*P*-1	8.01	15.48	6.23	84.2	89.0	93.3	-265636.38	0.70
31	*Pbca*	18.39	10.35	7.55	90.0	90.0	90.0	-265636.24	0.75
32	$P2_12_12_1$	7.20	36.49	5.56	90.0	90.0	90.0	-265635.98	0.74
33	$P2_1/c$	6.41	15.59	15.32	90.0	88.5	90.0	-265635.93	0.70
34	$Pna2_1$	9.38	5.29	30.72	90.0	90.0	90.0	-265635.86	0.71
35	*Pbca*	6.88	15.11	14.01	90.0	90.0	90.0	-265635.33	0.74
36	*Pbca*	33.41	8.95	10.00	90.0	90.0	90.0	-265635.27	0.73
37	*Pbca*	14.75	14.39	6.83	90.0	90.0	90.0	-265635.12	0.74
38	$P2_12_12_1$	18.22	7.93	5.20	90.0	90.0	90.0	-265635.03	0.73
39	*Cc*	10.37	5.18	15.56	90.0	101.5	90.0	-265634.86	0.66
40	$Pna2_1$	9.81	13.79	11.48	90.0	90.0	90.0	-265634.80	0.70
41	$P2_1/c$	7.73	25.51	7.81	90.0	73.5	90.0	-265634.68	0.74
42	$P2_1/c$	19.61	9.28	9.13	90.0	63.6	90.0	-265634.62	0.73
43	$P2_1$	5.14	9.37	7.75	90.0	81.4	90.0	-265634.58	0.74
44	$P2_1/c$	6.82	7.53	14.63	90.0	74.1	90.0	-265634.56	0.75
45	$Pca2_1$	8.78	8.20	21.11	90.0	90.0	90.0	-265634.55	0.72
46	$P2_1/c$	17.43	8.92	9.61	90.0	83.0	90.0	-265634.45	0.73

Structure	Space group	Cell parameters						Energy	Packing
		a / Å	*b* / Å	*c* / Å	α / °	β / °	γ / °	kJ mol^{-1}	Index
47	$Pna2_1$	36.31	5.24	7.93	90.0	90.0	90.0	-265634.19	0.72
48	*Cc*	16.51	6.25	7.59	90.0	80.9	90.0	-265634.08	0.70
49	*R*3*c*	23.64	23.64	7.49	90.0	90.0	120.0	-265634.04	0.67
50	*P*-1	13.14	7.95	9.23	89.8	51.6	89.9	-265633.99	0.72
51	$P2_1/c$	5.05	9.02	17.70	90.0	67.3	90.0	-265633.88	0.73
52	$Pna2_1$	10.44	15.11	4.72	90.0	90.0	90.0	-265633.85	0.73
53	$P2_1$	7.90	5.26	9.12	90.0	89.9	90.0	-265633.76	0.73
54	$P2_1/c$	12.84	5.34	10.80	90.0	97.0	90.0	-265633.69	0.73
55	*Pc*	11.23	8.62	7.79	90.0	80.2	90.0	-265633.68	0.73
56	$P2_1/c$	12.92	7.01	18.62	90.0	122.3	90.0	-265633.61	0.76
57	*Pccn*	5.51	14.44	18.45	90.0	90.0	90.0	-265633.56	0.74
58	*Pbcn*	16.61	6.05	15.39	90.0	90.0	90.0	-265633.45	0.70
59	$P2_1/c$	13.93	9.37	11.35	90.0	87.3	90.0	-265633.41	0.73
60	$P2_12_12_1$	8.10	17.86	5.15	90.0	90.0	90.0	-265633.40	0.73
61	*Fdd*2	31.58	16.37	5.88	90.0	90.0	90.0	-265633.17	0.71
62	$Pca2_1$	14.32	9.66	5.46	90.0	90.0	90.0	-265633.16	0.72
63	*P*-1	7.85	9.23	12.14	118.7	90.0	90.0	-265633.11	0.71
64	$P2_1/c$	5.08	15.09	10.21	90.0	86.9	90.0	-265632.70	0.69
65	$P2_1/c$	9.18	5.20	15.91	90.0	89.5	90.0	-265632.47	0.72
66	*Fdd*2	19.42	28.52	5.16	90.0	90.0	90.0	-265632.43	0.76
67	$Pca2_1$	10.80	7.98	9.03	90.0	90.0	90.0	-265632.41	0.70
68	*P*-1	7.72	7.79	6.53	90.9	80.4	90.1	-265632.41	0.71
69	*Fdd*2	28.56	19.34	5.18	90.0	90.0	90.0	-265632.36	0.76
70	$Pca2_1$	18.44	7.84	10.66	90.0	90.0	90.0	-265632.34	0.70
71	$P2_1/c$	7.90	5.20	40.07	90.0	66.7	90.0	-265632.27	0.72
72	$P2_1/c$	12.17	5.21	24.30	90.0	98.0	90.0	-265632.11	0.71
73	$P2_1/c$	12.22	5.20	12.16	90.0	81.3	90.0	-265632.06	0.71
74	*P*-1	9.20	7.93	14.46	122.7	113.9	90.4	-265631.97	0.70
75	$P2_1/c$	9.20	5.21	36.67	90.0	120.3	90.0	-265631.92	0.72
76	$P2_1/c$	5.24	15.25	11.14	90.0	123.4	90.0	-265631.71	0.73
77	$P2_1/c$	9.27	26.68	7.63	90.0	53.7	90.0	-265631.36	0.71
78	*P*-1	6.81	7.21	15.36	73.1	90.5	90.1	-265630.03	0.76
79	$P2_1/c$	10.18	4.87	16.31	90.0	88.1	90.0	-265630.00	0.67
80	$P2_1/c$	8.12	4.91	19.77	90.0	88.0	90.0	-265629.87	0.69
81	$P2_1/c$	4.92	10.83	15.04	90.0	74.6	90.0	-265629.67	0.70
82	$P2_1/c$	21.97	8.19	9.42	90.0	116.5	90.0	-265629.44	0.71
83	$P4_12_12$	7.99	7.99	23.14	90.0	90.0	90.0	-265629.34	0.74

6.6. Calculated low-energy dapsone structures (recalculated)

Table S19. Dapsone: Computationally generated low-energy PBE-MBD* structures, with the experimental structures marked in bold (and red).

Structure	Space group	Cell parameters						Energy	Packing
		a / Å	*b* / Å	*c* / Å	α / °	β / °	γ / °	kJ mol^{-1}	Index
**1 (V_{DDS})**	***$P2_1/c$***	**29.11**	**5.64**	**29.97**	**90.0**	**115.6**	**90.0**	**-361580.93**	**0.75**
**2 (III_{DDS})**	***$P2_12_12_1$***	**5.46**	**7.93**	**25.74**	**90.0**	**90.0**	**90.0**	**-361580.88**	**0.75**
**3**	***$Pca2_1$***	**15.44**	**5.59**	**25.95**	**90.0**	**90.0**	**90.0**	**-361580.82**	**0.74**
4	*$Pna2_1$*	5.55	26.51	7.82	90.0	90.0	90.0	-361579.30	0.72
**5 ($dehy_{DDS}$)**	***$C2/c$***	**24.62**	**24.62**	**12.81**	**92.2**	**92.2**	**26.4**	**-361578.16**	**0.72**
6	*$Pca2_1$*	15.34	5.75	25.72	90.0	90.0	90.0	-361577.89	0.73
7	*Pbca*	15.88	15.83	18.81	90.0	90.0	90.0	-361576.84	0.70
**8 (I_{DDS})**	***$P2_1/c$***	**18.63**	**8.18**	**16.42**	**90.0**	**115.2**	**90.0**	**-361576.26**	**0.73**
**9 (II_{DDS})**	***$P2_12_12_1$***	**5.62**	**7.85**	**24.90**	**90.0**	**90.0**	**90.0**	**-361576.18**	**0.76**
10	*$P2_1$*	5.57	7.94	25.44	90.0	90.6	90.0	-361575.83	0.74
11	*$P2_1$*	5.53	7.81	26.65	90.0	90.0	90.0	-361575.36	0.72
12	*$Pna2_1$*	14.09	26.70	5.93	90.0	90.0	90.0	-361574.99	0.75
13	*$P2_1/c$*	14.56	5.58	15.27	90.0	115.9	90.0	-361574.62	0.74
14	*$P2_1/c$*	26.52	8.09	10.87	90.0	101.0	90.0	-361574.51	0.73
15	*$P2_1/c$*	26.53	8.10	10.70	90.0	100.8	90.0	-361574.27	0.74
16	*$Pna2_1$*	14.95	5.58	26.06	90.0	90.0	90.0	-361574.21	0.77
17	*Pbca*	5.71	15.18	26.28	90.0	90.0	90.0	-361574.18	0.73
**18 (IV_{DDS})**	***$Pca2_1$***	**16.16**	**8.23**	**17.45**	**90.0**	**90.0**	**90.0**	**-361574.16**	**0.72**
19 (DCM $desol_{DDS}$)	*$P2_1/c$*	8.42	16.06	18.69	90.0	98.9	90.0	-361573.74	0.66
20	*$P2_1/c$*	26.76	5.63	14.96	90.0	104.3	90.0	-361573.70	0.76
21	*$P2_1/c$*	5.77	27.79	13.97	90.0	93.7	90.0	-361572.83	0.75
22	*$P2_1/c$*	5.78	7.85	24.61	90.0	89.1	90.0	-361572.79	0.74
23	*$Pca2_1$*	28.84	5.67	13.74	90.0	90.0	90.0	-361572.72	0.74
24	*$P2_1/c$*	5.82	29.41	13.08	90.0	96.0	90.0	-361572.29	0.75
25	*$P2_1/c$*	26.50	5.68	15.11	90.0	104.9	90.0	-361572.12	0.76
26	*$P2_1/c$*	26.38	5.81	15.24	90.0	106.5	90.0	-361571.93	0.74
27	*$P2_1/c$*	8.54	15.01	18.41	90.0	101.8	90.0	-361571.83	0.72
28	*$Pna2_1$*	15.38	5.82	25.09	90.0	90.0	90.0	-361571.80	0.74
29	*$P2_1/c$*	8.00	13.25	10.21	90.0	92.6	90.0	-361571.69	0.77
30	*$P2_1/c$*	25.54	5.70	15.20	90.0	91.4	90.0	-361571.39	0.75
31	*$Pca2_1$*	5.73	26.30	14.97	90.0	90.0	90.0	-361571.16	0.74
32	$P2_12_12_1$	5.82	12.99	29.36	90.0	90.0	90.0	-361571.15	0.75
33	*$P2_1/c$*	11.57	12.64	7.64	90.0	100.9	90.0	-361571.14	0.76
34	*$Pca2_1$*	7.89	11.78	23.90	90.0	90.0	90.0	-361570.95	0.75
35	*$Pna2_1$*	15.16	5.83	25.32	90.0	90.0	90.0	-361570.91	0.75
36	*$P2_1$*	5.60	26.06	8.15	90.0	106.8	90.0	-361570.80	0.74
37	*$P2_1/c$*	11.52	12.58	7.68	90.0	100.2	90.0	-361570.67	0.76
38	*Cc*	14.31	14.31	27.49	33.0	33.0	22.5	-361570.66	0.75
39	$P2_12_12_1$	5.76	13.13	29.26	90.0	90.0	90.0	-361570.63	0.76
40	*$P2_1/c$*	26.14	5.65	15.02	90.0	93.2	90.0	-361570.63	0.75
41	*Cc*	14.05	14.05	27.15	33.7	33.7	23.5	-361570.33	0.74
42	*$P2_1/c$*	11.48	12.65	7.65	90.0	99.9	90.0	-361570.08	0.76
43	*$P2_1/c$*	15.48	7.90	18.38	90.0	97.7	90.0	-361569.44	0.75

6.7. Calculated low-energy flavone structures

Table S20. Flavone: Computationally generated low-energy PBE-MBD* structures, with the experimental structures marked in bold (and red).

Structure	Space group	Cell parameters						Energy	Packing
		a / Å	*b* / Å	*c* / Å	α / °	β / °	γ / °	kJ mol^{-1}	Index
**1 (I_{FL})**	***P*2₁2₁2₁**	**8.62**	**12.83**	**18.82**	**90.0**	**90.0**	**90.0**	**-324098.70**	**0.76**
**2 (II_{FL})**	***P*2₁/*c***	**22.01**	**4.84**	**22.24**	**90.0**	**117.9**	**90.0**	**-324098.24**	**0.75**
3	*Pna*2₁	11.36	19.04	4.80	90.0	90.0	90.0	-324097.82	0.76
4	*P*2₁/*c*	9.36	10.75	10.57	90.0	100.8	90.0	-324097.49	0.76
5	*Pc*	3.83	24.24	11.03	90.0	89.9	90.0	-324097.47	0.77
6	*P*2₁/*c*	22.35	3.91	23.19	90.0	92.9	90.0	-324096.72	0.78
7	*P*2₁/*c*	11.84	3.93	21.72	90.0	90.9	90.0	-324096.63	0.78
8	*P*2₁/*c*	3.87	23.48	11.42	90.0	100.4	90.0	-324096.63	0.77
9	*P*-1	3.90	11.39	22.97	90.3	90.0	86.4	-324096.59	0.77
10	*P*-1	3.85	21.39	26.71	112.7	90.2	90.4	-324096.43	0.78
11	*P*2₁/*c*	24.21	3.86	21.73	90.0	91.9	90.0	-324096.12	0.78
12	*P*2₁/*c*	4.06	19.85	12.77	90.0	98.4	90.0	-324096.07	0.77
13	*Pc*	3.84	22.58	23.51	90.0	90.4	90.0	-324095.82	0.78
14	*P*1	3.88	21.26	24.74	90.1	89.9	90.2	-324095.75	0.78
15	*P*2₁/*c*	5.25	8.46	22.77	90.0	93.7	90.0	-324095.58	0.78
16	*P*2₁/*c*	13.00	4.17	19.14	90.0	94.9	90.0	-324095.49	0.76
17	*P*2₁/*c*	3.89	46.15	11.35	90.0	93.4	90.0	-324095.34	0.78
18	*P*2/*c*	15.08	3.96	17.64	90.0	99.3	90.0	-324095.30	0.76
19	*Pbca*	7.58	20.99	25.79	90.0	90.0	90.0	-324095.30	0.77
20	*Pna*2₁	11.37	45.10	3.93	90.0	90.0	90.0	-324095.23	0.78
21	*Pna*2₁	41.38	12.43	3.95	90.0	90.0	90.0	-324095.04	0.78
22	*P*2₁/*c*	11.75	8.37	21.15	90.0	98.7	90.0	-324095.00	0.77
23	*P*2₁/*c*	20.53	3.89	25.29	90.0	93.5	90.0	-324094.98	0.78
24	*P*-1	4.90	12.51	18.04	70.6	86.9	80.1	-324094.92	0.77
25	*C*2/*c*	12.08	12.08	17.71	90.2	90.2	23.6	-324094.91	0.77
26	*P*2₁/*c*	12.94	4.14	19.44	90.0	93.7	90.0	-324094.87	0.76
27	*P*2₁/*c*	3.86	48.09	10.94	90.0	90.9	90.0	-324094.85	0.78
28	*P*2₁/*c*	12.93	3.88	20.21	90.0	93.1	90.0	-324094.82	0.78
29	*P*2₁/*c*	3.86	48.86	10.89	90.0	97.6	90.0	-324094.72	0.78
30	*P*1	3.93	11.44	22.76	90.5	94.6	94.2	-324094.68	0.78
31	*P*2₁/*c*	24.13	4.02	24.37	90.0	118.6	90.0	-324094.61	0.76
32	*P*-1	8.28	14.77	17.51	90.3	83.7	89.6	-324094.45	0.74
33	*Pna*2₁	42.66	12.25	3.90	90.0	90.0	90.0	-324094.43	0.78
34	*Pna*2₁	11.10	47.89	3.85	90.0	90.0	90.0	-324094.40	0.77
35	*Pna*2₁	25.85	4.02	19.87	90.0	90.0	90.0	-324094.25	0.77
36	*P*2₁/*c*	22.93	4.17	24.49	90.0	117.7	90.0	-324094.25	0.76
37	*P*-1	7.13	8.40	9.10	97.1	105.0	93.9	-324094.18	0.76
38	*P*2₁/*c*	11.95	4.21	20.77	90.0	104.2	90.0	-324094.10	0.78
39	*P*2₁/*c*	16.64	5.00	25.53	90.0	102.8	90.0	-324094.10	0.76
40	*P*2₁/*c*	15.79	3.88	16.63	90.0	96.7	90.0	-324094.08	0.78
41	*P*-1	6.93	8.08	20.18	80.4	84.9	68.8	-324094.06	0.76
42	*P*2₁/*c*	16.00	7.58	17.44	90.0	100.6	90.0	-324094.02	0.76
43	*P*2₁/*c*	11.57	3.89	44.64	90.0	91.4	90.0	-324093.99	0.79
44	*P*2₁/*c*	12.40	4.21	40.27	90.0	90.2	90.0	-324093.96	0.75
45	*P*2₁/*c*	22.49	4.15	22.17	90.0	96.9	90.0	-324093.88	0.77
46	*P*2₁/*c*	12.59	3.91	41.25	90.0	96.9	90.0	-324093.71	0.78

Structure	Space group	Cell parameters						Energy	Packing
		a / Å	*b* / Å	*c* / Å	α / °	β / °	γ / °	kJ mol^{-1}	Index
47	$P2_1/c$	10.80	3.85	49.26	90.0	91.3	90.0	-324093.57	0.77
48	$P2_1/c$	10.88	24.94	7.65	90.0	94.0	90.0	-324093.46	0.77
49	*P*-1	4.18	13.01	19.12	90.4	89.5	83.8	-324093.45	0.76
50	$P2_1/c$	21.14	3.89	24.55	90.0	94.2	90.0	-324093.43	0.79
51	*Pc*	19.95	4.03	25.65	90.0	90.7	90.0	-324093.42	0.77
52	*C*2/*c*	20.92	20.92	24.56	90.2	90.2	11.2	-324093.29	0.74
53	*Cc*	17.84	17.84	12.29	90.7	90.7	164.4	-324093.29	0.76
54	*P*-1	4.09	23.23	23.71	112.1	95.7	95.6	-324093.22	0.77
55	$P2_1/c$	10.63	3.89	24.38	90.0	96.0	90.0	-324093.19	0.79
56	$P2_1/c$	12.40	3.92	41.98	90.0	93.2	90.0	-324093.16	0.78
57	$Pca2_1$	49.29	3.85	10.83	90.0	90.0	90.0	-324093.13	0.77
58	$P2_1/c$	12.66	9.66	17.78	90.0	101.6	90.0	-324093.13	0.74
59	$P2_1/c$	17.67	4.36	26.65	90.0	99.7	90.0	-324093.06	0.78
60	$Pna2_1$	10.42	48.26	4.00	90.0	90.0	90.0	-324092.85	0.79
61	*P*-1	3.81	12.47	22.22	89.7	80.5	88.6	-324092.32	0.76
62	$P2_1/c$	11.79	11.75	15.59	90.0	104.8	90.0	-324092.28	0.76
63	$P2_1/c$	4.33	37.84	12.93	90.0	98.8	90.0	-324091.82	0.76
64	*P*1	4.13	12.76	20.13	88.0	83.6	77.3	-324091.67	0.77
65	*P*-1	5.83	8.97	10.91	109.5	91.3	95.9	-324091.66	0.73
66	*P*-1	7.59	11.64	24.07	89.5	86.3	89.9	-324090.36	0.74
67	*P*-1	4.53	14.44	17.85	91.7	91.1	63.8	-324089.25	0.75

6.8. Crystal packing similarity of predicted cocrystal structures

The packing similarity dendrograms were generated with the CCDC's 'packing_similarity_dendrogram' python script and standard settings available on GitHub[3] according to Childs *et al*[4].

6.8.1. Sulfanilamide/flavone packing similarity dendrogram

The different crystal packing motifs/arrangements of sulfanilamide in the computed sulfanilamide/flavone cocrystals were compared. The numbers represent the stability order (ID) of the structures. For the structures marked in red, the cocrystal lattice energy is lower than sum of the single components lattice energies. Among the 7 most stable structures, only structure 3 and 5 form identical packings, the others feature distinct packing motifs, which is also discussed in section 3.3 of the manuscript.

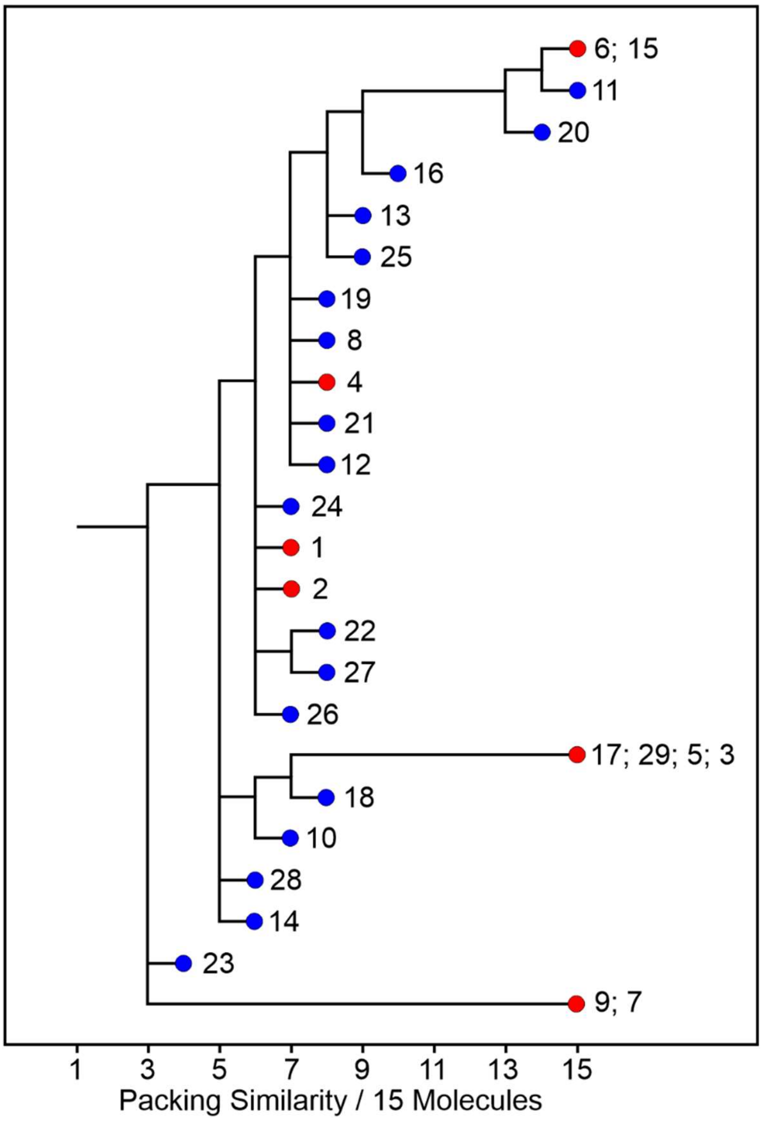

Figure S9 Packing similarity dendrogram of sulfanilamide in the sulfanilamide/flavone cocrystal structures omitting flavone molecules. Red dots: thermodynamically feasible cocrystal structures; The numbers represent the rank (ID) of the structures.

6.8.2. Dapsone/flavone Dendrogram

The packing similarity dendrogram of the CSP generated dapsone/flavone cocrystals was generated. The numbers represent the stability order (ID) of the computed structures. For the structures marked in red, the cocrystal lattice energy is lower than sum of the single components lattice energies. Among the 7 most stable structures, distinct packing motifs are observed.

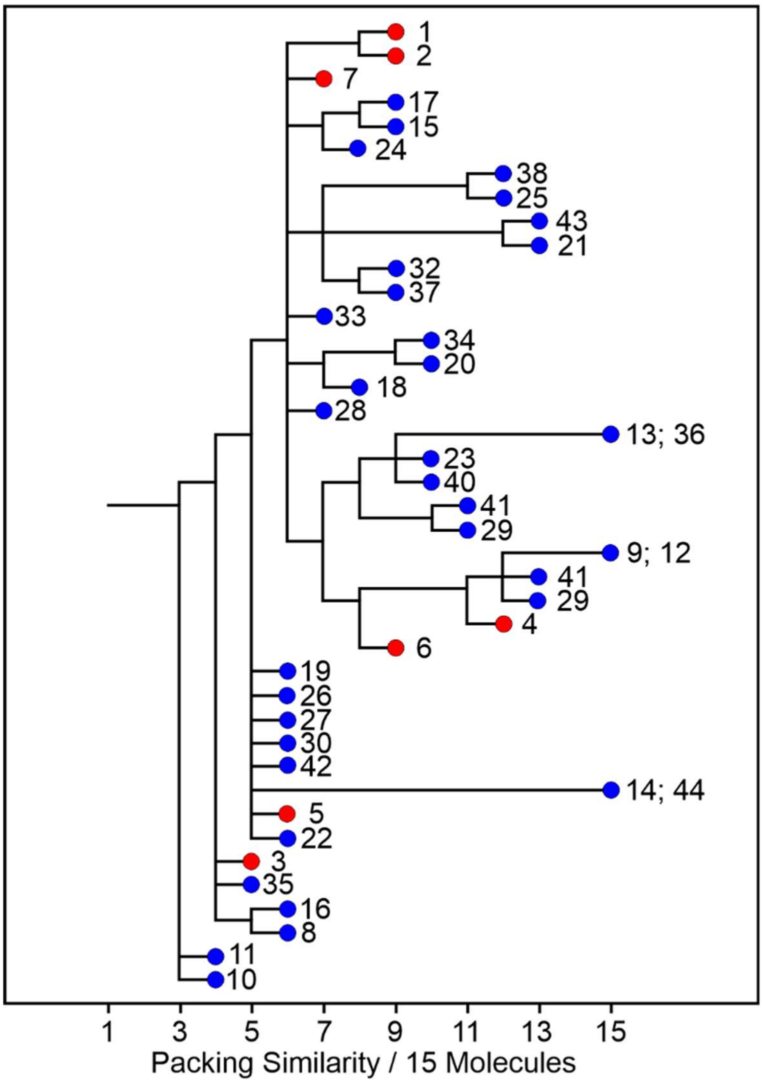

Figure S10 Packing similarity dendrogram of dapsone in the dapsone/flavone cocrystal structures omitting flavone molecules. Red dots: thermodynamically feasible cocrystal structures; The numbers represent the rank (ID) of the structures.

6.8.3. Sulfaguanidine/flavone Dendrogram

Among the 7 most stable structures, all structures are unique, although there are packing motifs in common with the sulfaguanidine (single-component) structures. For more details, please refer to section 3.3 of the manuscript.

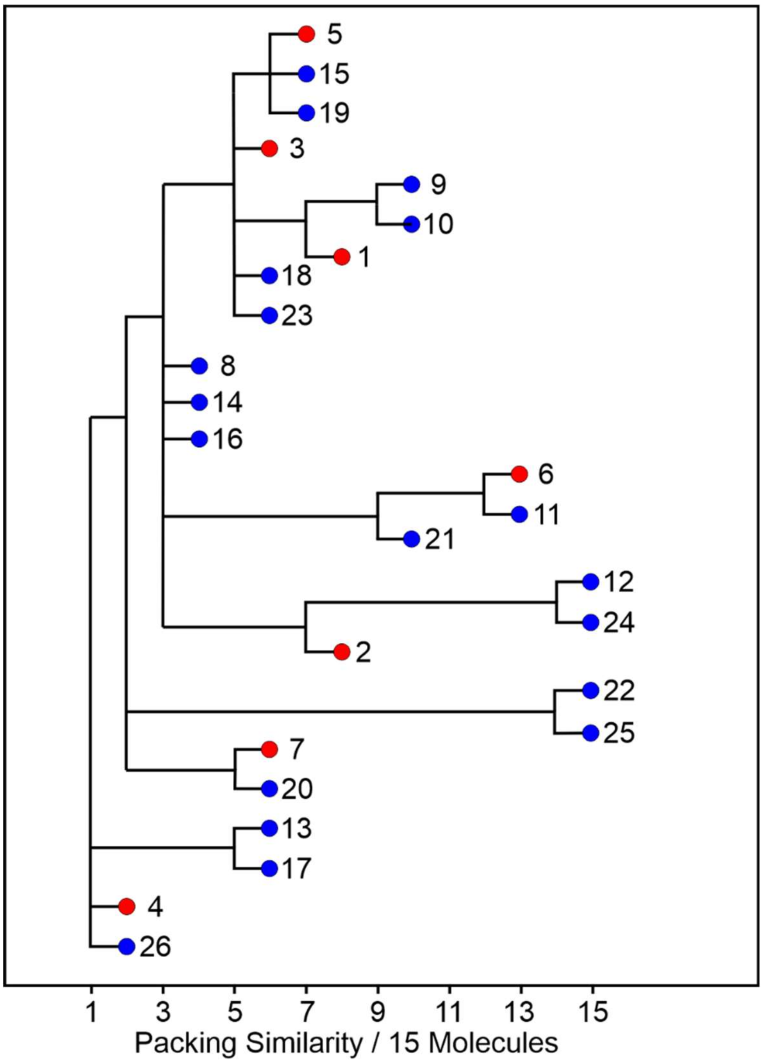

Figure S11 Packing similarity dendrogram of sulfaguanidine in the sulfaguanidine/flavone cocrystal structures omitting flavone molecules. Red dots: thermodynamically feasible cocrystal structures; The numbers represent the rank (ID) of the structures.

B) SOLID-STATE FORM CHARACTERIZATION

7. Structure solution form PXRD data

The crystal structures of **II$_{FL}$**, **II$_{CC}$**, and **E$_{CC}$** were successfully solved from PXRD data.

The PXRD patterns were recorded using an X'Pert PRO diffractometer (PANalytical, Almelo, NL) in transmission geometry, with a Cu-K$_{\alpha1,2}$ radiation source, PIXcel1D detector, 40 kV/40 mA, and a step size of 2θ = 0.007° with 800 s (**E$_{CC}$**) or 1600 (**FL$_{II}$** and **II$_{CC}$**) in the 2θ range between 2° and 70°.

The diffraction patterns were indexed using 20-24 peaks with DICVOL, and the space groups determined based on a statistical assessment of systematic absences,[5] as implemented in the DASH structure solution package.[6] From the cell volume, it was determined that there are two FL molecules in the **II$_{FL}$** (Z'=2, $P2_1/n$), two SG and two FL in the **II$_{CC}$** (Z'=2, $P2_12_12_1$), and each one DDS, FL, and *t*-BuOH molecule in the **E$_{CC}$** (Z'=1, $P2_1/c$) asymmetric unit. The data were background subtracted, and Pawley refinement[7] was used to extract the intensities and their correlations. Simulated annealing was used to optimize the models against the diffraction data set in direct space. The internal coordinate (*Z*-matrix) descriptions were derived from the PBE0/6-31G(d,p) gas phase global conformational minima, with O–H and N–H distances normalized to 0.9 Å and C–H distances to 0.95 Å. Each of the structures was solved using 100 simulated annealing runs of 2 x 10^8 moves per run in DASH. Each DDS or SG molecule was allowed 6 external and 2 internal degrees of freedom, FL 6 external and 1 internal degrees, and *t*-BuOH only 6 external degrees of freedom. The best solutions were then subjected to PBE-MBD* optimizations (CASTEP). All chosen structure solutions had refined to a χ^2 ratio of < 4 (profile χ^2/ pawley χ^2). The optimized structures, with O–H/N–H distances normalized to 0.9 Å and C–H distances to 0.95 Å were then used as the starting point for rigid-body Rietveld refinements[8] using TOPAS V7.12[9]. The background was modelled with Chebyshev polynomials. For more details please refer to Figures S12-S14 and Table S21.

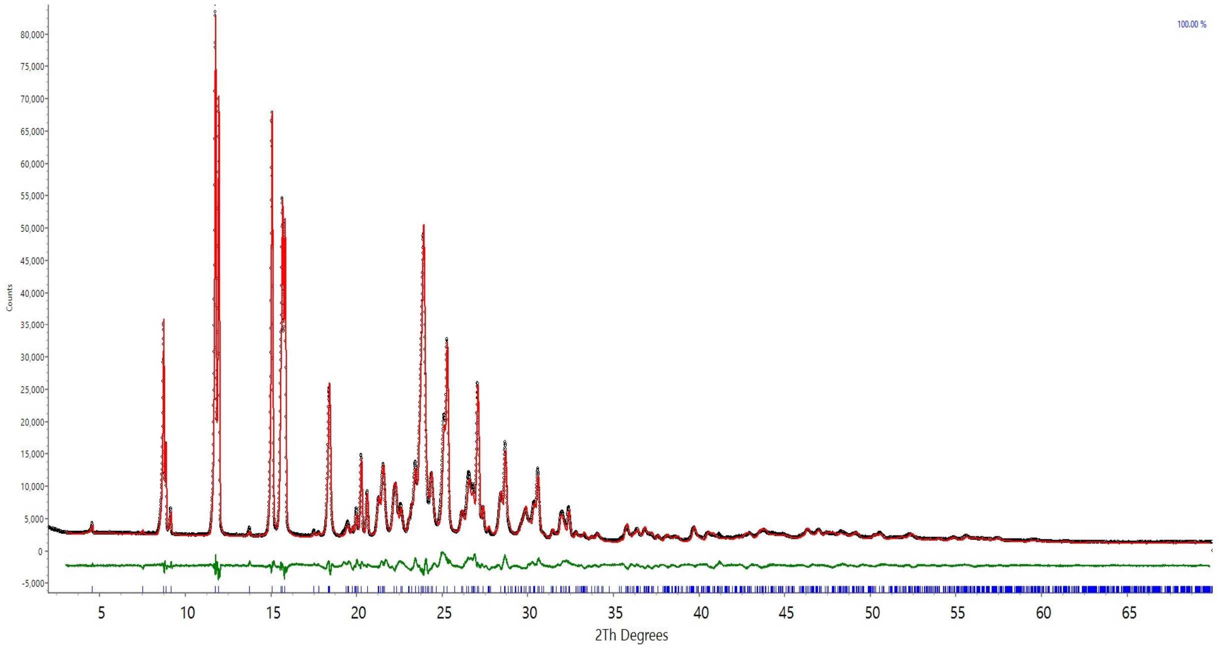

Figure S12. Powder X-ray diffraction pattern and Rietveld fit (rigid body) of FLAVONE II (**II$_{FL}$**): Observed (black points), calculated (red line), and difference profiles (green line). Blue tick marks denote the peak positions.

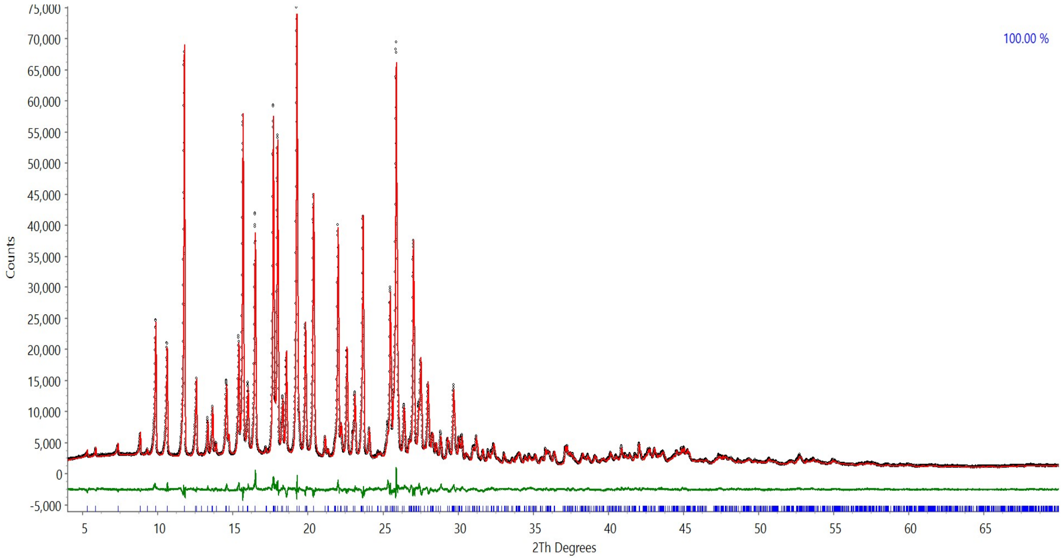

Figure S13. Powder X-ray diffraction pattern and Rietveld fit (rigid body) of SG/FL cocrystal II ($\mathbf{II_{CC}}$): Observed (black points), calculated (red line), and difference profiles (green line). Blue tick marks denote the peak positions.

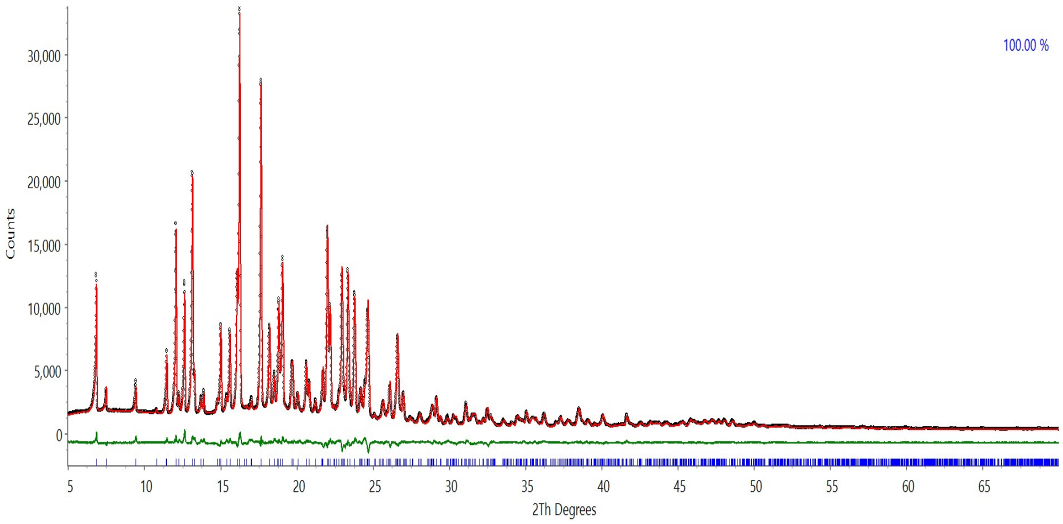

Figure S14. Powder X-ray diffraction pattern and Rietveld fit (rigid body) of DDS/FL/*t*-butanol cocrystal solvate ($\mathbf{E_{CC}}$): Observed (black points), calculated (red line), and difference profiles (green line). Blue tick marks denote the peak positions.

Table S21. Experimental details for the **II$_{FL}$**, **II$_{CC}$**, and **E$_{CC}$** structures.

Crystal data	**II$_{FL}$**	**II$_{CC}$**	**E$_{CC}$**
Chemical formula	$C_{15}H_{10}O_2$	$C_{15}H_{10}O_2$· $C_7H_{10}N_4O_2S$	$C_{12}H_{12}N_2O_2S$· $C_{15}H_{10}O_2$·$C_4H_{10}O$
Molar mass	222.23	436.48	544.65
Crystal system	Monoclinic	Orthorhombic	Monoclinic
Space group	$P2_1/n$	$P2_12_12_1$	$P2_1/c$
a / Å	22.4317(5)	7.03362(4)	8.24354(8)
b / Å	4.9684(1)	19.9909(2)	23.5981(3)
c / Å	22.7708(5)	30.0549(2)	15.5166(1)
α / °	90	90	90
β / °	117.317(2)	90	95.977(2)
γ / °	90	90	90
Volume / Å^3	2254.79(9)	4225.97(5)	3002.06 (6)
Z	8	8	4
Z'	2	2	1
Radiation type		Cu $K\alpha_{1.2}$	
μ / mm^{-1}	0.70	1.68	1.29
Data collection			
Diffractometer		Panalytical X'Pert PRO	
Temp. / K		298	
Data collection mode		Transmission	
2θ values / °		$2\theta_{min}$ = 2.001<br>$2\theta_{max}$ = 69.985<br>$2\theta_{step}$ = 0.007	
Refinement			
Parameters	41<br>Profile: 20<br>Cell: 4<br>Scale: 1<br>U_{iso}: 1<br>Preferred orientation: 15<br>Positions: 12	62<br>Profile: 20<br>Cell: 3<br>Scale: 1<br>U_{iso}: 1<br>Preferred orientation: 13<br>Positions: 24	44<br>Profile: 20<br>Cell: 4<br>Scale: 1<br>U_{iso}: 1<br>Preferred orientation: 0<br>Positions: 18
R_p	4.55	4.50	2.77
R_{wp}	6.04	5.75	3.66
R_{exp}	2.53	3.16	2.55
R_{Bragg}	2.76	2.97	2.36
χ^2	3.57	1.82	2.07

8. Flavone polymorphs

8.1. Preparation of FL_{II}

Flavone form II could be prepared by solvent evaporation experiments from acetone, ethanol, 1-propanol, 2-propanol, *t*-butanol, diethyl ether, 1-butanol, acetonitrile, chloroform, cyclohexane, dichloromethane, dimethyl sulfoxide, ethyl acetate, methanol, methyl ethyl ketone, methyl isobutyl ketone, methyl-tert-butyl ether, nitromethane, tetrahydrofuran, toluene, butyl acetate, diisopropyl ether, and *n*-heptane.

8.2. Powder X-ray diffraction

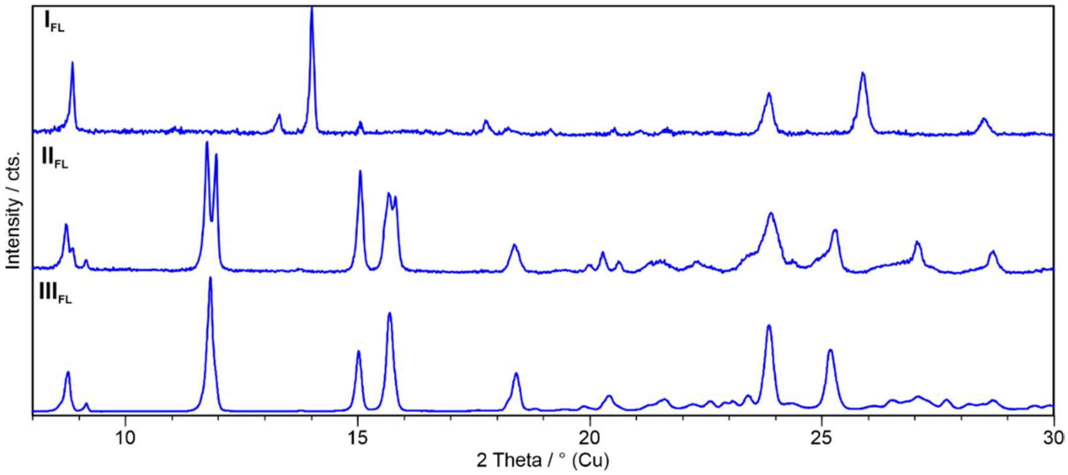

Figure S15. PXRD patterns of the flavone polymorphs.

8.3. IR Spectroscopy

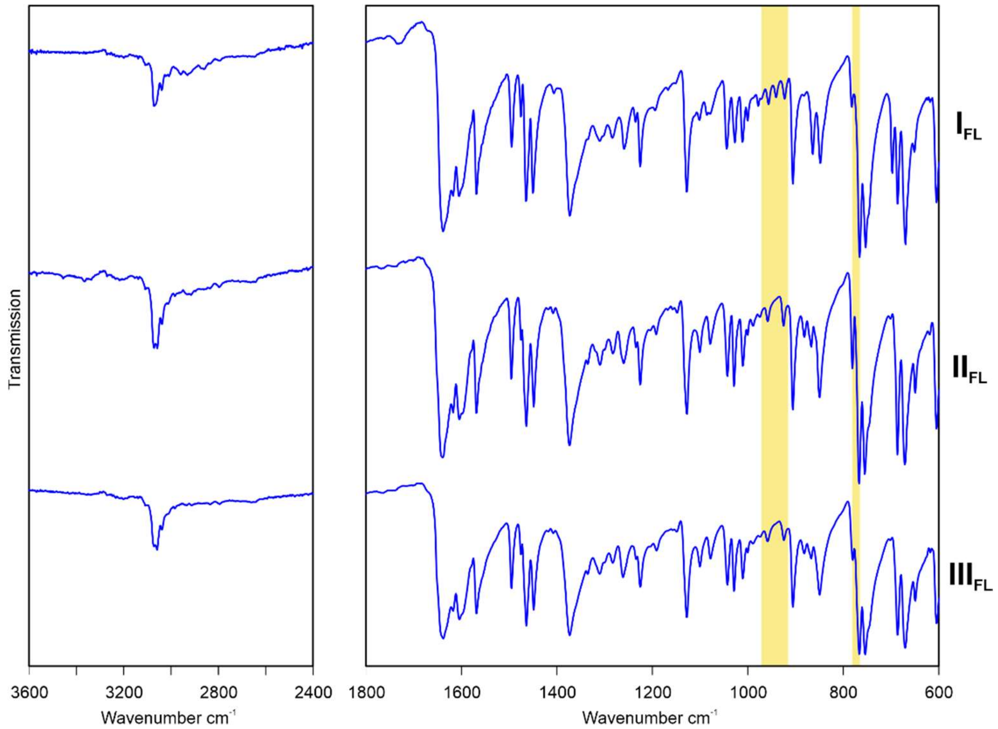

Figure S16. IR spectroscopy of the flavone polymorphs. Key differences are highlighted in yellow. II_{FL} and III_{FL} show very similar IR-spectra.

8.4. Thermal analysis: Differential scanning calorimetry and hot-stage microscopy

Figure S17a displays the DSC data of **II$_{FL}$**, which exhibits a melting point at 96.5 ± 0.1 °C along with a heat of fusion of 21.1 ± 0.2 kJ mol^{-1}. A negligible mass loss of less than 0.1% was observed in the temperature range of 25 – 105 °C, while employing a heating rate of 10 °C min^{-1} in TGA measurements. Upon cooling the melt, another polymorphic form, **III$_{FL}$**, recrystallizes at approximately 78 °C. **III$_{FL}$** often recrystallizes concomitantly with **II$_{FL}$**. The melting point of **III$_{FL}$** was determined at 95.6 ± 0.2 °C, with a heat of fusion of 19.6 ± 0.2 kJ mol^{-1}. Thus, based on the heat of fusion rule[10, 11] the two polymorphs are monotropically related.

Hot-stage microscopic investigations have revealed that when a **III$_{FL}$** sample containing **II$_{FL}$** is heated, the transition from **III$_{FL}$** to **II$_{FL}$** can be monitored (Figure S17b). This transformation process occurs over a wide temperature range. Phase-pure **III$_{FL}$** samples eventually transform into **FL$_{II}$** as well, although this transformation takes a longer time, often days at RT.

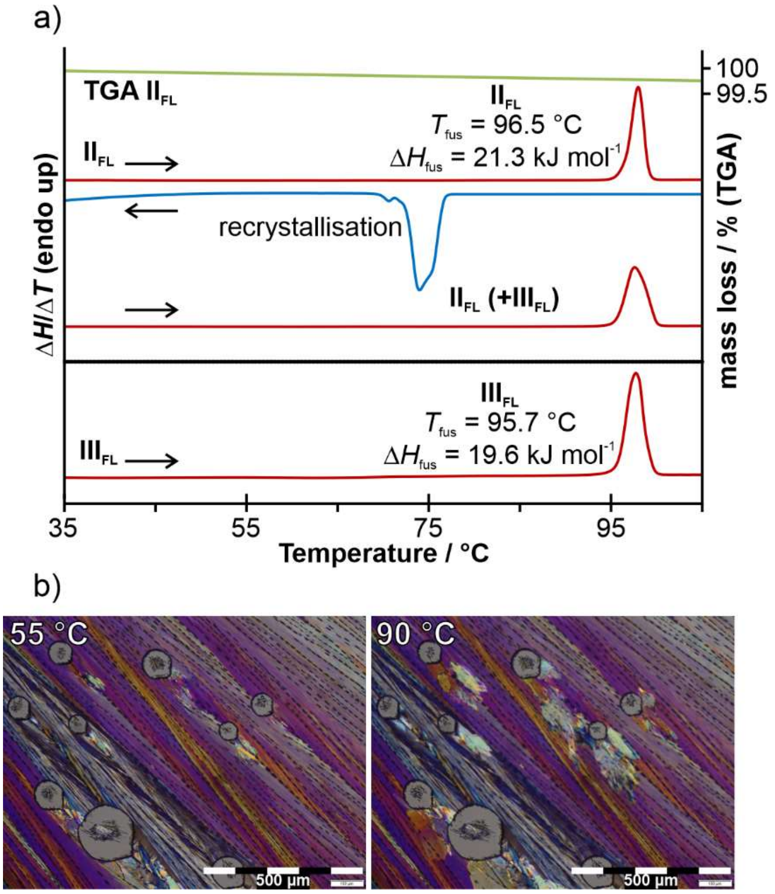

Figure S17 – a) DSC and TGA thermograms of flavone form II and III with a heating rate of 10 °C min^{-1} b) Hot stage microscopy of flavone form III with seed crystals form II growing at about 55 °C.

8.5. Pawley fit flavone Form III

The diffraction pattern of **III$_{FL}$** could be indexed to monoclinic unit cells (DICVOL). Pawley refinement in different space groups was then performed using TOPAS V7.12[9] (Table S23). The best match was seen for a cell very closely related to the **II$_{FL}$** lattice parameters and space group $P2_1$ (**III$_{FL}$**) instead of $P2_1/n$ (**II$_{FL}$**). The proposed **III$_{FL}$** lattice parameters, together with the similarity of the PXRD data and IR spectra suggest a close resemblance of the two polymorphs.

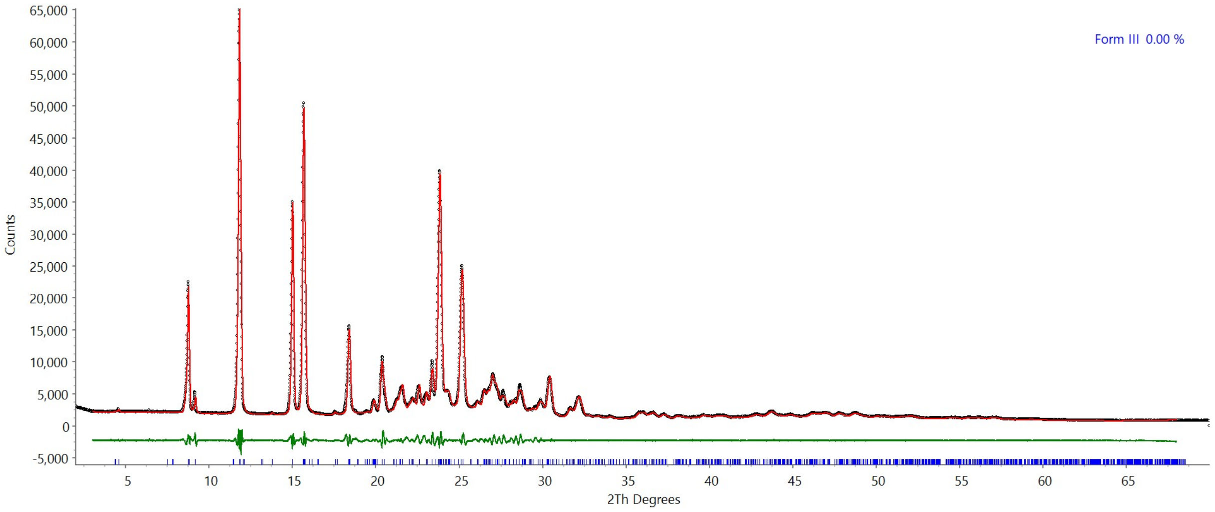

Figure S18. Powder X-ray diffraction pattern and Pawley fit (rigid body) of FLAVONE III (**III$_{FL}$**): Observed (black points), calculated (red line), and difference profiles (green line). Blue tick marks denote the peak positions.

Table S22. Potentail III$_{FL}$ lattice parameters. In green: all peak positions are accounted for with the model, in red: one peak positon not accounted for (wrong space group).

Space group	Z'	a / Å	b / Å	c / Å	b / °	V / Å^3	R_p	R_{wp}	R_{exp}	χ^2
Pn	4	22.6939(32)	4.9780(5)	22.5400(34)	117.025(4)	2268.29(52)	3.48	5.22	1.78	8.58
$P2_1$	4	22.6575(28)	4.9739(5)	22.5023(30)	116.992(4)	2259.69(47)	3.03	4.41	1.77	6.22
$P2_1/n$	2	22.678(32)	4.9772(5)	22.5229(34)	117.009(4)	2264.98(52)	3.50	5.26	1.79	8.63
Pc	4	23.2170(36)	11.7792(8)	9.5581(19)	123.960(12)	2168.07(65)	4.72	6.72	1.80	14.03
$P2_1$	4	23.2161(26)	11.7737(9)	9.5509(15)	123.966(8)	2165.18(48)	4.50	6.46	1.79	13.06
$P2_1/c$	2	23.2300(25)	11.7786(9)	9.5451(13)	123.998(7)	2165.23(45)	4.78	6.88	1.80	14.70
Pc	2	22.9137(17)	11.7853(7)	5.9144(8)	122.815(6)	1342.28(25)	6.16	9.76	1.83	28.46
$P2_1$	2	22.9247(23)	11.7799(11)	5.9106(21)	122.850(8)	1340.92(53)	6.40	10.50	7.83	33.06

9. Sulfaguanidine/flavone cocrystals

9.1. Powder X-ray diffraction

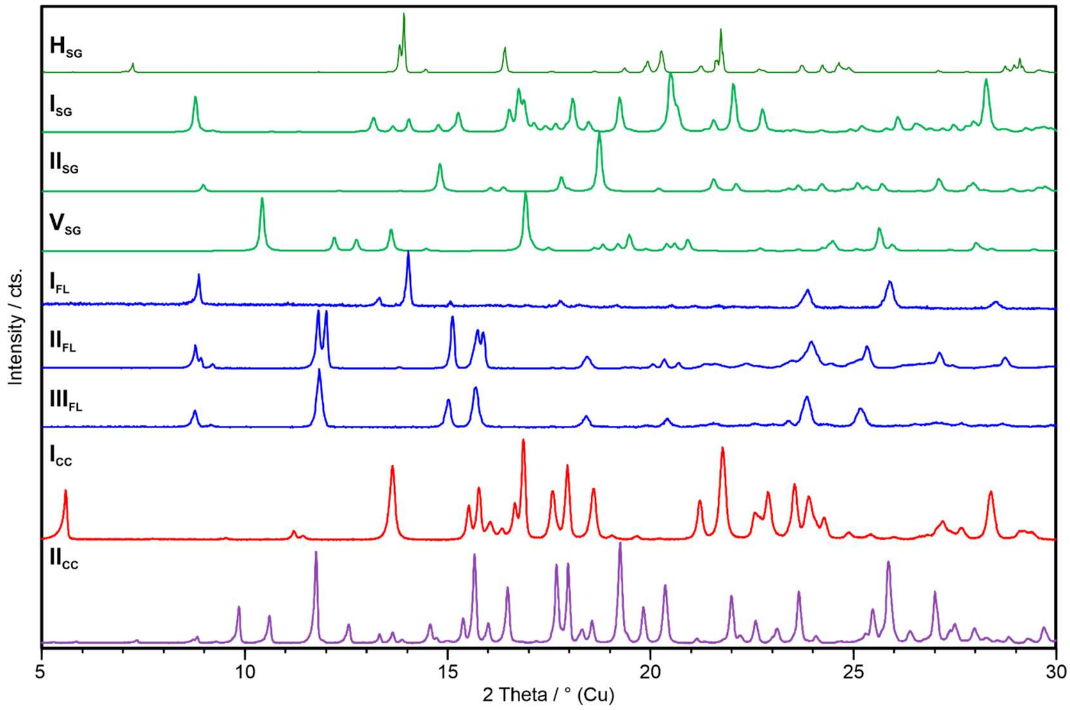

Figure S19 PXRD patterns of the starting materials and the cocrystals with the sulfaguanidine polymorphs shown in green, the flavone polymorphs shown in blue and the two cocrystal polymorphs in red and purple, respectively.

9.2. IR spectroscopy

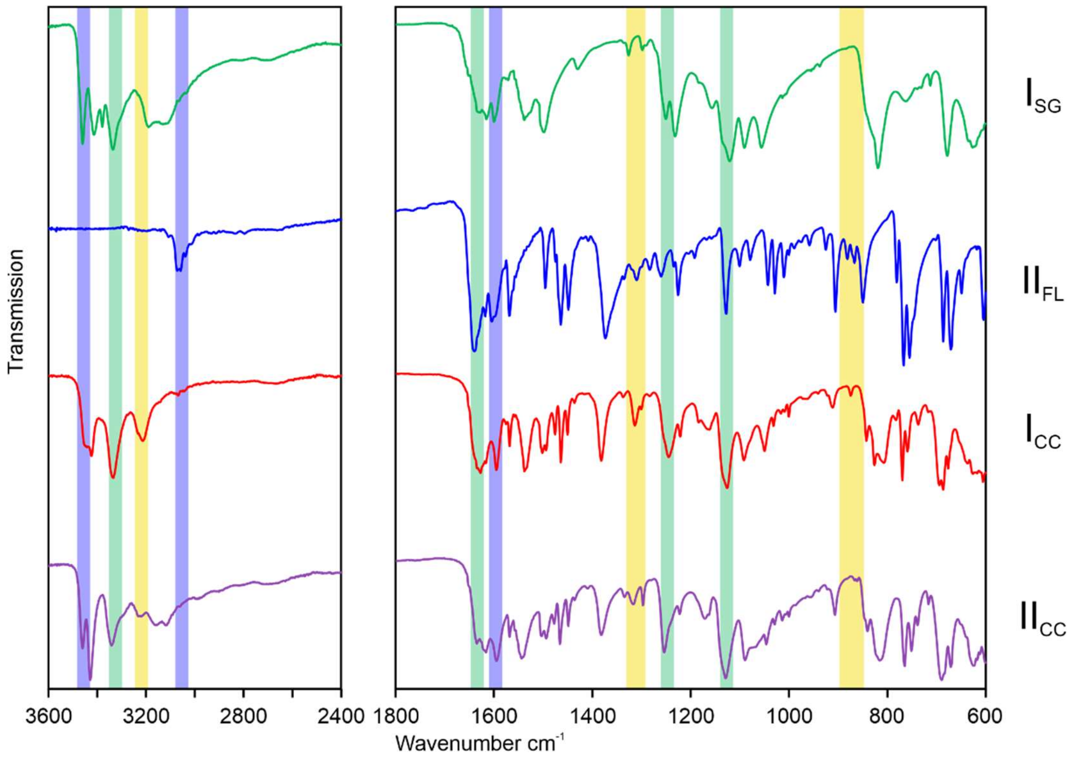

Figure S20. IR specrta of the sulfaguanidine/flavone cocrystals compared to the educts. Highlighted are the key bands of sulfagunainde (green) and flavone (blue) in the cocrystal structures. Other key regions are marked in yellow.

9.3. Gravimetric Moisture (de)sorption experiments

The gravimetric moisture (de)sorption behavior was analyzed using a SPS23 (ProUmid, Ulm, Germany) instrument with approximately 150 mg of substance. The humidity was stepwise increased from 0 to 95%, followed by a stepwise decrease to 0% relative humidity. The humidity changes were set to 5% per step and the equilibrium conditions to a mass constancy of ±0.001% over 48min and a maximum time limit of 48 hours per step.

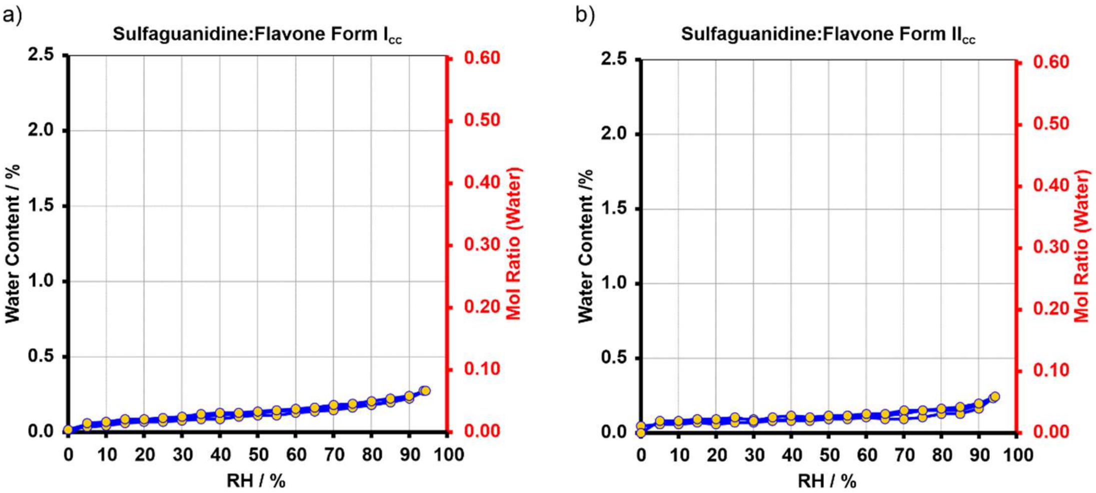

Figure S21. Moisture (de)sorption of sulfaguanidine/flavone cocrystals a) form I_{CC} and b) form II_{CC}.

10. Dapsone/flavone cocrystals

10.1. Powder X-ray diffraction

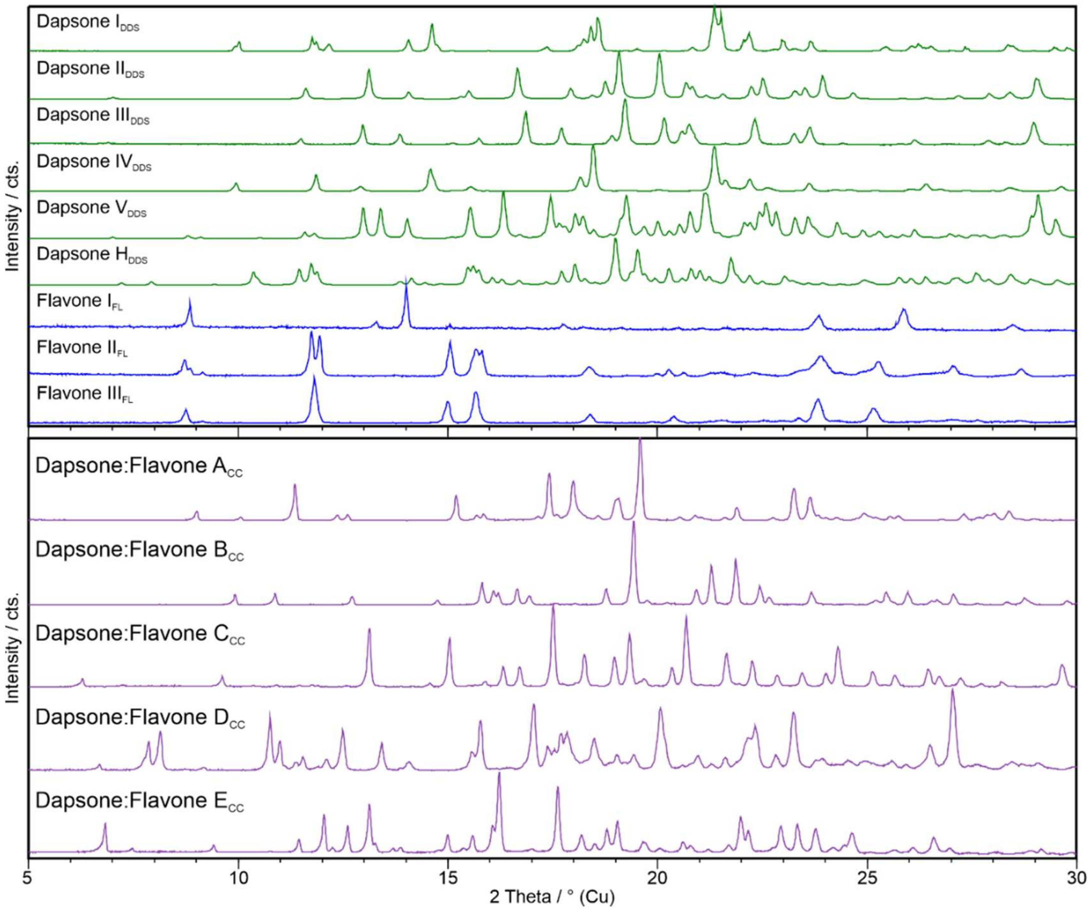

Figure S22. PXRD patterns of the dapsone/flavone cocrystals **A$_{CC}$**, **B$_{CC}$**, **C$_{CC}$**, **D$_{CC}$**, and **E$_{CC}$** and their educts (dapsone and flavone).

10.2. IR spectroscopy

Table S24 lists key bands positions to discriminate the cocrystal forms.

Table S23. Key band positions of the DDS/FL cocrystals.

	υ(N-H)	υ(S=O)	υ(C=O)
A$_{CC}$	3335 and 3231	1287 and 1142	1637
B$_{CC}$	3376 and 3202	1283 and 1143	1642
C$_{CC}$	3372 and 3220	1282 and 1145	1635
D$_{CC}$	3336 and 3190	1284 and 1145	1638
E$_{CC}$	3350 and 3194	1282 and 1132	1647

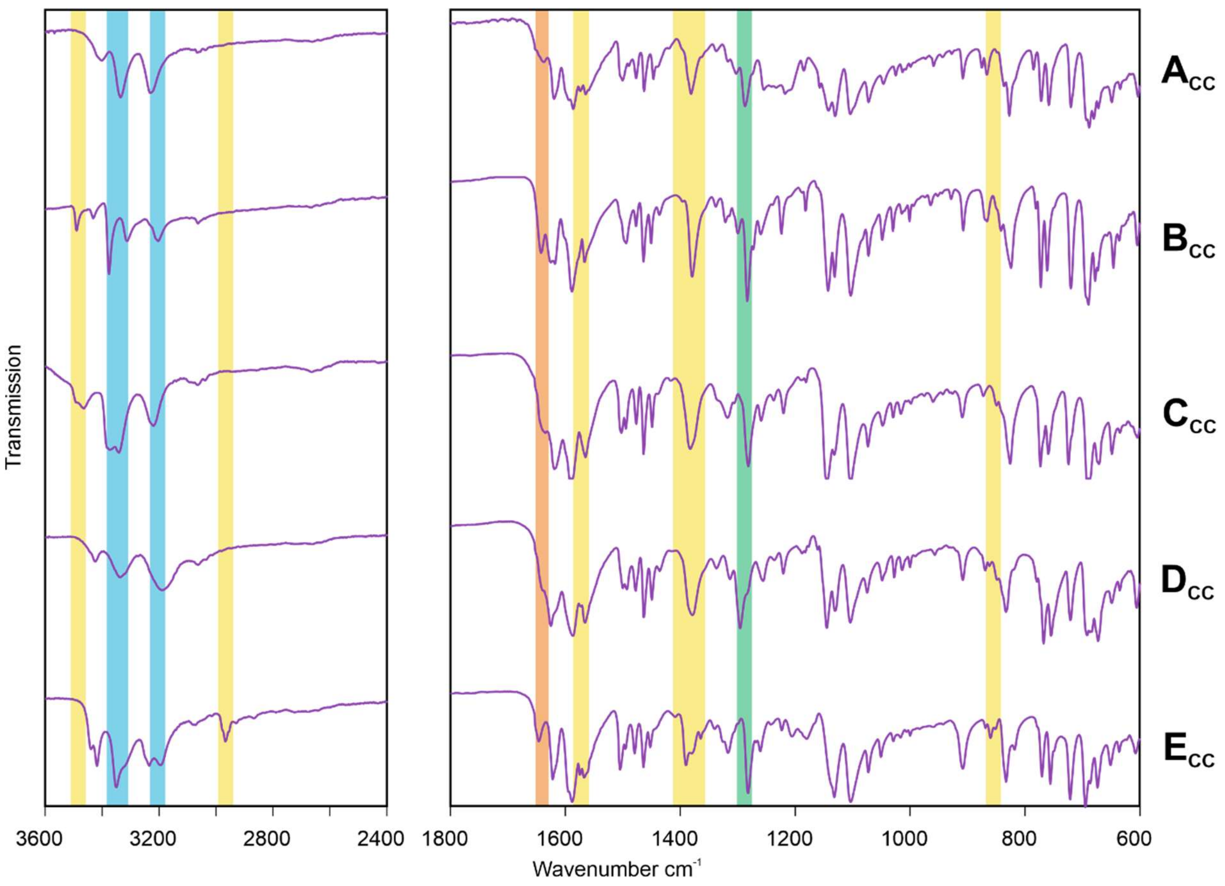

Figure S23. IR spectra of dapsone/flavone cocrystals A_{CC}, B_{CC}, C_{CC}, D_{CC}, and E_{CC}. Key regions are highlighted in green (S=O), orange (C=O), and blue (N-H). Additional band positions used for discriminating between the cocrystals are highlighted in yellow.

10.3. Gravimetric Moisture (de)sorption experiments

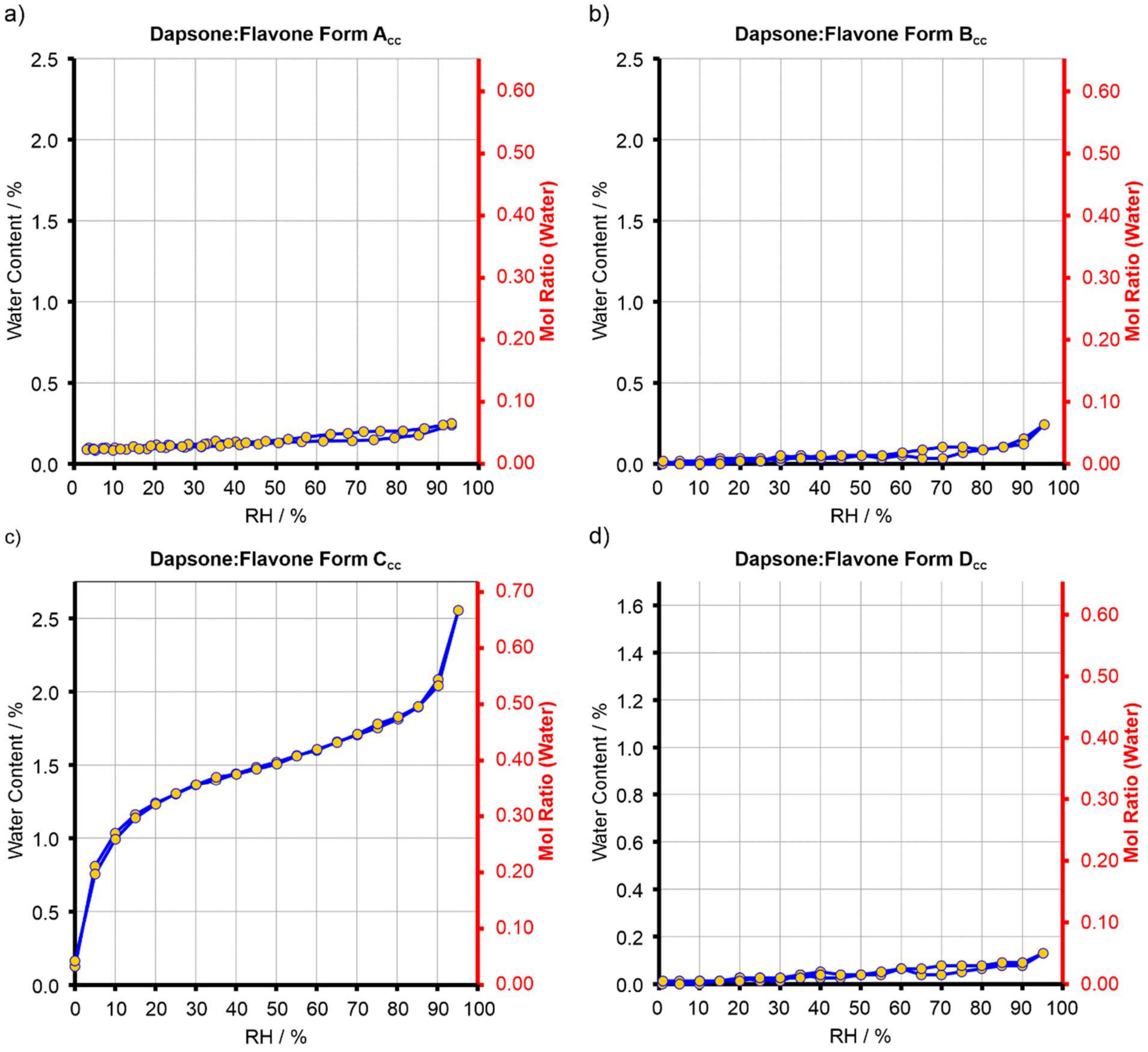

Figure S24. Moisture (de)sorption isotherms of the dapsone/flavone cocrystals A_{CC}, B_{CC}, C_{CC}, and D_{CC}.

11. Pairwise intermolecular energy calculations

11.1. Flavone form I

Table S24. Pairwise intermolecular interactions seen in I_{FL}.

N	Symmetry operation	R Å	E_{ele}	E_{pol}	E_{dis}	E_{rep}	E_{tot}[a]
			kJ mol^{-1}				
2	-	3.87	-11.8	-2.3	-74.5	58.5	-42.9
2	-	4.69	-9.6	-1.5	-59.6	35.5	-41.3
2	-	6.97	-29.3	-10.1	-17.7	29.4	-35.7
2	-x, y+1/2, -z+1/2	6.75	-13.5	-5.2	-24.7	27.2	-22.9
2	-	6.48	-7.4	-1.2	-27.2	20.3	-19.9
2	-x, y+1/2, -z+1/2	7.14	-12.9	-4.2	-22.4	28.5	-18.7
2	-	9.62	-8.7	-2	-14.5	13.6	-15
2	-x+1/2, -y, z+1/2	10.03	-6	-0.6	-12.7	16.2	-7.9
2	-	10.66	-2.5	-0.6	-10.9	10.5	-6.1
2	x+1/2, -y+1/2, -z	10.12	-2.1	-0.3	-5.2	1.2	-6.1
2	-x+1/2, -y, z+1/2	10	-0.6	-0.7	-9.5	5.8	-5.8
2	-	9.81	-0.1	-1.1	-13.1	12.3	-4.7
2	-	10.92	-0.4	-0.6	-9.9	9.7	-3.5
2	x+1/2, -y+1/2, -z	11.32	0.2	-0.1	-2.2	0	-1.8

[a] electrostatic (E_{ele}), polarization (E_{pol}), dispersion (E_{dis}), and exchange-repulsion (E_{res}). $E_{tot} = k_{ele} \cdot E_{ele} + k_{pol} \cdot E_{pol} + k_{dis} \cdot E_{dis} + k_{res} \cdot E_{res}$, with k being scale factors.

11.2. Flavone form II

Table S25. Pairwise intermolecular interactions seen in II_{FL}.

N	Symmetry operation	R Å	E_{ele}	E_{pol}	E_{dis}	E_{rep}	E_{tot}[a]
			kJ mol^{-1}				
1	-x, -y, -z	6.97	-46.5	-14.4	-19.3	55.1	-42.6
2	x, y, z	4.83	-7.3	-1.9	-64.9	44.8	-37.9
2	x, y, z	4.83	-5.8	-1.6	-62.4	39	-37.6
1	-x, -y, -z	6.97	-33	-11	-18.4	34.8	-37.5
1	-x, -y, -z	7.12	-6.4	-4.5	-19	12.1	-19.1
2	-	7.15	-13	-3.6	-17.2	20	-19
1	-x, -y, -z	7.17	-3.5	-3	-14.7	4.4	-16
2	-	8.62	-10.1	-3.1	-14.2	17.3	-14.7
2	-	8.61	-4.3	-0.7	-16.3	13.1	-11.2
2	-	7.31	-2.4	-0.9	-14.4	10.6	-9.1
2	-x+1/2, y+1/2, -z+1/2	9.02	-3.5	-0.7	-14.1	14.5	-7.5
2	-	9.75	-2	-0.7	-12.3	10	-7.1
2	-x+1/2, y+1/2, -z+1/2	9.13	-3.7	-0.7	-13.3	14.6	-7
2	-	9.88	-4.6	-0.8	-13.9	17.2	-6.9
2	-	12.02	-2.7	-0.7	-13.7	13.8	-6.8
2	-	9.9	-1.2	-0.6	-12.7	11.5	-5.6
2	-	11.99	0.8	-0.1	-1.7	0.2	-0.6

[a] electrostatic (E_{ele}), polarization (E_{pol}), dispersion (E_{dis}), and exchange-repulsion (E_{res}). $E_{tot} = k_{ele} \cdot E_{ele} + k_{pol} \cdot E_{pol} + k_{dis} \cdot E_{dis} + k_{res} \cdot E_{res}$, with k being scale factors.

11.3. Sulfaguanidine form I

Table S26. Pairwise intermolecular interactions seen in I_{SG}.

N	Symmetry operation	R Å	E_{ele}	E_{pol}	E_{dis}	E_{rep}	E_{tot}[a]
			kJ mol^{-1}				
2	-	5.51	-128.3	-31.1	-36.2	117.9	-117.4
1	-x, -y, -z	6.78	-126.5	-29.5	-30.6	113.3	-112.3
1	-x, -y, -z	7.85	-59.9	-11.2	-29.6	23.2	-83
2	-	5.01	-53.6	-11.8	-34.6	32.7	-75.3
1	-x, -y, -z	5.78	-67.1	-18.7	-27.6	57.7	-73.2
2	x, y, z	6.97	-75.5	-18.7	-12.2	57.3	-68.9
1	-x, -y, -z	6.94	-38.5	-11.2	-25.1	18.2	-59.6
2	-	6.45	-41	-10.3	-28.8	34.7	-54.6
2	-	8.62	-46.5	-10.9	-9.5	29.5	-47.2
2	x, y, z	6.97	-38.6	-9.4	-8.1	16.7	-44.5
2	-	6.24	-25.6	-10.8	-37.7	38.9	-43.9
2	x, y, z	6.97	-30.4	-8	-9	7.6	-41.1
2	-	9.3	-31.9	-8.4	-13.8	35.3	-30.3
2	-	6.36	-16.5	-10.9	-29.5	39.6	-26.8
2	-	9.5	-14.7	-5.7	-9.3	12.5	-20.2
2	-	8.91	-31.4	-12.6	-17.4	64.2	-18
2	-	7.01	1.8	-2.9	-28.4	14.7	-16
2	-	8.31	-10.5	-1	-3.9	0.3	-15
2	-	10.33	-4.2	-2.4	-6	4	-9
2	-	9.28	-1.8	-0.5	-4.2	0.9	-5.4
1	-x, -y, -z	8.88	1.6	-0.9	-1.9	0	-0.6
1	-x, -y, -z	9.47	5.2	-3.1	-5.4	1.5	-0.6
1	-x, -y, -z	13.34	2	-0.3	-1	0	1
2	-	6.54	14.9	-5	-16.2	8.7	3.3
2	-	9.38	15	-2.5	-4.1	1	11
2	-	9.62	14	-1.1	-1.8	0	12.5
1	-x, -y, -z	7.95	27.3	-5.8	-4.6	0.7	20.9
2	-	7.98	33.4	-4.8	-8.8	2.8	25.9

[a] electrostatic (E_{ele}), polarization (E_{pol}), dispersion (E_{dis}), and exchange-repulsion (E_{res}). $E_{tot} = k_{ele} \cdot E_{ele} + k_{pol} \cdot E_{pol} + k_{dis} \cdot E_{dis} + k_{res} \cdot E_{res}$, with k being scale factors.

11.4. Sulfaguanidine form II

Table S27. Pairwise intermolecular interactions seen in II_{SG}.

N	Symmetry operation	R (Å)	E_{ele}	E_{pol}	E_{dis}	E_{rep}	E_{tot}[a]
			kJ mol^{-1}				
1	-x, -y, -z	7.38	-84.2	-18.6	-20.2	46.3	-91.8
1	-x, -y, -z	5.39	-92.5	-20.4	-65.1	132.8	-87.6
2	-	5.73	-52.3	-16.5	-41.2	74.2	-57.5
2	-	5.75	-57.5	-16.8	-35.6	84.3	-52.2
2	-	6.21	-47.7	-12.2	-17.2	43.8	-47.4
2	x, y, z	7.43	-35.5	-7.3	-5.8	10.6	-41.4
2	-x, y+1/2, -z+1/2	6.21	-16.4	-9	-34.5	27.4	-37.1
2	-	7.99	-31.5	-16.3	-17.6	44.2	-33.4
2	x, y, z	7.43	-12.2	-3.2	-5.5	1.4	-19.2
1	-x, -y, -z	7	-3.3	-4	-12.2	3	-15.2
2	-	6.04	2.4	-6.8	-21.5	15.2	-11.8
2	-	8	2	-7.9	-17	11.8	-11.2
2	-	8.6	-0.8	-1.7	-3.3	0.2	-4.9
2	-x, y+1/2, -z+1/2	7.72	3.7	-6.8	-9.1	7	-4.8
2	x, y, z	9.81	6.6	-3.1	-8.7	3	-0.9
2	x, y, z	9.81	14.8	-3.8	-13.4	10.7	7.8
1	-x, -y, -z	8.62	24.1	-3	-2.5	0.2	21.2

[a] electrostatic (E_{ele}), polarization (E_{pol}), dispersion (E_{dis}), and exchange-repulsion (E_{res}). $E_{tot} = k_{ele} \cdot E_{ele} + k_{pol} \cdot E_{pol} + k_{dis} \cdot E_{dis} + k_{res} \cdot E_{res}$, with k being scale factors.

11.5. Sulfanilamide form α

Table S28. Pairwise intermolecular interactions seen in α_{SA}.

N	Symmetry operation	R (Å)	E_{ele}	E_{pol}	E_{dis}	E_{rep}	E_{tot}[a]
			kJ mol^{-1}				
1	-x, -y, -z	7.13	-46.8	-9.6	-11.5	26.8	-50.1
2	-x+1/2, -y, z+1/2	5.4	-41.8	-9.6	-29.6	44.2	-49.7
2	x, -y+1/2, z+1/2	4.76	-29.5	-9.7	-31.2	55.7	-31.2
2	x+1/2, y, -z+1/2	9.19	-30.5	-6.1	-12.7	27.3	-30.9
2	x, y, z	5.5	-16.8	-4.8	-19.9	16	-28.8
2	x+1/2, -y+1/2, -z	10.2	3.9	-0.6	-2.3	0.2	1.8
2	x+1/2, y, -z+1/2	10.41	4.1	-0.9	-2.3	0.2	1.8
1	-x, -y, -z	7.55	29.9	-4.5	-6.1	4	25.5

[a] electrostatic (E_{ele}), polarization (E_{pol}), dispersion (E_{dis}), and exchange-repulsion (E_{res}). $E_{tot} = k_{ele} \cdot E_{ele} + k_{pol} \cdot E_{pol} + k_{dis} \cdot E_{dis} + k_{res} \cdot E_{res}$, with k being scale factors.

11.6. Sulfanilamide form β

Table S29. Pairwise intermolecular interactions seen in β_{SA}.

N	Symmetry Operation	R Å	E_{ele}	E_{pol}	E_{dis}	E_{rep}	E_{tot}[a]
			kJ mol^{-1}				
2	-	4.93	-42.3	-9.6	-21.9	38.4	-47.2
2	-	4.95	-40.9	-9.3	-21.7	35.9	-46.9
2	-	6.31	-32	-9.5	-17.6	33.4	-35.6
2	-	6.25	-34.6	-10.1	-19.2	40.9	-35.5
2	x+1/2, -y+1/2, z	8.4	-36.3	-8.3	-9.4	28.2	-35.4
2	x+1/2, -y+1/2, z	8.4	-36.8	-8.4	-9.4	29.3	-35.3
2	-	6.22	-19.7	-3.7	-11.9	6.7	-29.8
2	-x+1/2, y+1/2, -z	6.63	-18.1	-5.9	-23.2	26.1	-27.6
2	-x+1/2, y+1/2, -z	6.63	-18.2	-5.9	-23.3	26.4	-27.5
1	-x, -y, -z	8.28	-8.8	-1.9	-3.4	0.3	-13.4
1	-x, -y, -z	8.32	-7.8	-1.8	-3.1	0.2	-12.2
1	-x, -y, -z	7.63	-8.2	-0.7	-2.2	0	-11.1
1	-x, -y, -z	7.7	-7.8	-0.6	-2.1	0	-10.5
2	-	6.13	8.7	-2.9	-36.1	24	-9.5
1	-x, -y, -z	13.01	5	-0.4	-0.7	0	4.3
1	-x, -y, -z	13	5.1	-0.5	-0.8	0	4.4
2	-	8.99	10.1	-1.7	-6.4	2.8	5.6
2	-	9	10.3	-1.6	-6.1	2.4	5.8
1	-x, -y, -z	7.75	18.7	-2.2	-2.3	0.3	16.2
1	-x, -y, -z	7.72	18.9	-2.3	-2.4	0.4	16.4

[a] electrostatic (E_{ele}), polarization (E_{pol}), dispersion (E_{dis}), and exchange-repulsion (E_{res}). $E_{tot} = k_{ele} \cdot E_{ele} + k_{pol} \cdot E_{pol} + k_{dis} \cdot E_{dis} + k_{res} \cdot E_{res}$, with k being scale factors.

11.7. Sulfanilamide form γ

Table S30. γ_{SA}: Pairwise intermolecular interactions seen in γ_{SA}.

N	Symmetry operation	R Å	E_{ele}	E_{pol}	E_{dis}	E_{rep}	E_{tot}[a]
			kJ mol^{-1}				
1	-x, -y, -z	5.94	-83.3	-18.2	-25.6	75.4	-77.3
1	-x, -y, -z	5.34	-25.3	-5.3	-24.4	19.5	-39.8
2	x, y, z	9.32	-26.3	-5.4	-5.3	13.5	-28.1
2	-x, y+1/2, -z+1/2	6.95	-20.9	-4.4	-11.6	15.1	-26
2	x, -y+1/2, z+1/2	4.97	-22.3	0	-23	39.2	-19.4
2	x, -y+1/2, z+1/2	8.33	-2.9	-4.2	-9	6.7	-9.8
1	-x, -y, -z	10.85	-5	-2.4	-9.3	13.1	-7
1	-x, -y, -z	5.93	18.9	-4	-33.7	17.8	-1.3
2	x, y, z	7.74	5.6	-1.9	-9.8	4.6	-1.2

[a] electrostatic (E_{ele}), polarization (E_{pol}), dispersion (E_{dis}), and exchange-repulsion (E_{res}). $E_{tot} = k_{ele} \cdot E_{ele} + k_{pol} \cdot E_{pol} + k_{dis} \cdot E_{dis} + k_{res} \cdot E_{res}$, with k being scale factors.

11.8. Sulfanilamide form δ

Table S31. Pairwise intermolecular interactions seen in δ_{SA}.

N	Symmetry operation	R Å	E_{ele}	E_{pol}	E_{dis}	E_{rep}	E_{tot} [a]
			kJ mol^{-1}				
1	-x, -y, -z	6.21	-64.2	-13.5	-20.5	47.6	-66.4
2	x+1/2, -y+1/2, -z	6.03	-30.5	-6.3	-23.8	34.7	-36.1
2	x+1/2, y, -z+1/2	5.17	-16.5	-5.1	-23.1	16.8	-30.9
2	-x+1/2, -y, z+1/2	7.13	-29	-7.7	-8.5	25.5	-28
2	-x+1/2, y+1/2, z	8.99	-25.3	-5.7	-12.7	23.6	-27.5
2	x, -y+1/2, z+1/2	6.18	-9.8	-2.9	-18.9	12.1	-21.5
2	-x, y+1/2, -z+1/2	9.86	6.3	-1.1	-4.8	2.2	3
1	-x, -y, -z	7.71	24.7	-3.5	-4.2	2.2	21.3

[a] electrostatic (E_{ele}), polarization (E_{pol}), dispersion (E_{dis}), and exchange-repulsion (E_{res}). $E_{tot} = k_{ele} \cdot E_{ele} + k_{pol} \cdot E_{pol} + k_{dis} \cdot E_{dis} + k_{res} \cdot E_{res}$, with k being scale factors.

11.9. Dapsone form I

Table S32. Pairwise intermolecular interactions seen in I_{DDS}.

N	Symmetry Operation	R Å	E_{ele}	E_{pol}	E_{dis}	E_{rep}	E_{tot} [a]
			kJ mol^{-1}				
1	-x, -y, -z	6.52	-21.7	-7.8	-56.4	40.7	-52.7
1	-x, -y, -z	6.53	-20	-7.5	-56.6	39.8	-51.4
2	-	6.46	-27.5	-6.8	-37.8	36.5	-44.5
1	-x, -y, -z	9.29	-19.9	-2.5	-18.9	13.6	-30.9
1	-x, -y, -z	9.74	-19.7	-2.4	-20.4	16.3	-30.3
2	x, -y+1/2, z+1/2	8.61	-26.3	-7.6	-10.2	19.6	-30.2
2	x, -y+1/2, z+1/2	8.78	-27	-7.7	-9.1	19.5	-30
2	x, y, z	8.28	-27	-8.9	-13.7	29.2	-29
2	x, y, z	8.28	-24	-8.1	-11.4	23.7	-26.6
2	-x, y+1/2, -z+1/2	6.22	-5.6	-6	-26.8	19	-21.9
2	-	7.6	-11.7	-5.7	-20.3	20.9	-21.4
2	-x, y+1/2, -z+1/2	6.32	-4.5	-5.2	-24.4	15.1	-20.5
2	-	9.23	-9.1	-0.9	-4.6	0.1	-14.3
2	-	8.65	-5.3	-1.7	-10.2	6.3	-11.9
2	-	10.29	-5.7	-1.3	-7.2	7.4	-8.7
2	-	7.87	8.6	-1.6	-20.1	9.6	-3.7
2	-	11.44	3	-1.3	-4.9	1.2	-1.3

[a] electrostatic (E_{ele}), polarization (E_{pol}), dispersion (E_{dis}), and exchange-repulsion (E_{res}). $E_{tot} = k_{ele} \cdot E_{ele} + k_{pol} \cdot E_{pol} + k_{dis} \cdot E_{dis} + k_{res} \cdot E_{res}$, with k being scale factors.

11.10. Dapsone form II

Table S33. Pairwise intermolecular interactions seen in II_{DDS}.

N	Symmetry Operation	R Å	E_{ele}	E_{pol}	E_{dis}	E_{rep}	E_{tot}[a]
			kJ mol^{-1}				
2	-x, y+1/2, -z+1/2	6.8	-30.1	-6.9	-37	38.7	-45.3
2	-x, y+1/2, -z+1/2	7.03	-15	-7	-35.5	25.6	-36.1
2	x, y, z	7.9	-30.6	-9.5	-12.1	34.9	-28.4
2	x+1/2, -y+1/2, -z	8.25	-6.8	-3.3	-20.6	11.8	-20.2
2	x, y, z	5.77	1.3	-5.9	-29.5	19.7	-16.5
2	x+1/2, -y+1/2, -z	12.19	-6.2	-2.6	-9.1	8.5	-11.2

[a] electrostatic (E_{ele}), polarization (E_{pol}), dispersion (E_{dis}), and exchange-repulsion (E_{res}). $E_{tot} = k_{ele} \cdot E_{ele} + k_{pol} \cdot E_{pol} + k_{dis} \cdot E_{dis} + k_{res} \cdot E_{res}$, with k being scale factors.

11.11. Dapsone form III

Table S34. Pairwise intermolecular interactions seen in III_{DDS}.

N	Symmetry Operation	R Å	E_{ele}	E_{pol}	E_{dis}	E_{rep}	E_{tot}[a]
			kJ mol^{-1}				
2	-x, y+1/2, -z+1/2	7.15	-27.7	-6.3	-33.3	32.1	-43.1
2	-x, y+1/2, -z+1/2	7.09	-16.2	-7.2	-36.6	28.3	-36.8
2	x, y, z	7.98	-31.6	-9.6	-12.5	36	-29.2
2	x+1/2, -y+1/2, -z	11.57	-20.2	-4.5	-13.3	25.3	-20.6
2	x, y, z	5.5	3.7	-6.9	-33.4	23.8	-15.6
2	x+1/2, -y+1/2, -z	8.28	-2.7	-2.7	-13.7	4.4	-14.1
2	x, y, z	9.69	-9.9	-1.5	-2.1	0	-13.4

[a] electrostatic (E_{ele}), polarization (E_{pol}), dispersion (E_{dis}), and exchange-repulsion (E_{res}). $E_{tot} = k_{ele} \cdot E_{ele} + k_{pol} \cdot E_{pol} + k_{dis} \cdot E_{dis} + k_{res} \cdot E_{res}$, with k being scale factors.

11.12. Dapsone form V

Table S35. Pairwise intermolecular interactions seen in V_{DDS}.

N	Symmetry operation	R Å	E_{ele}	E_{pol}	E_{dis}	E_{rep}	E_{tot}[a]
			kJ mol^{-1}				
2	-	7.26	-26.3	-8.1	-40.1	30.1	-50.2
2	-	6.96	-29	-7.5	-36.1	35.8	-45.5
2	-	7.03	-30	-6.9	-35.9	37.5	-45
2	-	7.05	-29.1	-6.4	-35.8	35.7	-44.6
2	-	6.64	-33.3	-9	-32.8	41.8	-44.6
1	-x, -y, -z	7.65	-22.3	-8.5	-32.5	23.3	-43.8
2	-	7.14	-22.7	-7.3	-35.1	28.7	-42.3
2	-	7.25	-16.7	-6.3	-39.1	31.3	-37
2	-	7.76	-35.7	-9.7	-14.2	35.8	-35.2
2	-	8.34	-31.3	-8.1	-11.8	23.4	-34.9
2	-	8.25	-33.1	-9.5	-11.5	35.7	-30
2	-	7.85	-10	-4.5	-30.2	17.5	-29.4
2	-	8.02	-24.2	-6.9	-10.9	20	-27.9
2	x, y, z	5.7	-9.6	-9.1	-35.6	33.4	-27.2
2	-x+1/2, y+1/2, -z+1/2	11.65	-16.6	-3.3	-13.2	15.2	-22.1
2	-	11.22	-17.6	-4	-13.1	19.8	-20.7
2	-	8.29	-10.6	-5.2	-12.5	12.3	-18.4
2	x, y, z	5.7	0.7	-6.5	-29.9	20.6	-17.4
2	-	11.76	-12.7	-3.5	-12.2	15.5	-17.1
2	-x+1/2, y+1/2, -z+1/2	8.11	-3.7	-3.1	-15.2	5.3	-16.2
2	-	9.18	-9.3	-1.7	-10.3	6.8	-15.8
2	x, y, z	5.7	3.8	-6.1	-29.7	18.8	-14.7
2	-	8.83	-7.7	-3	-6.1	1.8	-14.5
2	x, y, z	5.7	5	-5.7	-30.5	20	-13.1
1	-x, -y, -z	11.01	-6.2	-0.7	-3.5	0.3	-10
1	-x, -y, -z	11.56	-0.8	-2.8	-11.4	5.8	-9.3
2	-	7.9	4.2	-2.9	-18.6	10.8	-7.3
1	-x, -y, -z	12.8	-3.4	-1.7	-8	8.2	-6.7
2	-	13.58	4.9	-0.6	-1.4	0.1	3.5

[a] electrostatic (E_{ele}), polarization (E_{pol}), dispersion (E_{dis}), and exchange-repulsion (E_{res}). $E_{tot} = k_{ele} \cdot E_{ele} + k_{pol} \cdot E_{pol} + k_{dis} \cdot E_{dis} + k_{res} \cdot E_{res}$, with k being scale factors.

11.13. Dapsone/flavone A_{CC}

Table S36. Pairwise intermolecular interactions seen in A_{CC} [dapsone/flavone (1:1) form A].

N	Symmetry operation	R Å	E_{ele}	E_{pol}	E_{dis}	E_{rep}	E_{tot}[a]
			kJ mol^{-1}				
2	-	7.3	-18.3	-3.4	-33.8	26.9	-34.7
2	-	9.83	-43.3	-11.1	-13.5	51.6	-33.9
2	-	6.36	-14	-6.4	-35.8	32.6	-30.6
2	-	9.76	-39.4	-9.6	-10.9	48.5	-28.3
2	-x+1/2, y+1/2, -z+1/2	9.41	-31.1	-9.2	-13	38	-27.5
1	-x, -y, -z	7.3	-6.6	-1.5	-36.5	24.4	-24.8
2	-	5.31	-6.5	-3.2	-36.9	28.1	-24
2	-	4.72	-0.1	-2.5	-36.4	16.3	-23.6
2	-	9.08	-6.9	-3.2	-19.7	18.6	-15.3
2	x, y, z	11.2	-20.3	-5.5	-13	37.4	-13.8
2	-x+1/2, y+1/2, -z+1/2	8.61	-7.1	-2.1	-4.1	0.8	-12.2
2	-x+1/2, y+1/2, -z+1/2	7.93	-2.1	-0.3	-9.2	2.3	-9.1
1	-x, -y, -z	8.3	-1.5	-2.8	-4.2	0.3	-7.1
1	-x, -y, -z	6.82	1.4	-3.3	-21.9	22.1	-6.4
2	x, y, z	11.2	-1.5	-0.7	-4.9	1.2	-5.6
2	-	10.95	0.1	-1	-9	7.1	-4.2
1	-x, -y, -z	14.08	1.7	-0.4	-1.7	0.1	0.1

[a] electrostatic (E_{ele}), polarization (E_{pol}), dispersion (E_{dis}), and exchange-repulsion (E_{res}). $E_{tot} = k_{ele} \cdot E_{ele} + k_{pol} \cdot E_{pol} + k_{dis} \cdot E_{dis} + k_{res} \cdot E_{res}$, with k being scale factors.

11.14. Dapsone/flavone B_{CC}

Table S37. Pairwise intermolecular interactions seen in B_{CC} [dapsone/flavone (1:1) form B].

N	Symmetry operation	R Å	E_{ele}	E_{pol}	E_{dis}	E_{rep}	E_{tot}[a]
			kJ mol^{-1}				
1	-	7.1	-25.5	-3.6	-45.5	50.9	-37.8
1	x+3/4, -y+1/4, z+3/4	8.28	-29.7	-9.3	-18.6	35.2	-32.7
1	x+3/4, -y+1/4, z+3/4	8.28	-29.7	-9.3	-18.6	35.2	-32.7
1	-	10.02	-36.8	-10.7	-10.5	42	-30
1	x+3/4, -y+1/4, z+3/4	9.14	-20	-4.3	-8.8	7.6	-27.2
1	x+3/4, -y+1/4, z+3/4	9.14	-20	-4.3	-8.8	7.6	-27.2
1	-	9.42	-17	-5.8	-26.8	30.5	-26.7
1	-	6.23	-8	-1.2	-30.3	17	-25.2
1	-	7.72	-10.5	-3.8	-17.4	13.8	-20.6
1	-	8.59	-8.6	-2.5	-29.5	27.5	-19.7
1	x, y, z	5.49	-7.5	-8.7	-41.4	51.6	-18.5
1	x, y, z	5.49	-7.5	-8.7	-41.4	51.6	-18.5
1	-	10.02	-5.5	-1.3	-12.7	10.1	-11.5
1	-	6.4	1	-3	-12	2.1	-10.4
1	-x, -y, z	13.63	4.7	-1.5	-5.6	2.8	0.7

[a] electrostatic (E_{ele}), polarization (E_{pol}), dispersion (E_{dis}), and exchange-repulsion (E_{res}). $E_{tot} = k_{ele} \cdot E_{ele} + k_{pol} \cdot E_{pol} + k_{dis} \cdot E_{dis} + k_{res} \cdot E_{res}$, with k being scale factors.

11.15. Dapsone/flavone D_{CC}

Table S38. Pairwise intermolecular interactions seen in D_{CC} [dapsone/flavone (1:2) form D].

N	Symmetry operation	R Å	E_{ele}	E_{pol}	E_{dis}	E_{rep}	E_{tot}[a]
			kJ mol^{-1}				
1	-	9.43	-39.9	-10.3	-12.5	46.5	-32
1	-	8.78	-28.4	-7.4	-9.9	23.1	-29.8
1	-	7.93	-17	-2.9	-25	23.4	-27.4
1	-	7.16	-12.7	-6.2	-23.8	23.8	-24.1
1	-	6.44	-15.8	-6.4	-24.8	30.7	-24
1	-	10.02	-29.8	-6.8	-9.9	34.8	-23.7
1	-	6.8	-6.6	-2.1	-28.7	15.9	-23.7
1	x, y, z	11.09	-33.8	-8.7	-14.3	56.4	-19.7
1	x, y, z	11.09	-33.8	-8.7	-14.3	56.4	-19.7
1	-	5.09	3.9	-2.9	-39	20.8	-19.1
1	-	6.51	-11.8	-7	-30.6	42.7	-17.9
1	-	8.74	-10.3	-4.1	-21.6	25.9	-16.8
1	-	7.36	-7.2	-1.9	-29.5	28.9	-16.8
1	-	10.17	-0.4	-1.1	-11.1	7.7	-6.2
1	-	12.97	2.9	-0.5	-1.7	0.1	1.3
1	-	9.36	5.3	-0.8	-2.8	0.1	2.6

[a] electrostatic (E_{ele}), polarization (E_{pol}), dispersion (E_{dis}), and exchange-repulsion (E_{res}). $E_{tot} = k_{ele} \cdot E_{ele} + k_{pol} \cdot E_{pol} + k_{dis} \cdot E_{dis} + k_{res} \cdot E_{res}$, with k being scale factors.

11.16. Dapsone/flavone E_{CC}

Table S39. Pairwise intermolecular interactions seen in E_{CC} [dapsone/flavone/*t*-butanol (1:1:1) form E].

N	Symmetry Operation	R Å	E_{ele}	E_{pol}	E_{dis}	E_{rep}	E_{tot}[a]
			kJ mol^{-1}				
1	-x, -y, -z	3.82	-10.7	-2.1	-72	44	-48.4
2	-	5.72	-56.7	-14.4	-16.1	75.2	-38.2
2	x, -y+1/2, z+1/2	8.53	-34.3	-9.7	-13.5	34.9	-33.6
2	-	10.49	-42.6	-11.7	-12.9	51.6	-33.2
2	-	7.27	-46.2	-12.6	-19.4	69.7	-32
2	x, y, z	8.19	-31.7	-11	-18.9	45.5	-30
2	-	8.72	-15.1	-3.2	-15.9	18.5	-20.7
2	-	4.43	-5	-1.1	-28	16.7	-20.2
2	-	6.91	-8.9	-2.2	-28.6	28.5	-18.4
2	-	9.81	-11.4	-3.8	-15.9	17.2	-18.1
2	-	8.7	-7.7	-2	-4.5	0.6	-13.2
2	-	6.82	3.1	-1	-19.4	8.4	-9.1
1	-x, -y, -z	5.32	-3.2	-1.8	-8.9	6.5	-8.5
2	-	8.76	-1.9	-2.3	-21.3	24.1	-7.3
2	-	5.99	0	-0.4	-11.7	7.6	-5.8
2	-	7.37	-0.3	-1.5	-11.6	9.4	-5.7
2	-	7.76	-1.4	-0.9	-2.1	0	-4
2	-	10.98	0.4	-0.9	-7.2	4.2	-3.9
2	-	8.17	-2.3	-0.3	-8.5	10	-3.9
2	x, -y+1/2, z+1/2	11.55	-2.2	-0.4	-1.4	0	-3.8
2	-	8.91	0	-0.2	-6.4	3.6	-3.5
1	-x, -y, -z	6.61	-0.3	0	-3.4	0.5	-2.9
1	-x, -y, -z	8.2	2	-0.4	-3.5	0.1	-1.2
2	x, -y+1/2, z+1/2	11.3	3.7	-0.6	-2.9	0.2	1.1

[a] electrostatic (E_{ele}), polarization (E_{pol}), dispersion (E_{dis}), and exchange-repulsion (E_{res}). $E_{tot} = k_{ele} \cdot E_{ele} + k_{pol} \cdot E_{pol} + k_{dis} \cdot E_{dis} + k_{res} \cdot E_{res}$, with k being scale factors.

11.17. Sulfaguanidine/flavone II$_{CC}$

Table S40. Pairwise intermolecular interactions seen in II$_{CC}$ [sulfaguanidine/flavone (1:1) form II].

N	Symmetry operation	R Å	E_{ele}	E_{pol}	E_{dis}	E_{rep}	E_{tot}[a]
			kJ mol^{-1}				
2	-	6.9	-111.1	-25.9	-25.5	85.7	-105.9
2	x, y, z	7.03	-67.6	-17	-13.5	54	-62.5
2	x, y, z	7.03	-69.9	-17.9	-13.4	63.5	-59.6
2	-	7.81	-38.8	-9.3	-12.5	34.2	-37.6
2	-	8.43	-42.4	-10.8	-11.3	47.9	-33
2	-	8.56	-22.2	-4.8	-14.8	12.5	-32.1
2	-	4.15	1.7	-2.8	-56.7	31.5	-30.2
2	-	6.37	-10.9	-3	-34.7	23.2	-29.6
2	-	5.23	-3.2	-2.3	-36.6	18.8	-25.3
2	-	7.17	-6.9	-2.5	-33.3	23.2	-23.8
2	x+1/2, -y+1/2, -z	5.88	-3.9	-0.8	-30.1	12.3	-23.3
2	-	9.45	-13.4	-6.4	-9.5	10.4	-20.7
2	-	5.49	-5.6	-8.5	-29.6	28.6	-20.3
2	-	9.79	-11.8	-2	-6.4	1.7	-18.5
2	-	9.94	-10.9	-4.4	-5.6	4.3	-16.9
2	-	5.51	-2.2	-9	-31.9	33.7	-15.9
2	-	9.1	-9.6	-2.1	-10.8	9.2	-15.4
2	-	10.26	-6.3	-3.7	-10	6	-14.3
2	-	7.89	-4	-1.8	-12.2	3.3	-14.1
2	-	9.34	-4.5	-3.4	-9.4	6.8	-11.3
2	-	7.78	0.5	-0.9	-19.2	10.7	-10.2
2	-	9.25	-1.7	-2.8	-5.8	3.2	-7
2	-	11.98	-2.8	-1.4	-6.7	5.2	-6.7
2	-	11.22	-2.4	-1.4	-2.6	0.3	-5.6
2	-	10.35	4.4	-1.6	-6	1.8	-0.7
2	-	9.9	6.4	-2.4	-8.8	3.8	-0.4
2	x+1/2, -y+1/2, -z	12.64	2.7	-0.4	-1.4	0	1.4
2	-	11.1	13.6	-2.2	-3.2	0.6	10.3

[a] electrostatic (E_{ele}), polarization (E_{pol}), dispersion (E_{dis}), and exchange-repulsion (E_{res}). $E_{tot} = k_{ele} \cdot E_{ele} + k_{pol} \cdot E_{pol} + k_{dis} \cdot E_{dis} + k_{res} \cdot E_{res}$, with k being scale factors.

11.18. Sulfanilamide/flavone

Table S41. Pairwise intermolecular interactions seen in $SAFL_{CC}$ (sulfanilamide/flavone (1:1) form I).

N	Symmetry operation	R Å	E_{ele}	E_{pol}	E_{dis}	E_{rep}	E_{tot}[a]
			kJ mol^{-1}				
1	-x, -y, -z	6.06	-79.3	-18.2	-25.3	67.3	-77.9
2	-	6.81	-43.7	-11.6	-16.6	44	-42
2	-x+1/2, y+1/2, z	5.69	-9.5	-1.2	-53.2	34.6	-35.9
2	-	7.14	-41.1	-13.9	-15.7	65.8	-26.7
2	x, -y+1/2, z+1/2	7.48	-28.3	-9	-16.4	39.2	-26.6
2	-	7.39	-11.9	-3.2	-7	8	-16.1
2	-	7.58	-10.1	-5	-11.3	13.5	-15.9
2	-	8.77	-8.1	-3.7	-7.5	6.6	-13.8
2	-x+1/2, -y, z+1/2	8.09	-5.9	-0.8	-18.2	14.9	-13.5
2	-	5.73	-4.1	-2.1	-23.7	21.5	-13.3
2	-	6.56	-2.2	-2.5	-14	8.4	-11.1
1	-x, -y, -z	5.91	0.8	-1.8	-24.8	19.3	-10.1
2	x, -y+1/2, z+1/2	7.92	-2.3	-0.7	-17.3	14	-9.4
2	-	8.75	-4.1	-0.9	-15.3	16.8	-8
2	-	11.97	-3.3	-0.7	-1.1	0	-4.9
2	-x, y+1/2, -z+1/2	9.21	4.9	-1.9	-6.2	2.3	-0.2

[a] electrostatic (E_{ele}), polarization (E_{pol}), dispersion (E_{dis}), and exchange-repulsion (E_{res}). $E_{tot} = k_{ele} \cdot E_{ele} + k_{pol} \cdot E_{pol} + k_{dis} \cdot E_{dis} + k_{res} \cdot E_{res}$, with k being scale factors.

12. References

(1) Gölles, F. The examination and calculation of thermodynamic data from experimental measurements. I. The numerical integration of the vapor-pressure curves of the system methanol-water. *Monatsh. Chem* **1961**, *92*, 981-991.
(2) Zhu, H.; Yuen, C.; Grant, D. J. W. Influence of water activity in organic solvent + water mixtures on the nature of the crystallizing drug phase. 1. Theophylline. *International Journal of Pharmaceutics* **1996**, *135* (1), 151-160. DOI: https://doi.org/10.1016/0378-5173(95)04466-3.
(3) *CSD python api scripts - packing similarity dendrogram*; 2016. https://github.com/ccdc-opensource/csd-python-api-scripts/tree/main/scripts/packing_similarity_dendrogram (accessed 11.01.2024).
(4) Childs, S. L.; Wood, P. A.; Rodríguez-Hornedo, N.; Reddy, L. S.; Hardcastle, K. I. Analysis of 50 Crystal Structures Containing Carbamazepine Using the Materials Module of Mercury CSD. *Crystal Growth & Design* **2009**, *9* (4), 1869-1888. DOI: 10.1021/cg801056c.
(5) Markvardsen, A. J.; David, W. I. F.; Johnson, J. C.; Shankland, K. A probabilistic approach to space-group determination from powder diffraction data. *Acta Crystallogr. , Sect. A: Found. Crystallogr* **2001**, *A57* (1), 47-54. DOI: 10.1107/S0108767300012174.
(6) David, W. I. F.; Shankland, K.; van de Streek, J.; Pidcock, E.; Motherwell, W. D. S.; Cole, J. C. DASH: a program for crystal structure determination from powder diffraction data. *Journal of Applied Crystallography* **2006**, *39*, 910-915. DOI: 10.1107/S0021889806042117.
(7) Pawley, G. S. Unit-Cell Refinement from Powder Diffraction Scans. *Journal of Applied Crystallography* **1981**, *14* (DEC), 357-361. DOI: 10.1107/S0021889881009618.
(8) Rietveld, H. M. A Profile Refinement Method for Nuclear and Magnetic Structures. *Journal of Applied Crystallography* **1969**, *2*, 65-71. DOI: 10.1107/s0021889869006558
(9) *Topas Academic* Coelho Software: Brisbane, 2020. (accessed.
(10) Burger, A.; Ramberger, R. On the polymorphism of pharmaceuticals and other molecular crystals. II. *Microchimica Acta* **1979**, *72* (3), 273-316. DOI: 10.1007/BF01197380.
(11) Burger, A.; Ramberger, R. On the polymorphism of pharmaceuticals and other molecular crystals. I. *Microchimica Acta* **1979**, *72* (3), 259-271. DOI: 10.1007/BF01197379.
